# Polycyclic aromatic azomethine ylides: a unique entry to extended polycyclic heteroaromatics†
†Electronic supplementary information (ESI) available. CCDC 1022817 and 1022816. For ESI and crystallographic data in CIF or other electronic format see DOI: 10.1039/c4sc02793k



**DOI:** 10.1039/c4sc02793k

**Published:** 2014-10-22

**Authors:** Reinhard Berger, Manfred Wagner, Xinliang Feng, Klaus Müllen

**Affiliations:** a Max-Planck-Institut für Polymerforschung , Ackermannweg 10 , 55128 Mainz , Germany . Email: muellen@mpip-mainz.mpg.de; b Chair for Molecular Functional Materials , Technische Universität Dresden , 01062 Dresden , Germany . Email: xinliang.feng@tu-dresden.de

## Abstract

Based on polycyclic aromatic azomethine ylides (PAMYs), a metal-free “cycloaddition-planarization-sequence” is proposed, providing a unique entry to extended nitrogen-containing polycyclic aromatic hydrocarbons (N-PAHs). This method is highly versatile, as the structure of unprecedented N-PAHs can be tailored by the dipolarophile in the crucial 1,3-cycloaddition-reaction. Linear, as well as five- and six-membered cyclic dipolarophiles are successfully used. The geometric and optoelectronic nature of N-PAHs are investigated by UV-vis absorption and single crystal structure analysis. Remarkably, the newly synthesized N-PAHs demonstrate varying absorption profiles, covering the whole visible light range with rich photophysical properties, for example, fluorescent quantum yields up to 54%.

## Abstract 2

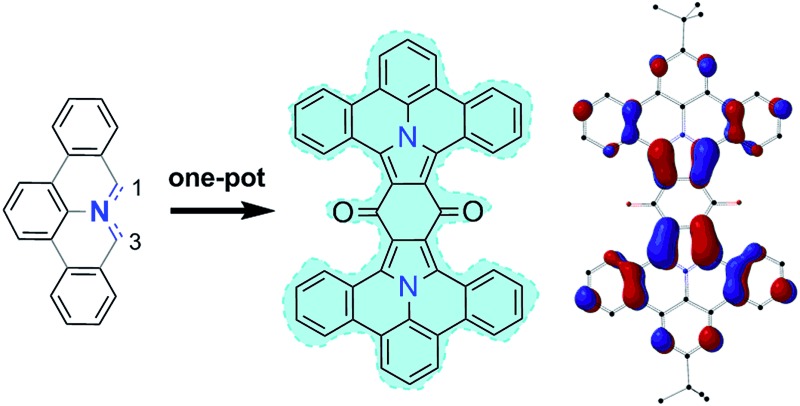
Based on polycyclic aromatic azomethine ylides, a metal-free “cycloaddition-planarization-sequence” is proposed, providing a unique entry to nitrogen-containing polycyclic aromatic hydrocarbons.

## Introduction

### Azomethine ylides and polycyclic aromatic hydrocarbons

**Fig. 1:**
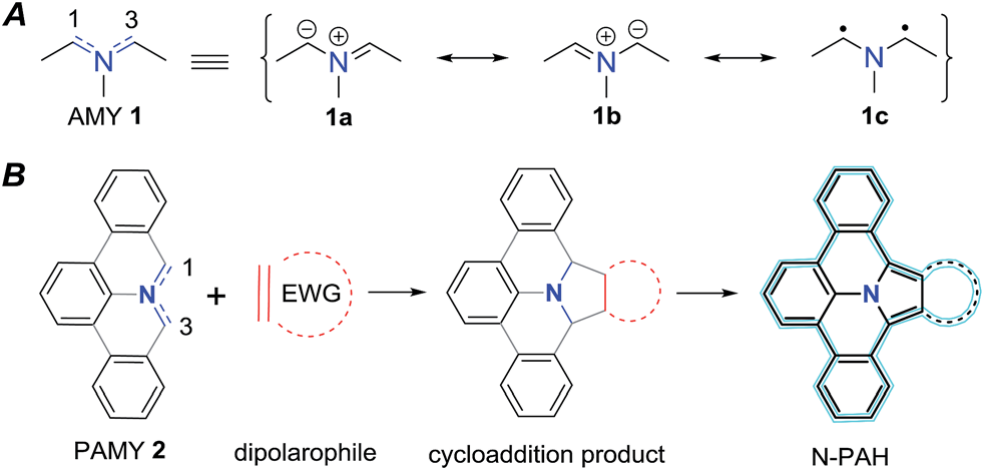
An azomethine ylide (AMY, **1**) together with its two ionic (**1a–b**) and its diradical (**1c**) Lewis structures. Schematic representation of a polycyclic aromatic azomethine ylide (PAMY, **2**) used in a 1,3-dipolar cycloaddition reaction (click-reaction) with an electron-poor dipolarophile. After oxidation of the cycloaddition product, extended π-conjugated nitrogen containing N-PAHs are obtained.

Azomethine ylides (AMY 1, Fig. 1) are prime examples of 1,3-dipolar compounds.^1,2^ Their structure is isoelectronic to that of the allyl anion with the negative charge equally distributed over two carbon atoms adjacent to the central nitrogen atom (see Fig. 1). Besides the two ionic Lewis structures (**1a** and **1b**), the diradical (**1c**) structure contributes to the overall ground state as well.^3–6^ Calculations have been used to quantify the diradical character of AMYs and to correlate it to their chemical reactivity in 1,3-dipolar cycloaddition reactions.^7,8^ In general, AMYs are highly reactive and only a few examples of stabilized AMYs have hitherto been isolated.^9–16^ The high reactivity makes AMYs essential for facile construction of five-membered heterocycles, and a variety of methods, such as deprotonation or desilylation of precursor derivatives have been developed.^17^ However, the synthesis of AMY conjugated with multiple aromatic rings (PAMY **2**) has not been described, although they are an attractive building block for the synthesis of nitrogen-containing polycyclic aromatic hydrocarbons (N-PAHs) as proposed in Fig. 1.

## Results and discussion

### 1,3-Dipolar cycloaddition reaction and planarization

**Scheme 1:**
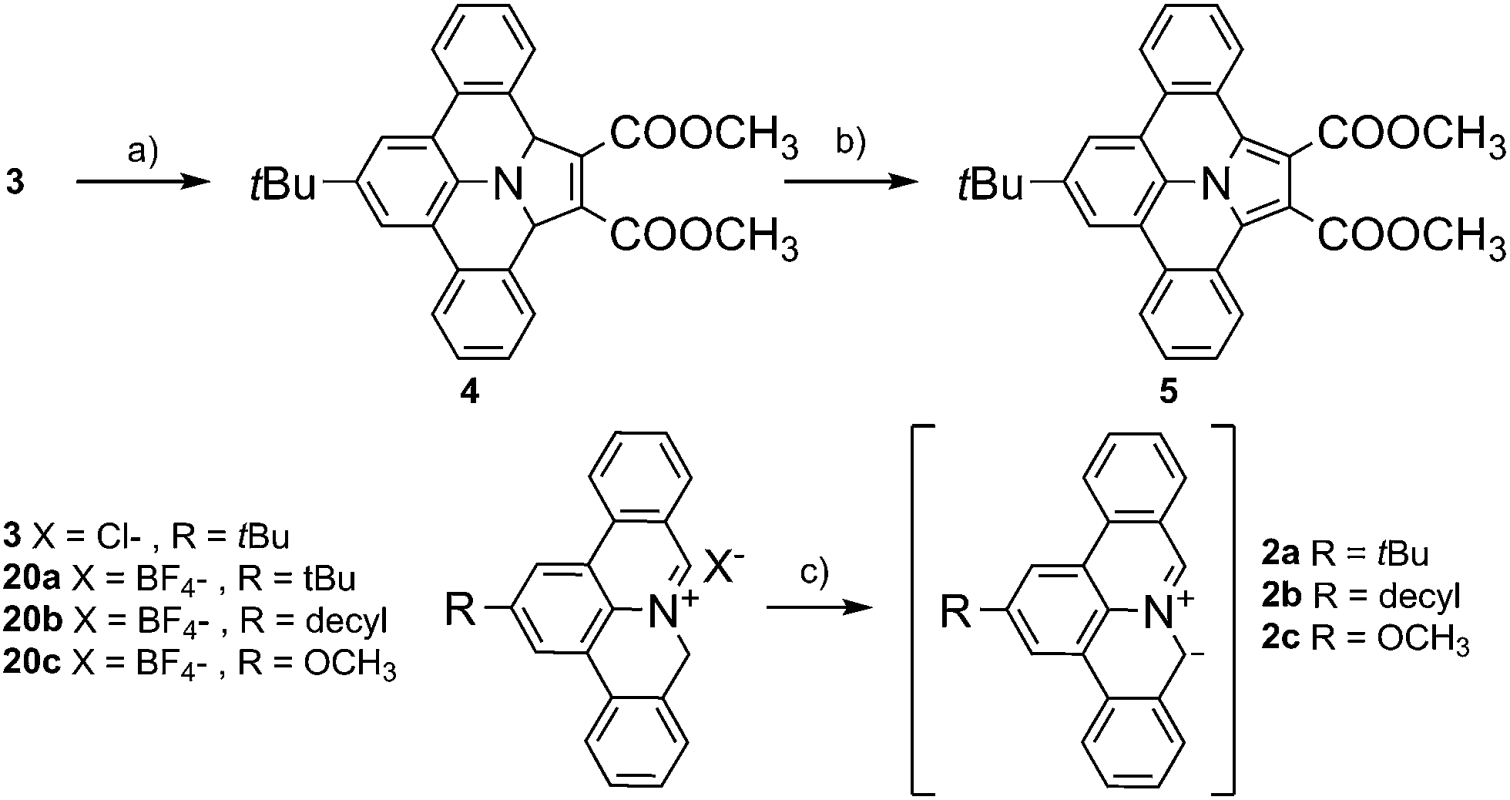
Synthetic route to N-PAH **5** based on **3**. Mild oxidation of **4** by DDQ results in planar N-PAH **5** with extended π-conjugation over the whole molecule. (a) DMAD, TEA, dichloromethane, 25 °C; (b) DDQ, toluene, 25 °C; (c) TEA, dichloromethane, 25 °C.

With our initial motif to explore biradicals based on 9a-azaphenalene,^18^ we recently found that treatment of 2-(*tert*-butyl)-8*H*-isoquinolino[4,3,2-de]phenanthridin-9-ium chloride (**3**) with triethyl amine in the presence of dimethoxy acetylene dicarboxylate (**6**, DMAD) resulted in direct formation of dimethyl-8-(*tert*-butyl)-2*a*,13*b*-dihydrobenzo[7,8]indolizino[6,5,4,3-def]phenanthridine-1,2-dicarboxylate (**4**) *via* a selective 1,3-dipolar cycloaddition reaction between DMAD and *in situ* generated PAMY **2a** (see Scheme 1). The X-ray crystallographic analysis of **4** validated the selective 1,3-addition and revealed a tilted geometry with minor π-conjugation along the *meta*-terphenylene plane. The polycyclic feature of compound **4** strongly suggests that it can be further converted to an extended N-PAH. Therefore, in this work, we subjected compound **4** to oxidative dehydrogenation by treating with 2,3-dichloro-5,6-dicyano-1,4-benzoquinone (DDQ). A fully planar, π-extended N-PAH, 8-(*tert*-butyl)-benzo[7,8]indolizino[6,5,4,3-def]phenanthridine-1,2-dicarboxylate (**5**) was obtained in 82% yield, which was unambiguously characterized by NMR, MS, and single crystal analysis. Notably, this sequence can be conducted without purification of the intermediate cycloaddition product.

**Scheme 2:**
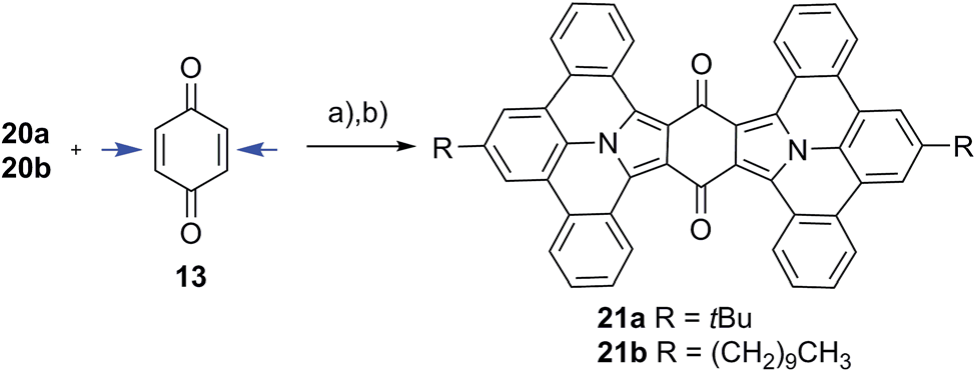
Twofold addition of PAMYs to both double bonds in **13** (indicated by blue arrows). The reaction was induced by treatment of precursor **20a** or **20b** with triethyl amine, followed by planarization with DDQ to obtain large N-PAHs **21a–b**; (a) triethyl amine, dichloromethane, 25 °C; (b) DDQ, toluene.

### Scope of reaction

**Fig. 2:**
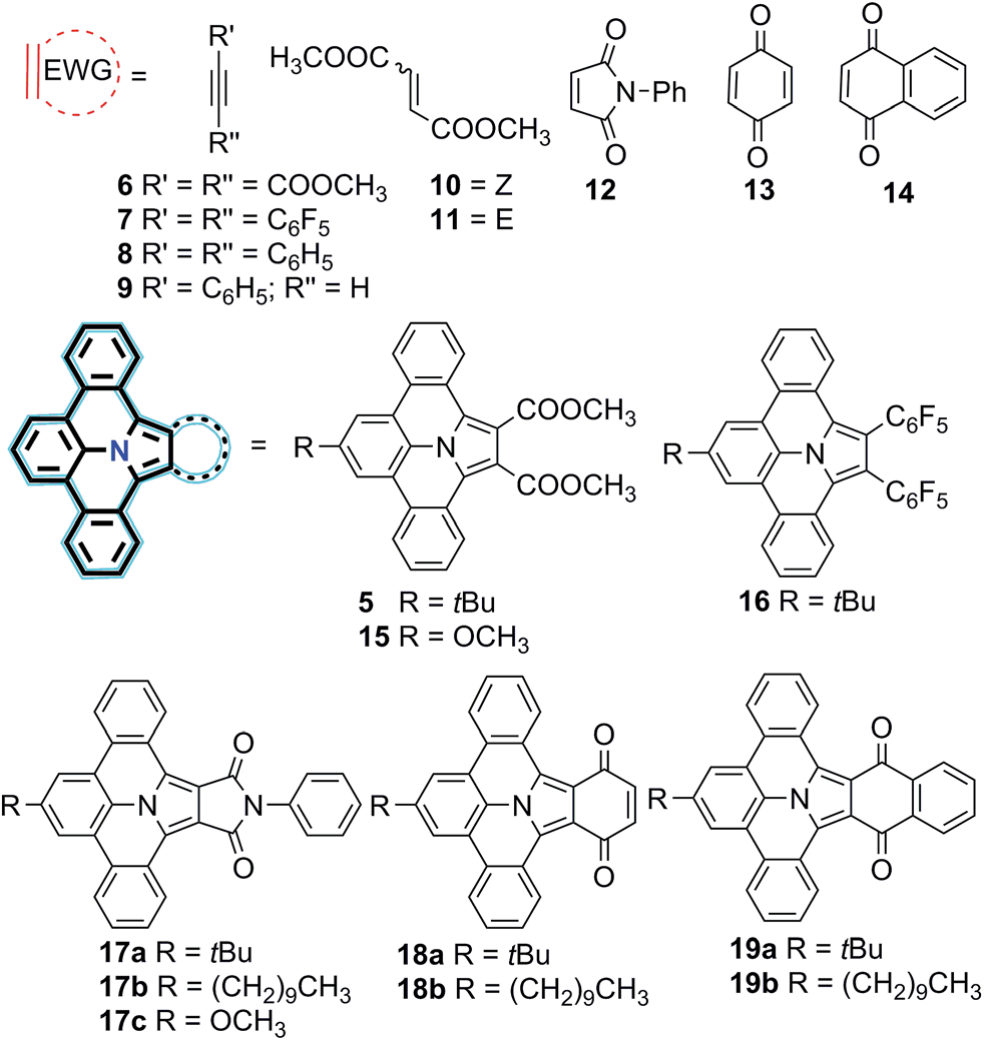
Scope of N-PAHs **15a–19b** synthesized dependent on the dipolarophiles **6–14** used in the 1,3-dipolar cycloaddition reaction.

Encouraged by the successful synthesis of N-PAH **5**, we conceive that our synthetic protocol of using PAMYs it is not only applicable to linear ethyne DMAD (**6**), but also to a series of tilted (**10**, **11**), pentacyclic (**12**) and hexacyclic ethylenes (**13–14**) as dipolarophiles. In this way, unprecedented nitrogen containing N-PAHs **15–19b** can be accessed (see Fig. 2). Moreover, the scope of this concise, metal-free reaction sequence can be further broadened by the use of other PAMYs with long alkyl chains (**2b**), or electron-donating substituents (**2c**). Like AMYs, PAMYs are highly reactive and thus need to be generated *in situ* from a suitable precursor. To obtain PAMYs **2a–c** with *tert*-butyl-, decyl- and methoxy-substituents, the corresponding precursor derivatives **20a–c** were synthesized from 2,6-dibromo-anilines (see Fig. S2, ESI†). Finally, the key step for the preparation of N-PAHs **5** and **15–19b**, the 1,3-dipolar cycloaddition reaction of *in situ* formed PAMYs with dipolarophiles **6–14**, was induced by dropwise addition of triethyl amine to solutions of the corresponding precursors **20a–c**. Afterwards, the crude cycloaddition product was directly oxidized with DDQ in toluene, to afford the planarized N-PAHs **5** and **15a–19b** in good to excellent yields of 48–95%. As shown in Table 1, the yields of N-PAHs **5**, **15–19b** are summarized for each precursor (**20a–c**) and dipolarophile (**6–14**). The linear ethyne **6**, as well as the tilted ethynylenes **10**, and **11** participated in the 1,3-dipolar cycloaddition reaction with the *in situ* generated PAMY **2a** and afforded the same N-PAH **5** after planarization. This is in agreement with our expectation that linear unsaturated carbon–carbon bonds such as ethynes and ethynylenes with electron withdrawing groups undergo 1,3-dipolar cycloaddition reaction with the electron rich PAMYs, independent of their molecular geometry. On the contrary, the electron rich ethynes such as diphenyl acetylene (**8**) and monophenyl acetylene (**9**), which are frequently used in Diels–Alder reactions of inverse electron-demand, failed to undergo the 1,3-dipolar cycloaddition reaction. This result indicates that the electronic effect has a large influence on the reactivity of cycloaddition reaction. Importantly, 1,2-bis(perfluorophenyl)ethyne (**7**) was found to be suitable as the dipolarophile, showing that other non-carbonyl electron-withdrawing groups can be used as well. In this case, the planarized N-PAH **16** is obtained as colourless crystals in 48% yield.

**Table 1:** Precursors and dipolarophiles used in the cycloaddition reactions and isolated yields of N-PAHs **15a–19b**, obtained after oxidation with DDQ

Precursor	Dipolarophile	N-PAH	Yield^*a*^ (%)
**20a**	**6**	**5**	82
	**7**	**16**	48
	**8**	—	—
	**9**	—	—
	**10**	**5**	95
	**11**	**5**	83
	**12**	**17a**	64
	**13**	**18a**	78
	**14**	**19a**	90
**20b**	**12**	**17b**	91
	**13**	**18b**	69
	**14**	**19b**	89
**20c**	**3**	**15**	68
	**9**	**17c**	74

^*a*^Isolated yield of N-PAHs after planarization.

Inspired by the successful synthesis of **5** and **16**, the cyclic five-membered dipolarophile *N*-phenylmaleimide **12** was thus examined in combination with precursor **20a** yielding N-PAH **17a** as yellow solid in 64% yield after two steps. In contrast to N-PAHs **5**, **15** and **16**, the conjugation of N-PAH **17a** is extended over an additional five-membered pyrrole cycle. Probably due to the larger conjugated structure, the solubility of N-PAH **17a** in conventional organic solvents is considerably lower than that of N-PAHs **5** and **16**. However, when **20b** was used as precursor, a decyl chain could be introduced *via* PAMY **2b** in N-PAH **17b**, which enhanced solubility compared to the *tert*-butyl substituent in N-PAH **17a**.

We further extend our synthetic protocol to larger six-membered dipolarophiles, such as 1,4-benzoquinone (**13**) and 1,4-napthoquinone (**14**). Both **13** and **14** indeed afforded N-PAHs **18a** and **18b** as red and N-PAHs **19a** and **19b** as orange powders, respectively, in 78–90% yield. Encouraged to further streamline the two-step procedure, we tested to conduct cycloaddition and oxidation in one-pot. Exemplified on **20a** as precursor and **14** as dipolarophile, N-PAH **19a** was achieved in one-pot using toluene as inert solvent (see Fig. S5, ESI†).

### Twofold addition

Compound **13** can be considered as “double-dipolarophile” and allows for a twofold 1,3-dipolar cycloaddition reaction by simply adjusting the ratio of PAMY precursor to dipolarophile to 2 : 1 (Scheme 2). After planarization with DDQ, highly extended N-PAHs **21a–b** are obtained as red powders in excellent yields of 82%. In N-PAHs **21a–b**, 48 π-electrons are delocalized throughout the molecule. For comparison, the famous PAH hexa-*peri*-hexabenzocoronene (HBC) only consists of 42 delocalized π-electrons.^19–21^ More importantly, **21a–b** contain two (pyrrolic) nitrogen atoms at the interior of the planar PAH-skeleton, which is usually synthetically much more challenging than introducing nitrogen at the more accessible peripheries.^22^ The few examples that have been reported to date, strongly suggested that N-PAHs with central nitrogen atoms may pave the way to developing new building blocks in nanoelectronics and supramolecular assemblies.^23–28^

### Structure

**Fig. 3:**
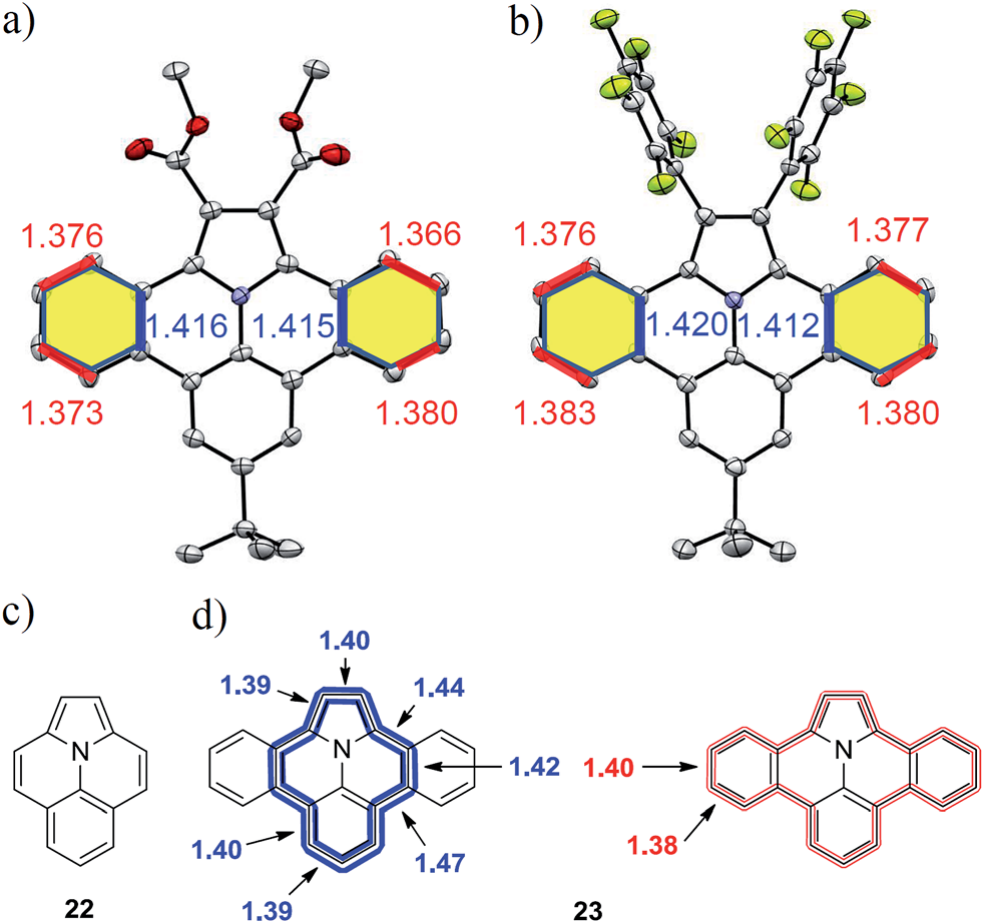
Crystal structures of N-PAHs (a) **5** and (b) **16** with selected bond lengths. Chemical structures of ullazine (**22**) and dibenzoullazine (**23**) with calculated bond length along the inner (blue) and outer (red) perimeter.

Although the solubility of the larger derivatives **21a–b** is limited to high-boiling organic solvents such as tetrachloroethane, all intermediates and precursor compounds could be analyzed by ^1^H-, ^13^C-NMR-spectroscopy and HR-ESI mass spectrometry. Single crystals of the planararized N-PAHs **5** and **16** suitable for X-ray crystallographic analysis were grown by slow evaporation of solutions in dichloromethane, which clearly revealed their fully planar structures (Fig. 3a and b).

Both compounds showed different carbon–carbon bond lengths in the annulated benzene rings (highlighted yellow). Those along the inner perimeter (marked blue) had a length of 1.41 Å, whereas a bond length of 1.38 Å was found at the outer perimeter (marked red) (Fig. 3a and b). This indicates a more pronounced double bond character in comparison to the typical bond length of 1.39 Å in benzene for the outer perimeter. On the other hand, the average bond length of 1.40 Å in the inner perimeter is in agreement with that in nitrogen containing heterocycles like pyridine (1.40 Å). Similar, the C2–C2a and C13b–C14 distances, as well part of the inner perimeter, are elongated to 1.39 Å from 1.38 Å in pristine pyrrole.^29^ Indeed, the inner perimeter of **5** and **16** can be identified as indolizino[6,5,4,3-aij]quinoline (**22**), which is an electronic isomer of pyrene with the more common name “Ullazine” based on the nomenclature of Bali and Zeller (see Fig. 3).^30^ Recently, derivatives of ullazine **22** attracted attention owing to their both electron donating and accepting properties and therefore their potential use as efficient sensitizers in dye-sensitized solar cells.^31–33^ In the dibenzoannulated derivative **23**, the ullazine core is stabilized by two additional benzenes, while the annulene character of **22** along the inner perimeter (blue) is still maintained. Therefore, we propose the name dibenzo[*d*,*k*]ullazine for N-PAH **23**, whose structural motif is found in all N-PAHs **5**, **15–19b** and twofold in **21a–b**.

### Photophysical properties

**Fig. 4:**
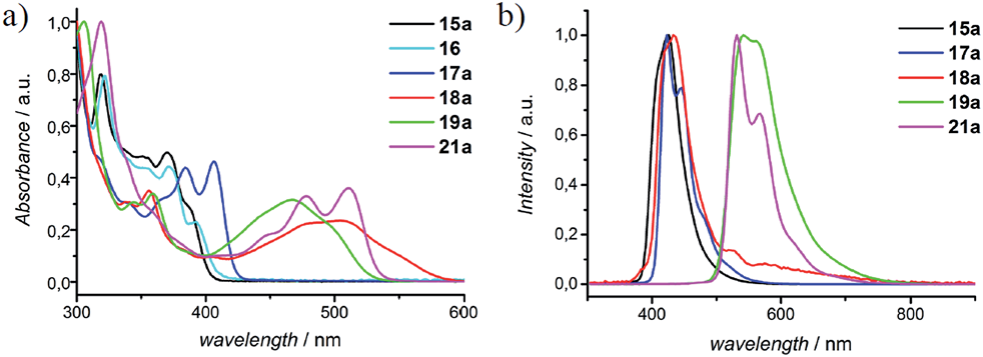
Normalized (a) UV-vis absorption and (b) fluorescence spectra of selected N-PAHs **5**, **16**, **17a**, **18a**, **19a**, and **21a**, representing the effect of annulation and/or substitution.

In Fig. 4, UV-vis absorption spectra of representative N-PAHs **5**, **16**, **17a**, **18a**, **19a**, and **21a** are displayed. N-PAHs **5** and **16** show the absorption maxima at *λ*_max_ = 388 nm and 394 nm, respectively, and both exhibit high molar extinction coefficients (*ε*) of 687 m^2^ mol^–1^. The pyrrole-annulated compound **17a** shows three pronounced transitions with a maximum absorption bathochromically shifted to *λ*_max_ = 406 nm. Annulation with quinones leads to a much stronger bathochromic shift with a broad absorption up to 600 nm for **18a** and a less shifted but more pronounced absorption maximum at *λ*_max_ = 498 nm for **19a**. In contrast to **19a**, which shows a strong fluorescence emission at 541 nm, the emission maximum of **18a** is found at 435 nm, close to that of N-PAHs **5** and **17a**. Therefore, we assume the S_1_ state of **18a** to be closer to the smaller N-PAHs **5** and **16–17a** and attribute the broad absorbance to an intramolecular charge-transfer band. Furthermore, for the large N-PAH **21a**, the absorption pattern shows three pronounced transitions with an absorption maximum at *λ*_max_ = 510 nm and a high value of *ε* = 1665 m^2^ mol^–1^. This absorption spectra resemble that of *N*,*N*′-diphenyl-3,4,9,10-perylentetracarboxylic-3,4:9,10-diimide (PDI), a benchmark system in dye chemistry and industrial pigments. Moreover, all N-PAHs **5**, **15–17a** and **21a** have good fluorescence quantum yields in a range from 14% to 54% obtained from the comparative method of Williams *et al.*, using 9,10-diphenylanthracene or Rhodamin 6G as ref. 34. To evaluate HOMO/LUMO energies of N-PAHs **15a–21a**, cyclic voltammetry was conducted using ferrocene as reference. The combined optoelectronic data are summarized in Table 2.

**Table 2:** Optical and electrochemical data

	*λ*_max_ ^*a*^ [nm]	*λ*_em_ ^*b*^ [nm]	*Φ*_F_	*E*_(0–0)_ ^*e*^ [eV]	*U* ^*f*^ [V]	*E*_HOMO_ ^*g*^ [ev]	*E*_LUMO_ ^*h*^ [ev]
**5**	388	424	0.45^*c*^	3.16	0.75	–5.55	–2.39
**15**	392	427	—	3.12	0.74	–5.54	–2.42
**16**	393	428	0.51^*c*^	3.14	0.77	–5.57	–2.50
**17a**	407	423	0.54^*c*^	3.02	0.84	–5.64	–2.50
**17c**	408	423	0.38^*c*^	3.00	—	—	—
**18a**	555	434	—	3.15	–1.38; 0.89	–5.69	–3.42^*j*^
**19a**	502	541	0.34^*d*^	2.45	–1.62; 0.85	–5.65	–3.95^*j*^
**21a**	512	532	0.14^*d*^	2.41	–1.86	–5.30^*i*^	–2.94^*j*^

^*a*^ *λ*_max_: absorption maximum at longest wavelength.

^*b*^ *λ*_em_: emission wavelength.

^*c*^Obtained from the comparative method of Williams *et al.*, using 9,10-diphenylanthracene as reference at excitation wavelength *λ*_ex_ = 380 nm.

^*d*^Rhodamin 6G at *λ*_ex_ = 470 nm. 9,10-diphenylanthracene as standard at excitation wavelength *λ*_ex_ = 325 nm.

^*e*^Measured at the intersection of the normalized absorbance and emission spectra.

^*f*^Redox potentials from CV are reported *vs.* Fc/Fc+ (0.1 M nBu_4_NPF_6_ in CH_3_CN), scan rate 50 mV s^–1^.

^*g*^HOMO values were derived from the first measured oxidation potential.

^*h*^LUMO values were evaluated by *E*_LUMO_ = *E*_HOMO(CV)_ + *E*_(0–0)_.

^*i*^HOMO value was evaluated by *E*_HOMO_ = *E*_LUMO(CV)_ – *E*_(0–0)_.

^*j*^LUMO values were derived from the first measured reduction potential.

### Density functional calculations

**Fig. 5:**
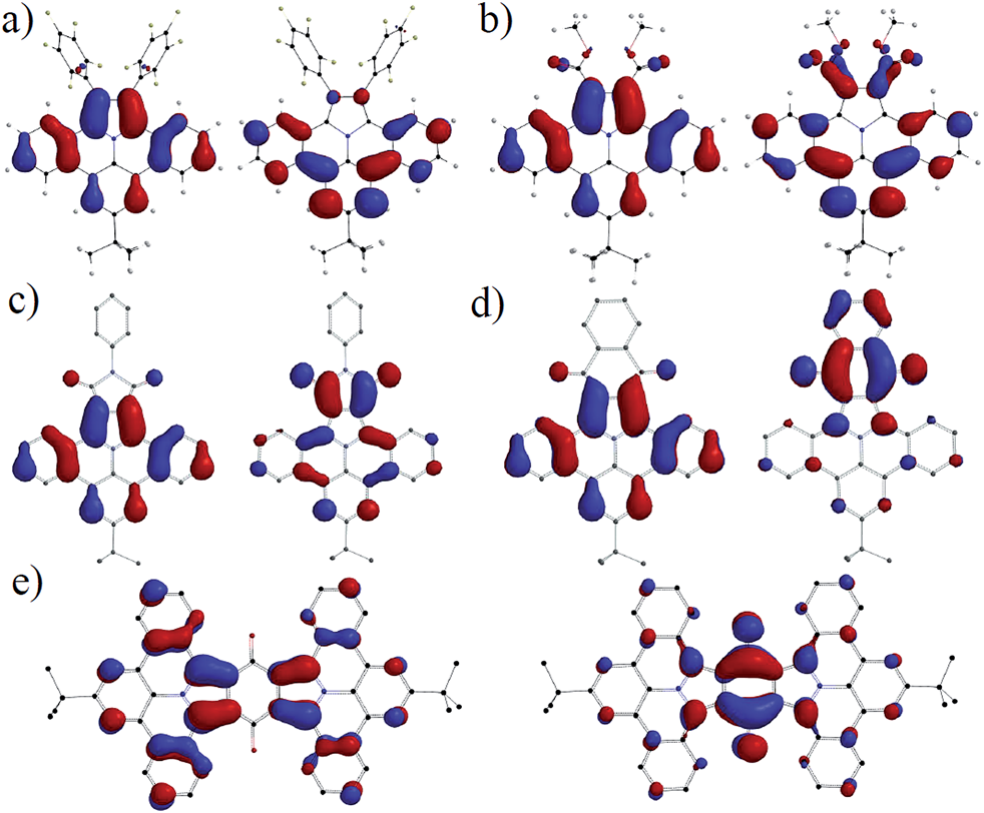
Graphical representation of HOMOs (left) and LUMOs (right) of N-PAHs (a) **16**, (b) **5**, (c) **17a**, (d) **19a** and (e) **21a**. The calculations were performed with DFT B3LYP 6-31g(d,p) based on geometries derived from crystal structure data obtained for **15a** and **16**.

To relate the optical and geometrical properties of N-PAHs **5**, **15–19b** and **21** to their electronic ground state, we performed density functional theory (DFT) with Gaussian09 using b3lyp functional on 6-31g(d,p) level.^35,36^ The geometries of **17a–19a**, **21a** and **23** were derived from the crystal structures of **16**, optimized on AM1 and computed with DFT b3lyp at the 6-31g(d,p) level. The graphical representations of the highest occupied molecular orbital (HOMO) and the lowest unoccupied molecular orbital (LUMO) in Fig. 5 both show a bisecting nodal plane, through the 8-position and the nitrogen atom for all N-PAHs. For comparison, ullazine **22** possesses such a nodal plane only in the HOMO.^33,37^ As a result, substituents introduced in the 8-position such as *tert*-butyl, decyl, and methoxy do not exert an influence on the optoelectronic properties, which is in agreement with the experimental results.

In stark contrast, substitution and/or annulation in the 1,2-position for **5**, **16**, **17a**, **19a** and **21**a lead to a redistribution in the orbital landscape. For N-PAHs **5** and **16**, HOMO and LUMO are located on the main part of the molecule similar to those of the parent molecule **23** and only little coefficients are located on the pentafluorophenyl substituents. The highly planar nitrogen containing heterocycle of the ullazine family, consisting of 24 delocalised π-electrons, can be considered as a donor molecule, which will facilitate a strong intramolecular charge-transfer if an acceptor is present. By introducing carbonyl groups such as carboxylic esters in **5**, the LUMO is shifted from the main core of dibenzoullazine **23** to the part of the molecule containing the electron-withdrawing groups. However, the free rotation around the C–C bond causes only minor conjugation over theses substituents in the HOMO and the LUMO. Therefore, no charge transfer can be detected and the *λ*_max_ for **5**, **15** and **16** is closely around 390 nm. This situation changes if the 1,2-substituents are forced into conjugation by formation of five-(**17a–c**)- or six-(**18a–19b**)-membered rings. Here an efficient charge-transfer can be observed. The HOMO is localized on the donor part while the LUMO relies exclusively on the acceptor (five-, six-membered ring) unit. This resembles a classic donor–acceptor (D–A) pattern and a dipole is induced along the nodal plane of the molecule with increasing acceptor strength. The increasing dipole moment and the charge transfer are origin of the bathochromic shift of *λ*_max_ ranging from ∼20 nm for **17a** to ∼170 nm (**18a**) compared to **5**. In **21a** the HOMO is located on two donor units (D–A–D), while the LUMO is centered on the middle quinone core. A charge transfer occurs from both sides. The extension of the overall conjugated system explains an additional bathochromic shift of 10 nm for **21a** compared to **19a**. The high symmetry of the HOMO and LUMO in combination with a certain rigidity of the planar core in **17a** might explain the high fluorescent quantum yields, because of large transfer integral and low relaxation of the excited state *via* motion.

## Conclusions

In summary, we established the use of planar polycyclic aromatic azomethine ylides (PAMYs) and demonstrated their versatile potential in the construction of large nitrogen containing polycyclic aromatic hydrocarbons for the first time. We provide a general approach to PAMYs, exemplified for three differently substituted pentacyclic derivatives based on readily available 2,6-dibromo-anilines. By cycloaddition reaction with a series of dipolarophiles and subsequent oxidation of the intermediate, it was possible to synthesize six classes of novel N-PAHs in a one-pot reaction, which all show good optoelectronic properties, such as high extinction coefficients and fluorescence quantum yields up to 54%. These unprecedented N-PAHs can further be functionalized in various ways giving access to promising materials, for example as organic sensitizers for solar-cell application.^33^
