# Supplementary material for: Polycyclic aromatic azomethine ylides: a unique entry to extended polycyclic heteroaromatics
Source: Chem Sci. 2014 Oct 22;6(1):436–41. doi: 10.1039/c4sc02793k (PMC5588116; doi:10.1039/c4sc02793k)
Supplement: Supplementary file 1 [file SC-006-C4SC02793K-s001.pdf]

Electronic Supplementary Material (ESI) for Chemical Science.
This journal is © The Royal Society of Chemistry 2014

Electronic Supplementary Information (ESI)

Polycyclic Aromatic Azomethine Ylides: A Unique Entry to Extended Polycyclic Heteroaromatics**

Reinhard Berger, Manfred Wagner, Xinliang Feng,* Klaus Müllen*

Contents

1) Experimental Section 1
2) X-ray crystallographic analysis 15
3) Calculations 18
4) UV-vis absorption spectra 18
5) Cyclic Voltammetry 24
6) NMR-spectra 28
7) References 46

1) Experimental Section

General Information

Unless otherwise stated, the commercially available reagents and dry solvents were used without further purification. The reactions were performed using standard vacuum-line and Schlenk techniques, work-up and purification of all compounds were performed under air and with reagent-grade solvents. For microwave assisted reactions a CEM Discover-SP w/activent 909155 was used. Column chromatography was done with silica gel (particle size 0.063-0.200mm from Macherey-Nagel) and silica coated aluminum sheets with fluorescence indicator from Macherey-Nagel were used for thin layer chromatography. The ^{1}H-NMR and ^{13}C-NMR spectra were recorded on a Bruker AVANCE 300, Bruker AVANCE III 500, Bruker AVANCE III 700 and Bruker AVANCE III 850 spectrometer in the listed deuterated solvents. The temperature was kept at 298.3 K and calibrated with a standard ^{1}H methanol NMR sample. The control of the temperature was realized with a VTU (variable temperature unit) and an accuracy of +/- 0,1K, which was monitored with the standard Bruker Topspin 3.1 software. Trimethylsilane (δ 0.00 ppm) or deuterated solvent was used as an internal standard. A standard ^{1}H NMR spectrum was measured with 64 transients and a relaxation time of 5 s. The carbon spectra were kept with a J-modulated spin-echo for ^{13}C-nuclei coupled to ^{1}H to determine the number of attached protons with decoupling during acquisition. All ^{13}C NMR measurements were done with 4096 number of scans. Solution UV-vis absorption and emission spectra were recorded at room temperature on a Perkin-Elmer Lambda 900 spectrophotometer and J&MTIDAS spectrofluorometer. High-resolution electrospray ionization mass spectrometry was performed on a QTof Ultima 3 (micromass/Waters).

General procedure for the Suzuki coupling of dibromo anilines 24a – 24c and 1-hydroxy-3*H*-2,1-benzoxaborole to afford compounds 25a – 25c

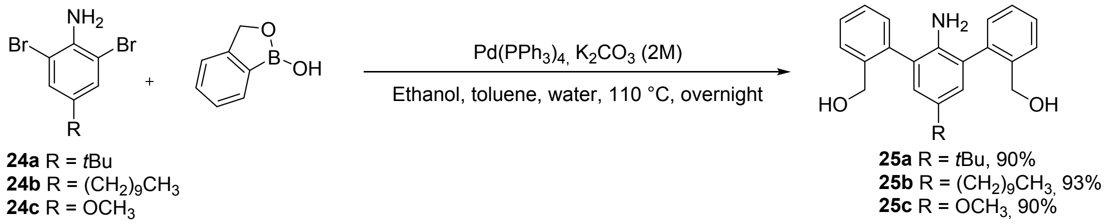

Figure S1. Synthesis of 2,6-di(1'-hydroxmethylphenyl)-anilines **25a** – **25c**.

A solution of the respective 2,6-dibromoaniline **24a** – **24c** (3.26 mmol, 1.00 eq.) and 1-hydroxy-3H-2,1-benzoxaborole (1.30 g, 9.78 mmol, 3.00 eq.) in a mixture of toluene (80 mL), ethanol (16 mL) and 2 M potassium carbonate solution (31 mL) was purged with argon for 30 min. After tetrakis(triphenylphosphine)palladium(0) (0.38 g, 0.32 mmol , 10 mol%) was added, the mixture was refluxed in a preheated oil bath (110 °C) overnight. The reaction mixture was allowed to reach room temperature and the organic layer was separated. The aqueous phase was extracted with diethylether (50 mL, three times) and the combined organic layers were washed with brine and dried over magnesium sulfate afterwards. The solvent was removed under reduced pressure and the residue was purified by column chromatography on silica (hexane:ethyl acetate; 1:2) to afford the title compound **25a** - **25c**.

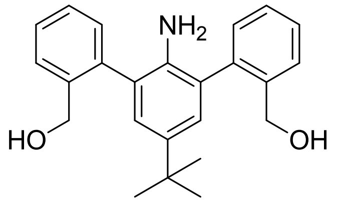

2,6-Di(1'-hydroxmethylphenyl)-4-*tert*-butyl-aniline (**25a**)

^{1}H NMR (300 MHz, DMSO-d$_6$, 298 K) δ 7.64-7.61 (br, 2H), 7.43-7.28 (br, 4H), 7.21-7.17 (br, 2H), 6.93 (br, 2H), 5.10-5.01 (br, 2H), 4.43-4.27 (br, 4H), 3.53-3.49 (br, 2H);^{1}H NMR (500 MHz, DMSO-d$_6$, 373 K) δ 7.64 (d, J = 7.5 Hz, 2H), 7.39 (t, J = 7.5 Hz, 2H), 7.33 (t, J = 7.5 Hz, 2H), 7.20 (d, J = 7.5 Hz, 2H), 6.96 (s, 2H), 4.60 (s, 2H), 4.38 (s, 4H), 3.42 (s, 2H), 1.28 (s, 9H);^{13}C NMR (75 MHz, DMSO-d$_6$, 298 K) δ 140.90, 140.65, 139.01, 138.92, 138.89, 137.26, 137.06, 129.75, 129.55, 127.40, 127.12, 126.86, 126.66, 125.86, 125.74, 125.14, 60.54, 60.31, 33.57, 31.44;^{13}C NMR (126 MHz, DMSO-d$_6$, 373 K) δ 140.30, 138.71, 138.37, 137.02, 129.05, 126.75, 126.26, 125.28, 125.10, 60.24, 32.98, 30.86. (hydrogen bonds

between amino and hydroxyl substituents hinder free rotation of the phenyl substituents. This causes isomer formation and explains the complex ^{1}H- and ^{13}C-spectra at room temperature); HRMS (ESI, *m/z*): calcd for $C_{24}H_{28}NO_2$ $[M+H]^+$362.2120, found 362.2120.

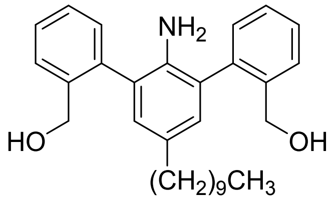

2,6-Di(1'-hydroxmethylphenyl)-4-*n*-decyl-aniline (**25b**)

^{1}H NMR (300 MHz, DMSO-d_6) δ 7.62 (d, 2H), 7.42-7.29 (br, 4H), 7.18-7.14 (br, 2H), 6.73 (s, 2H), 5.10-5.01 (br, 2H), 4.50-4.27 (br, 4H), 3.50-3.47 (br, 2H), 1.54 (t, 2H), 1.32-1.16 (br, 16H), 0.84 (t, 3H); ^{13}C-NMR (300 MHz, DMSO-d_6) δ 140.91, 140.64, 139.20, 139.12, 136.97, 136.77, 130.43, 130.24, 129.66, 129.47, 128.88, 128.81, 127.41, 127.02, 126.81, 126.61, 125.59, 60.51, 60.30, 34.15, 31.27, 31.12, 31.07, 29.00, 28.97, 28.82, 28.68, 28.56, 22.07 (hydrogen bonds between amino and hydroxyl substituents hinder free rotation of the phenyl substituents. This causes isomer formation and explains the complex ^{1}H- and ^{13}C-spectra at room temperature); HRMS (ESI, *m/z*): calcd for $C_{30}H_{40}NO_2$ $[M+H]^+$ 446.3059, found 446.3062.

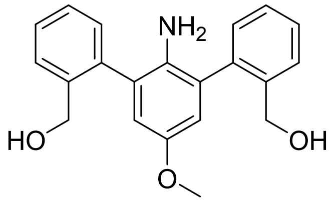

2,6-Di(1'-hydroxmethylphenyl)-4-methoxy-aniline (**25c**)

^{1}H NMR (300 MHz, DMSO-d_6, 298 K) δ 7.61 (d, J = 7.6 Hz, 2H), 7.41 (t, J = 7.4 Hz, 2H), 7.33 (t, J = 7.4 Hz, 2H), 7.17 (d, J = 7.4 Hz, 2H), 6.55 (s, 2H), 5.17 – 5.01 (br, 2H), 4.50 – 4.24 (br, 4H), 3.67 (s, 3H), 3.28 (br, J = 8.1 Hz, 2H); ^{13}C NMR (75 MHz, DMSO) δ 140.81, 140.52, 136.80, 136.60, 135.20, 129.63, 129.42, 127.60, 126.94, 126.82, 114.70, 114.64, 60.54, 60.34, 55.22; (hydrogen bonds between amino and hydroxyl substituents hinder free rotation of the phenyl substituents. This causes isomer formation and explains the complex ^{1}H- and ^{13}C-spectra at room temperature); HRMS (ESI, *m/z*): calcd for $C_{21}H_{22}NO_3$ $[M+H]^+$ 336.1600, found 336.1599.

General procedure for the preparation of precursor derivatives 20a – 20c from 25a - c

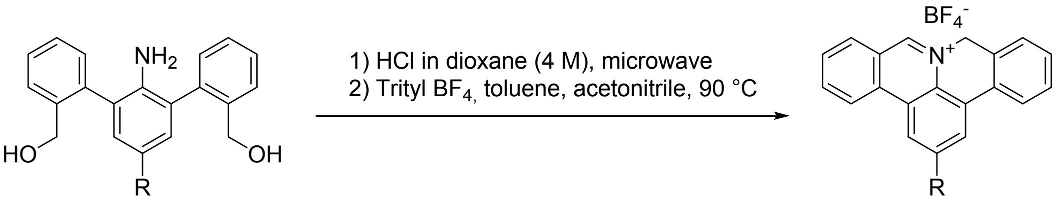

25a R = *t*Bu
25b R = $(CH_2)_9CH_3$
25c R = OCH_3

20a R = *t*Bu; 80 %
20b R = $(CH_2)_9CH_3$; 89 %
20c R = OCH_3; 79%

Figure S2. Synthesis of PAMY-percursors **20a** – **20c**

In a microwave tube, the respective compound (**25a** – **25c**) (1.46 mmol, 1.00 eq.) was added into a stirring anhydrous hydrogen chloride solution (4M in dioxane, 5 mL). The microwave tube was capped and placed in a microwave reactor. A dynamic mode was chosen (300 W, power max: on, activated cooling, pre-stirring: 10 seconds, temperature: 130 °C) for 90 minutes. After cooling to room temperature the cap was removed and the reaction mixture was transferred to a round bottom flask. The solvents were removed under reduced pressure. The crude product was redissolved in toluene (anhydrous, 10 mL) and heated to 90 °C under argon. Then a solution of triphenylcarbenium tetrafluoroborate (0.54 g, 1.63 mmol, 1.10 eq.) in anhydrous acetonitrile (5 mL) was added dropwise. Stirring is continued for 30 min and solvents were removed under reduced pressure. The residue was dissolved in a minimum amount of DCM (~10 mL) and precipitated in hexane (250 mL). The crude product was washed with a mixture of hexane and DCM (100mL, 9:1) and precipitated again to obtain the title compounds (**20a** – **20c**) as yellow solids. Yields over two steps are given in Figure S2.

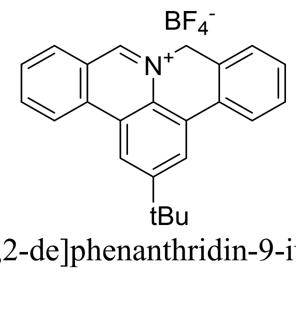

2-(*tert*-Butyl)-8H-isoquinolino[4,3,2-de]phenanthridin-9-ium tetrafluoroborate (**20a**)

^{1}H NMR (300 MHz, DMSO-d_6) δ 10.20 (s, 1H), 9.29 (d, J = 8.5 Hz, 1H), 8.93 (s, 1H), 8.76 (s, 1H), 8.62 (d, J = 7.6 Hz, 1H), 8.51 – 8.37 (m, 2H), 8.13 (t, J = 7.5 Hz, 1H), 7.69 – 7.50 (m, 3H), 6.22 (s, 2H), 1.58 (s, 9H); ^{13}C NMR (126 MHz, DMSO) δ 154.02, 153.25, 137.72, 134.03, 132.40, 130.50, 129.85, 129.18, 128.33, 128.19, 127.13, 126.45, 126.24, 126.01, 124.34, 124.06, 123.77, 123.55, 119.88, 56.93, 35.87, 30.92; Elemental Analysis for chemical

formula $C_{24}H_{22}BF_4N$: C, 70.09; H, 5.39; B, 2.63; F, 18.48; N, 3.41; HRMS (ESI, *m/z*): calcd for $C_{24}H_{22}N$ $[M-BF_4]^+$ 324.1752, found 324.1743.

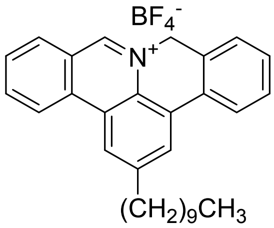

2-(*n*-Decyl)-8H-isoquinolino[4,3,2-de]phenanthridin-9-ium tetrafluoroborate (**20b**)

^{1}H NMR (300 MHz, DMSO-d$_6$) δ 10.17 (s, 1H), 9.16 (d, *J* = 8.4 Hz, 1H), 8.92 (s, 1H), 8.66 (s, 1H), 8.61 (d, *J* = 7.6 Hz, 1H), 8.38 (q, *J* = 8.0 Hz, 2H), 8.11 (t, *J* = 7.5 Hz, 1H), 7.71 – 7.49 (m, 3H), 6.22 (s, 2H), 3.09 – 2.94 (t, 2H), 1.83 (q, 2H), 1.31 (m, *J* = 36.0 Hz, 14H), 0.91 – 0.76 (m, 3H); ^{13}C NMR (75 MHz, DMSO) δ 153.01, 146.08, 137.69, 133.85, 132.32, 130.47, 129.85, 129.13, 128.28, 126.98, 126.43, 126.33, 126.22, 124.07, 123.98, 123.53, 123.12, 56.94, 35.38, 31.28, 30.84, 29.00, 28.99, 28.83, 28.74, 28.68, 22.07, 13.94; Elemental Analysis for $C_{30}H_{34}BF_4N$: C, 72.73; H, 6.92; B, 2.18; F, 15.34; N, 2.83; Found: C, 71.65; H, 6.32; N, 2.82; HRMS (ESI, *m/z*): calcd for $C_{30}H_{34}N$ $[M-BF_4]^+$ 408.2691, found 408.2700.

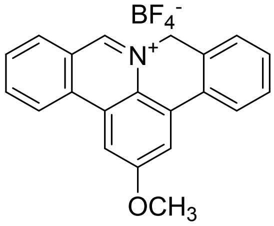

2-Methoxy-8H-isoquinolino[4,3,2-de]phenanthridin-9-ium tetrafluoroborate (**20c**)

^{1}H NMR (300 MHz, DMSO-d$_6$) δ 10.07 (s, 1H), 9.22 (d, *J* = 8.5 Hz, 1H), 8.60 (d, *J* = 8.0 Hz, 1H), 8.46 (d, *J* = 2.5 Hz, 1H), 8.42 – 8.32 (m, 3H), 8.12 (t, *J* = 7.8 Hz, 1H), 7.63 – 7.54 (m, 3H), 6.22 (s, 2H), 4.17 (s, 3H); ^{13}C NMR (176 MHz, DMSO) δ 160.77, 150.97, 137.24, 133.43, 132.13, 130.72, 130.20, 129.20, 128.59, 128.47, 128.25, 126.67, 126.40, 124.98, 124.40, 124.15, 123.98, 115.30, 105.22, 56.95, 56.68; Elemental Analysis for chemical formula $C_{21}H_{16}BF_4NO$: C, 65.49; H, 4.19; B, 2.81; F, 19.73; N, 3.64; O, 4.15; Found: C, 65.09; H, 3.86; N, 3.58; HRMS (ESI, *m/z*): calcd for $C_{21}H_{16}NO$ $[M-BF_4]^+$ 298.1221, found 298.1221.

Planarization of initial cycloaddition product 4 into N-PAH 5

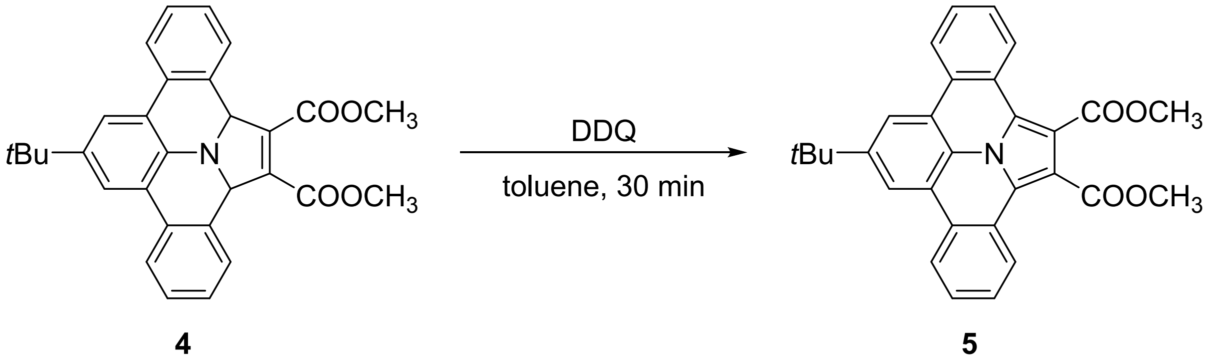

4 **5**

Figure S3. Oxidation of cycloaddition product **4** by DDQ results in targeted compound **5**.

8-(*tert*-Butyl)-benzo[7,8]indolizino[6,5,4,3-def]phenanthridine-1,2-dicarboxylate (**5**)
A dry and inert 25 mL Schlenk tube was charged with initial cycloaddition product **4** (0.10 g, 0.21 mmol, 1.00 eq.) and DDQ (0.06 g, 0.28 mmol, 1.30 eq.). The Schlenk tube was sealed with a septum and evacuated and refilled with argon three times. Afterwards anhydrous toluene (2 mL, purged with argon) was added and the reaction mixture was stirred for 30 min. The reaction mixture was filtered over a small plug of silica and the product was eluted with a mixture of ethyl acetate/DCM (2/1) and pure ethyl acetate till no fluorescence was observed in the filtrate. After removal of the solvents, the title compound **5** was obtained as a slight yellow powder (0.081 g, 0.17 mmol, 82%).

^{1}H NMR (300 MHz, DCM-d_2) δ 8.72 – 8.62 (m, 2H), 8.46 (s, 2H), 8.46 – 8.40 (m, 2H), 7.64 – 7.51 (m, 4H), 4.02 (s, 4H), 1.57 (s, 9H); ^{13}C NMR (75 MHz, DCM-d_2) δ 167.62, 148.53, 129.11, 128.57, 127.52, 125.77, 125.22, 124.58, 123.35, 123.03, 118.89, 113.50, 53.03, 35.89, 32.00; HRMS (ESI, *m/z*): calcd for $C_{30}H_{25}NO_4$ $[M+H]^+$ 486.1681, found 486.1682.

General procedure for the cycloaddition-planarization sequence for N-PAHs 15b – 19b

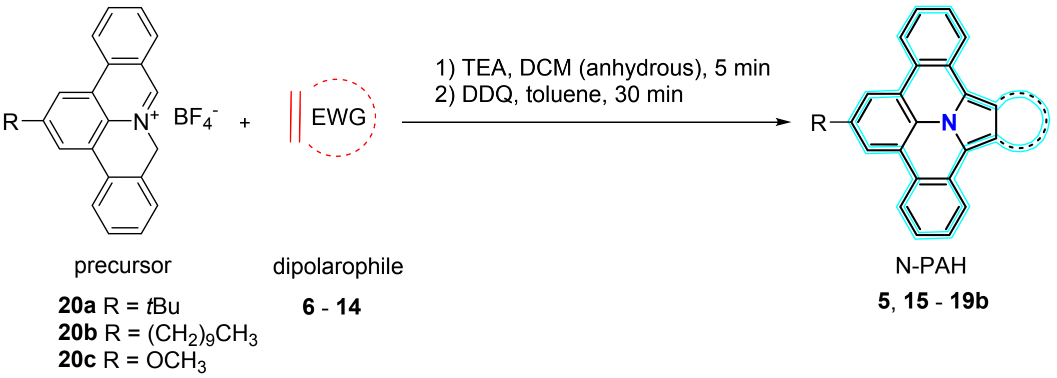

Figure S4. General reaction scheme for the two-step, cycloaddition and planarization, synthesis of N-PAH **5**, **15** – **19b**.

In a dry and inert 25 mL Schlenk tube the respective precursor **20a** – **20c** (0.10 mmol, 1.00 eq.) and the corresponding dipolarophile **6** - **14** (0.12 mmol, 1.20 eq.) were dissolved (**20b**)/suspended (**20a**, **20c**) in DCM (anhydrous, Ar bubbled, 4 mL). Under vigorous stirring triethylamine (anhydrous, Ar bubbled, 0.25 mL, ~12.00 eq) was added in one shot. The reaction was stirred for several minutes and transferred to a round bottom flask afterwards. Solvents and residual triethyl amine were removed under reduced pressure to obtain the crude product. DDQ (30 mg, 0.13 mmol, 1.30 eq.) was added and the flask was sealed with a septum and evacuated and refilled with argon three times. Toluene (4 mL, anhydrous, argon purged) was added via a syringe. The reaction was quenched by addition of water (10 mL) after 30 min and purified either by filtration over Alox (**5**, **16** and **17b**) or by recrystallization of precipitate from ethanol (**15**, **17a**, **17c**, **18a** – **19b**).

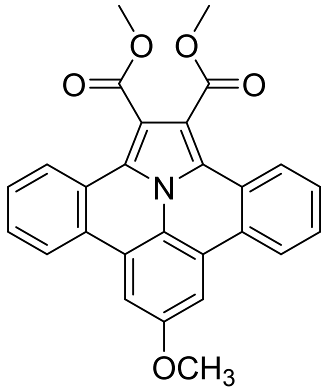

Dimethyl 8-methoxy-benzo[7,8]indolizino[6,5,4,3-def]phenanthridine-1,2-dicarboxylate (dimethyl-8-methoxy-dibenzo[d,k]ullazine-1,2-dicarboxylate, **15**)

Crude product of **15** was purified by recrystallization from ethanol.

^{1}H NMR (300 MHz, Chloroform-d) δ 8.67 – 8.57 (m, 2H), 8.20 – 8.05 (m, 3H), 7.62 (s, 2H), 7.53 – 7.43 (m, 4H), 4.05 (s, 6H), 3.95 (s, 3H); ^{13}C NMR (75 MHz, $C_2D_2Cl_4$) δ 167.38, 157.04, 129.29, 128.39, 126.42, 125.40, 124.82, 124.46, 123.89, 123.18, 112.90, 106.75, 56.11, 53.05; HRMS (ESI, *m/z*): calcd for $C_{27}H_{19}NO_5$ $[M+H]^+$ 460.1161, found 460.1164.

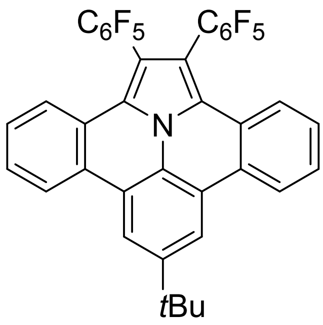

1,2-perflourphenyl-8-(*tert*-butyl)-dibenzo[d,k]ullazine (**16**)

Crude product of **16** was purified on alox.

^{1}H NMR (700 MHz, CD_2Cl_2) *δ* 8.52 (s, 2H), 8.50 (d, 2H), 7.56 (t, 2H), 7.47 (2H), 7.41 (t, 2H), 1.60 (s, 9H); ^{13}C NMR (176 MHz, CD_2Cl_2) δ 148.32, 146.45, 145.04, 139.15, 137.79, 129.36, 127.98, 127.87, 127.23, 125.78, 123.98, 123.88, 122.89, 122.69, 118.95, 110.60, 105.30, 35.91, 32.03.

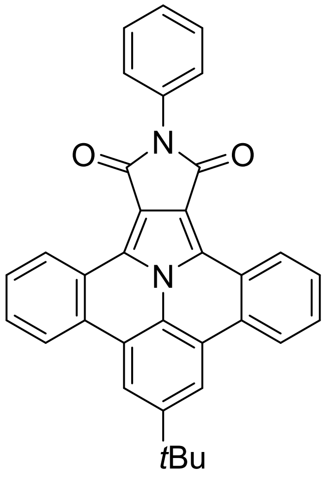

N-phenyl-8 -(*tert*-butyl)-1,2-dibenzo[d,k]ullazine imide (**17a**)

Crude product of **17a** was recrystallized from ethanol.

^{1}H NMR (300 MHz, $C_2D_2Cl_4$) *δ* 9.02 (dt, *J* = 7.3, 3.2 Hz, 2H), 8.37 (s, 2H), 8.32 (dd, *J* = 6.4, 2.9 Hz, 2H), 7.63 (dt, *J* = 6.2, 3.3 Hz, 4H), 7.49 (d, *J* = 3.1 Hz, 4H), 7.44 – 7.33 (m, 1H), 1.50 (s, 9H); ^{13}C NMR (176 MHz, C2D2Cl4) *δ* 164.27, 149.10, 133.20, 130.04, 129.73, 129.29, 128.01, 127.83, 127.57, 127.53, 127.33, 124.74, 124.24, 123.01, 122.94, 119.19, 114.45, 35.74, 32.01; Elemental Analysis for Chemical Formula $C_{34}H_{24}N_2O_2$: C, 82.91; H, 4.91; N,

5.69; O, 6.50; Found: C, 82.82; H, 4.83; N, 5.6; HRMS (ESI, *m/z*): calcd for $C_{34}H_{25}N_2O_2$ $[M+H]^+$ 493.1916, found 493.1924.

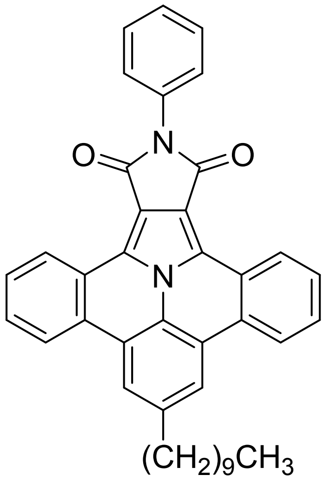

N-phenyl-8 -(*n*-decyl)-1,2-dibenzo[d,k]ullazine imide (**17b**)

Crude product of **17b** was recrystallized from ethanol.

^{1}H NMR (500 MHz, 393 K) δ 9.21 (d, J = 7.9 Hz, 2H), 8.38 (d, J = 8.1 Hz, 2H), 8.24 (s, 2H), 7.68 (br, 4H), 7.61 – 7.47 (m, 4H), 2.98 (t, J = 7.8 Hz, 2H), 1.87 (br, 2H), 1.35 – 1.21 (m, 14H), 0.93 – 0.82 (m, 3H); ^{13}C NMR (126 MHz, C_2D2Cl_4) δ 164.09, 151.60, 144.57, 142.58, 142.26, 141.04, 129.74, 129.64, 128.95, 128.01, 127.48, 124.69, 124.65, 123.65, 122.93, 121.98, 115.10, 36.79, 32.00, 31.69, 29.71, 29.71, 29.63, 29.53, 29.37, 22.69, 13.99; HRMS (ESI, *m/z*): calcd for $C_{40}H_{37}N_2O_2$ $[M+H]^+$ 577.2855, found 577.2840.

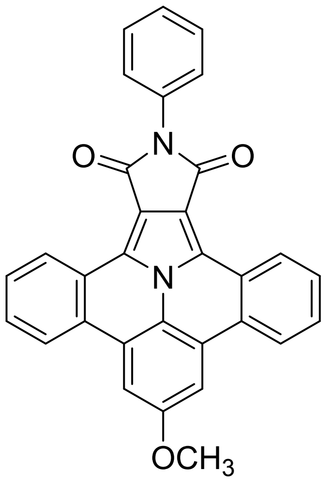

N-phenyl-8 -methoxy-1,2-dibenzo[d,k]ullazine imide (**17c**)

Crude product of **17c** was recrystallized from ethanol.

^{1}H NMR (500 MHz, C_2D2Cl_4) *δ* 9.12 (d, *J* = 7.8 Hz, 2H), 8.23 (d, *J* = 8.1 Hz, 2H), 7.84 (s, 2H), 7.64 (m, 4H), 7.56 (d, *J* = 7.9 Hz, 2H), 7.51 (t, *J* = 7.6 Hz, 2H), 7.38 (t, *J* = 7.4 Hz, 1H), 4.04 (s, 3H); ^{13}C NMR (126 MHz, C_2D2Cl_4) *δ* 164.05, 158.07, 133.75, 129.87, 129.69, 129.01, 127.93, 127.65, 127.45, 126.98, 124.99, 124.09, 122.92, 122.92, 114.94, 107.90, 56.36; HRMS (ESI, *m/z*): calcd for $C_{31}H_{19}N_2O_3$ $[M+H]^+$ 467.1396, found 467.1383; Due to the low solubility of **17c** in acetonitrile, chloroform and TFA had to be added for HRMS.

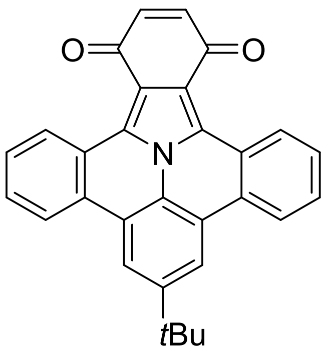

8-(*tert*-butyl)tribenzo[a,d,k]ullazine-1,4-dione (**18a**)

Crude product of **18a** was recrystallized from ethanol.
^{1}H NMR (300 MHz, Methylene Chloride-d2) δ 10.02 (d, *J* = 7.1 Hz, 2H), 8.35 (s, 2H), 8.31 (d, *J* = 7.5 Hz, 2H), 7.65 (t, *J* = 7.5 Hz, 2H), 7.57 (t, *J* = 7.7 Hz, 2H), 6.72 (s, 2H), 1.50 (s, 9H). ^{13}C NMR (176 MHz, C_2D2Cl_4) δ 182.67, 149.49, 140.18, 130.80, 130.27, 129.03, 128.82, 128.32, 126.30, 124.63, 123.30, 122.65, 119.18, 117.32, 35.67, 31.93.

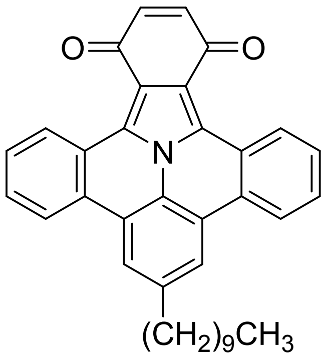

8-(*n*-decyl)tribenzo[a,d,k]ullazine-1,4-dione (**18b**)

Crude product of **18b** was recrystallized from ethanol.
^{1}H NMR (300 MHz, CD_2Cl_2) δ 9.85 (d, *J* = 8.0 Hz, 2H), 8.03 (d, *J* = 7.7 Hz, 2H), 7.82 (s, 2H), 7.59 – 7.42 (m, 4H), 6.64 (s, 2H), 2.80 – 2.67 (m, 3H), 1.78 – 1.65 (m, 2H), 1.46 – 1.23 (m, 29H), 0.95 – 0.83 (m, 3H); ^{13}C NMR (176 MHz, C_2D2Cl_4) δ 182.44, 141.24, 140.00, 130.59, 129.98, 128.87, 128.67, 127.82, 126.10, 124.32, 123.32, 122.53, 121.78, 117.12,

99.78, 36.73, 32.22, 32.06, 29.97, 29.88, 29.80, 29.67, 23.05, 14.57; Elemental analysis for Chemical Formula: $C_{36}H_{33}NO_2$: C, 84.51; H, 6.50; N, 2.74; O, 6.25; Found: C, 83.50; H, 6.20; N, 2.89; HRMS (ESI, *m/z*): calcd for $C_{36}H_{34}NO_2$ $[M+H]^+$ 512.2590, found 512.2612; Due to the low solubility of **18b** in acetonitrile, THF and TFA had to be added for HRMS.

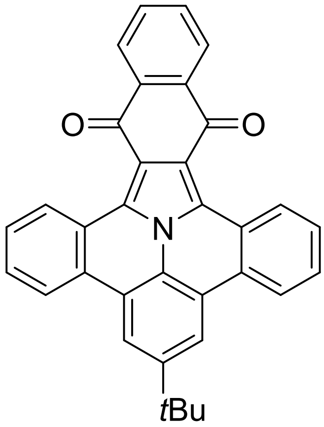

8-(*tert*-butyl)dibenzo[d,k]naphtha[a]ullazine-1,6-dion (**19a**)

Crude product of **19a** was recrystallized from ethanol.
^{1}H NMR (300 MHz, $C_2D_2Cl_4$) δ 10.17 – 10.12 (m, 2H), 8.42 (s, 2H), 8.40 – 8.33 (m, 2H), 8.38 – 8.35 (m, 2H), 7.80 – 7.59 (m, 6H), 1.52 (s, 9H); ^{13}C NMR (75 MHz, $C_2D_2Cl_4$) δ 180.73, 149.24, 135.74, 133.24, 130.27, 129.91, 129.04, 128.73, 128.07, 127.21, 124.70, 123.33, 122.40, 118.89, 118.61, 74.57, 74.46, 74.20, 73.92, 73.83, 35.64, 31.93. Elemental analysis for chemical formula $C_{34}H_{23}NO_2$: C, 85.51; H, 4.85; N, 2.93; O, 6.70; Found: C, 85.08; H, 4.84; N, 2.95; HRMS (ESI, *m/z*): calcd for $C_{34}H_{24}NO_2$ $[M+H]^+$ 478.1807, found 478.1816. Due to the low solubility of **19a** in acetonitrile, chloroform and TFA had to be added for HRMS.

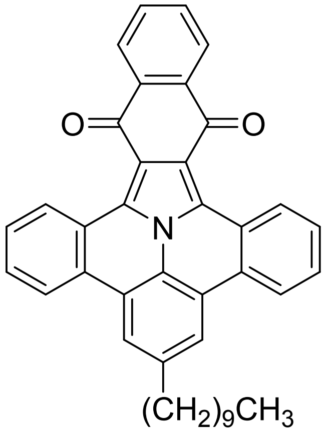

8-(*n*-decyl)dibenzo[d,k]naphtha[a]ullazine-1,6-dion (**19b**)

Crude product of **19b** was recrystallized from ethanol.
^{1}H NMR (700 MHz,) δ 10.11 – 10.05 (m, 2H), 8.26 (dd, *J* = 5.5, 3.4 Hz, 2H), 8.22 (dd, *J* = 6.5, 2.4 Hz, 2H), 8.04 (s, 2H), 7.69 (dd, *J* = 5.8, 3.1 Hz, 2H), 7.60 – 7.53 (m, 4H), 2.80 (t, *J* = 8.0 Hz, 2H), 1.73 (p, *J* = 7.7 Hz, 2H), 1.39 (p, *J* = 7.4 Hz, 2H), 1.34 (p, *J* = 6.7 Hz, 2H), 1.30 – 1.16 (m, 10H), 0.82 (t, *J* = 6.9 Hz, 3H); ^{13}C NMR (176 MHz, C_2D2Cl_4) δ 180.86, 141.29, 135.96, 133.22, 130.21, 129.95, 129.09, 128.78, 127.89, 127.27, 126.46, 124.79, 123.73, 122.51, 121.70, 120.62, 118.76, 36.71, 32.17, 31.91, 29.90, 29.81, 29.72, 29.59, 22.96, 14.44; HRMS (ESI, *m/z*): calcd for $C_{40}H_{36}NO_2$ [M+H]$^+$ 562.2746, found 562.2748. Due to the low solubility of **19b** in acetonitrile, THF and TFA had to be added for HRMS.

One-pot procedure exemplified on the synthesis of N-PAHs 19a

$$\textbf{20a} + \textbf{14} \xrightarrow[\text{toluene, 77\%}]{\text{1) TEA, 60 min; 2) DDQ, 60 min}} \textbf{19a}$$

Figure S5. Synthesis of N-PAH **19a** by cycloaddition and oxidation in one-pot process.

In a dry and inert 25 mL Schlenk tube precursor **20a** (0.05 g, 0.12 mmol, 1.00 eq.) and the dipolarophile **14** (0,02 g, 0,12 mmol, 1.00 eq.) were suspended in anhydrous toluene (4 mL). Under vigorous stirring anhydrous triethylamine (80 µL, 4.80 eq) was added and stirring was continued for 60 minutes. DDQ (83 mg, 0.37 mmol, 3.00 eq.) was added to the flask. The reaction was quenched after 60 minutes by the addition of water (10 mL). The crude product was filtered and washed with water and recrystallized from ethanol to afford the title compound (0.05 g, 0.10 mmol, 77% yield).

General procedure for the twofold-cycloaddition-planarization sequence for N-PAHs 21a – 21b

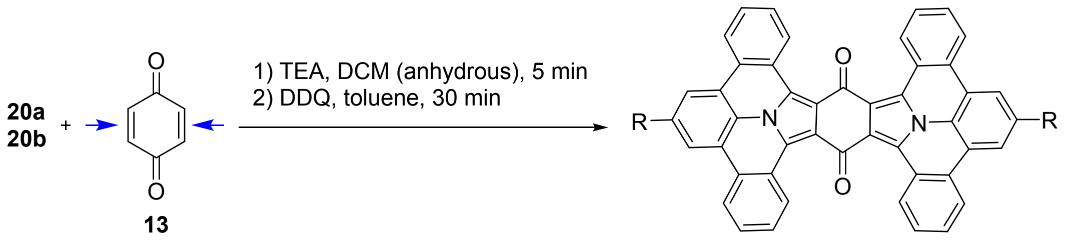

Figure S6 General reaction scheme for the twofold-cycloaddtion-planariazation synthesis of N-PAHs **21a** – **21b**

In a dry and inert 25 mL Schlenk tube the precursor **20a** – **20b** (0.24 mmol, 2.00 eq.) and dipolarophile **13** (0.013 g, 0.12 mmol, 1.00 eq.) were dissolved in anhydrous dichloromethane (5 mL). Under vigorous stirring triethylamine (anhydrous, 0.25 mL, ~12.00 eq) was added in one shot. The reaction was stirred for several minutes and transferred to a round bottom flask afterwards. Solvents and residual triethyl amine were removed under reduced pressure to obtain the crude product. DDQ was added and the flask was sealed with a septum and evacuated and refilled with argon for three times. Anhydrous toluene (4 mL) was added via a syringe under argon and stirring was continued for 30 min. The reaction was quenched by addition of water (10 mL). The precipitate was filtered and purified by recrystallization from ethanol to afford the title compound **21a** – **21b** as red solids.

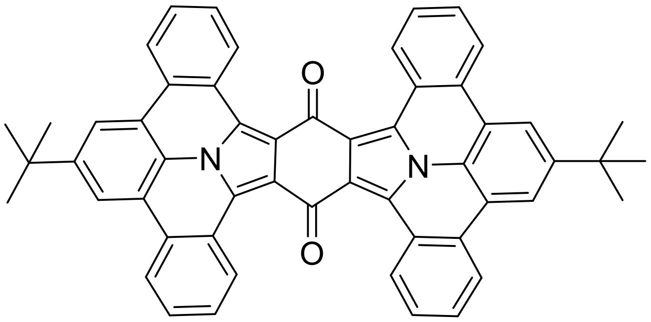

benzo[1,2-a:4,5-a']-bis(8-(*tert*-butyl)-dibenzo[d,k]ullazine) (**21a**)

Crude product of **21a** was recrystallized from ethanol.

^{1}H NMR (500 MHz, 403 K) δ 10.26 (s, 4H), 8.48 (br, 4H), 8.40 (br, 4H), 7.77 – 7.63 (br, 8H), 1.62 (s, 18H); ^{13}C NMR (126 MHz, C_2D2Cl_4, 403 K) δ 180.21, 129.32, 129.13, 128.38, 128.07, 125.67, 123.64, 123.62, 122.21, 122.18, 120.44, 118.43, 118.40, 35.39, 31.69 (The observed peaks in NMR expermiments were significantly broadened due to the low solubility of **23a** even at 403 K); Elemental analysis for Chemical Formula: $C_{54}H_{38}N_2O_2$: C, 86.84; H, 5.13; N, 3.75; O, 4.28; Found: C, 86.17; H, 5.16; N, 3.84; HRMS (ESI, *m/z*): calcd for $C_{54}H_{39}N_2O_2$ $[M+H]^+$ 747.3029, found 747.3012. Due to the low solubility of **21a** in acetonitrile, THF and TFA had to be added for HRMS.

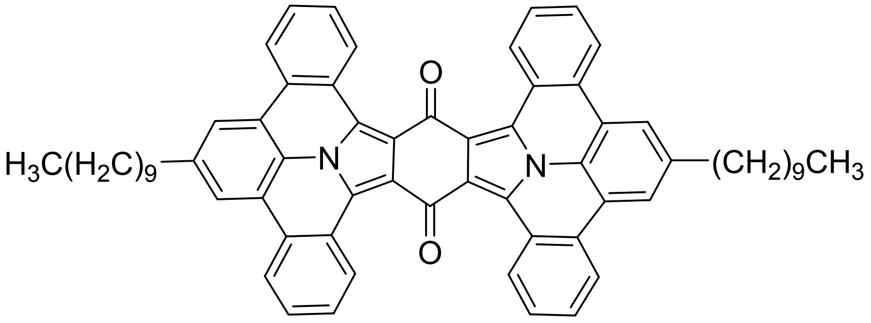

benzo[1,2-a:4,5-a’]-bis(8-(*n*-decyl)-dibenzo[d,k]ullazine) (**21b**)

Crude product of **21b** was recrystallized from ethanol.
^{1}H NMR (500 MHz, $C_2D_2Cl_4$, 403 K) δ 10.27 (d, *J* = 8.3 Hz, 4H), 8.39 (d, *J* = 7.9 Hz, 4H), 8.23 (s, 4H), 7.74 (t, *J* = 7.6 Hz, 4H), 7.66 (t, *J* = 7.5 Hz, 4H), 2.99 (s, 4H), 1.90 (p, *J* = 7.4, 6.6 Hz, 4H), 1.38 – 1.19 (m, 38H), 0.89 (t, *J* = 6.8 Hz, 9H); ^{13}C NMR (126 MHz, $C_2D_2Cl_4$) δ 180.16, 149.34, 140.55, 129.17, 129.10, 128.41, 128.18, 127.74, 126.73, 125.52, 123.91, 122.28, 122.07, 121.25, 111.25, 101.68, 36.51, 31.81, 31.46, 29.55, 29.52, 29.47, 29.40, 29.19, 22.51, 13.82; Elemental analysis for chemical formula $C_{66}H_{62}N_2O_2$: C, 86.61; H, 6.83; N, 3.06; O, 3.50; Found: C, 85.39; H, 7.38; N, 3.03; HRMS (ESI, *m/z*): calcd for $C_{66}H_{63}N_2O_2$ $[M+H]^+$ 915.4890, found 915.4902.

2) X-ray crystallographic analysis

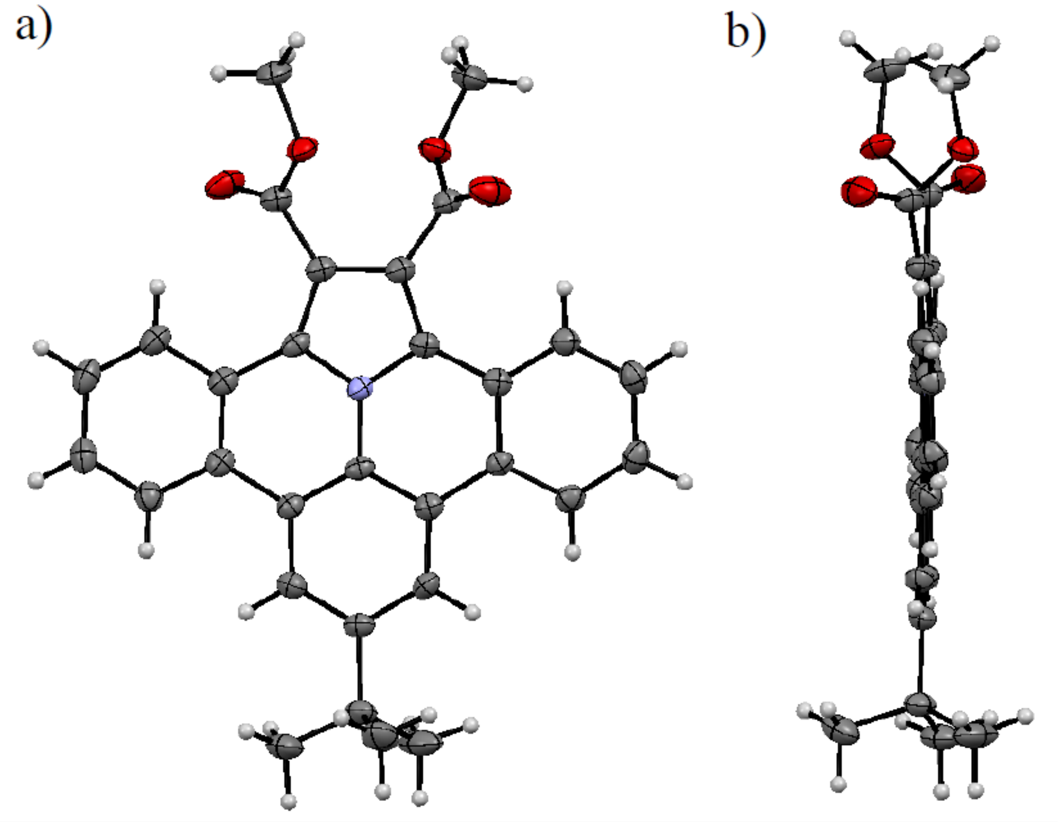

Figure S7. X-ray crystal structure of **5**. ORTEP drawing (a: top view, b: side view). Oxygen atoms are labled in red and the nitrogen atom in blue.

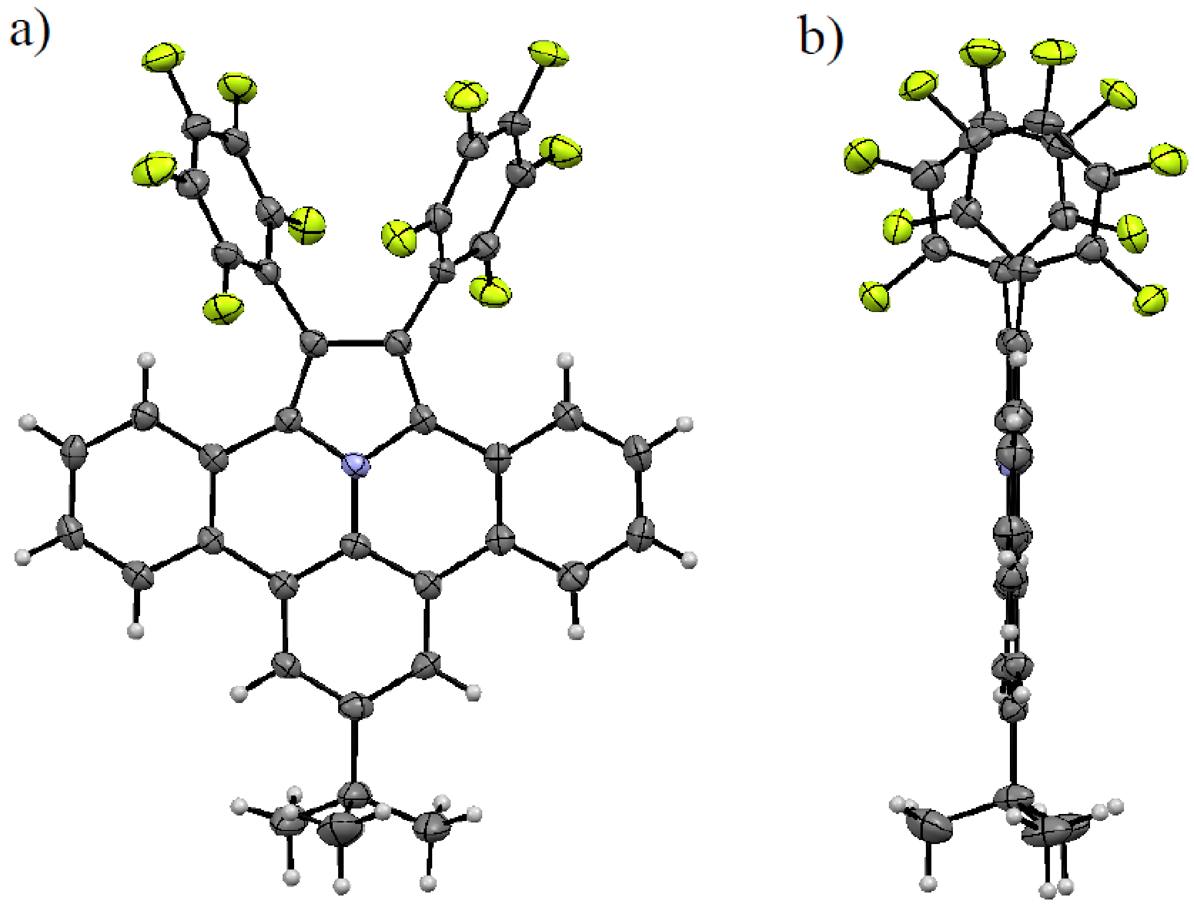

Figure S8. X-ray crystal structure of **16**. ORTEP drawing (a: top view, b: side view). Fluorine atoms are labled in yellow and the nitrogen atom in blue.

Details of the crystal data and a summary of the intensity data collection parameters for **15a** and **16** are listed in Table S1 and deposited at the Cambridge Crystallographic Data Centre. In each case, suitable crystals were measured with a Bruker APEX II diffractometer. Graphite-monochromated Mo Kα radiation was used. The structures were solved by direct methods with SIR-97 and refined by the full-matrix least-squares techniques against F2 (SHELXL-97).[1] The intensities were corrected for Lorentz and polarization effects. The non-hydrogen atoms were refined anisotropically. Hydrogen atoms were refined using the riding model.[2, 3] The crystals structures were visualized using Mercury 3.3.

Table S1: Crystallographic data and structure refinement details of **15a** and **16**. Single crystals of **15a** and **16** were obtained from dichlromethane.

compound	5	**16**
CCD Deposition Number	CCDC 1022817	CCDC 1022816
Molecular Formula	$C_{30}H_{25}NO_4$	$2(C_{38}H_{19}F_{10}N)$, CH_2Cl_2
Formula weight	463.53	1444.01
Crystal dimensions	0.32 x 0.54 x 0.57 mm^3	0.06x0.28x0.31 mm^3
Crystal color	colorless	colorless
Crystal system	triclinic	triclinic
Space group	P -1	P -1

a	12.1231(10) Å	12.6166(15) Å
b	12.9429(11) Å	16.350(2) Å
c	17.2915(14) Å	16.738(2) Å
α	95.734(2)°	87.227(2)°
β	108.021(2)°	75.478(3)°
γ	113.1440(19)°	67.501(3)°
Cell volume	2295.0(3)$Å^3$	3083.5()$Å^3$
Z value	4	2
μ	0.09 mm^{-1}	0.216 mm^{-1}
D_{calc}	1.341 gcm^{-3}	1.555 gcm^{-3}
F000	976	14600
Temperature	173 K	173 K
Method of determination of unit cell dimensions	Calculated from 6391 reflections with 2.2° ≤ θ ≤ 27.8°	Calculated from 79031 reflections with 2.4° ≤ θ ≤ 26.7°
Number of reflections measured	22977	50350
Unique number of reflections	10930	14688
R_{int}	0.0268	0.037
Theta range	2° ≤ θ ≤ 28°	2° ≤ θ ≤ 28°
Index range	-15≤h≤15; -17≤k≤16; -22≤l≤22	-15≤h≤16; -21≤k≤21; 0≤l≤22
Residuals: R_1 (I > 2.00σ(I)	0.0464	0.0485
Residuals: R_1 (all reflections)	0.0698	0.0809
wR_2	0.1258	0.1135
Number of parameters refined	671	935
Goodness of fit (*S*)	1.006	1.013
Max shift/error	0.001 * esd	0.001 * esd
Remarks	Structure contains two independent molecules and one is disordered.	Structure contains two independent molecules A and B and one solvent molecule (CH_2Cl_2), which is disordered. The crystal is twinned.

3) Calculations

DFT calculations were performed on **Gaussian09** simulation package[4] using B3LYP functional with **6-31g (d,p)** basis set.[5] For compound **5** and **16** the geometric parameters obtained by the crystal structure was used for energy calculations. The geometries of **17a**, **18a**, **19a** and **23** are based on the crystal structure of **16**, optimized on the AM1 level and computed with DFT B3LYP at the 6-31g(d,p) level. The default algorithm of optimization is the Berny algorithm using GEDIIS in redundant internal coordinates (www.gaussian.com).

Input for optimization:
opt b3lyp/6-31g(d,p)

Input for energy calculation exemplified on parent compound **23**:
b3lyp/6-31g(d,p)

Dibenzoullazine **23**
Symbolic Z-matrix:
Charge = 0 Multiplicity = 1

N	8.216	7.2118	7.1666
C	8.6889	6.7754	5.0455
C	9.1061	6.5285	6.3532
C	10.2303	5.8526	6.9624
C	11.2494	5.2746	6.1891
H	11.1807	5.2872	5.2408
C	12.345	4.6905	6.7815
H	13.0283	4.3094	6.2424
C	12.4567	4.6571	8.1691
H	13.2098	4.2485	8.5772
C	11.4649	5.2221	8.9506
H	11.5487	5.1963	9.8959
C	10.3338	5.8345	8.3788
C	9.3094	6.4892	9.1907
C	9.3306	6.5098	10.5806
H	9.9931	6.0013	11.0333

C	8.4225	7.2414	11.3385
C	7.4173	7.9312	10.6698
H	6.7899	8.4333	11.1757
C	7.3008	7.9108	9.2729
C	6.2132	8.5814	8.5502
C	5.1364	9.1971	9.2289
H	5.1203	9.1978	10.179
C	4.1074	9.7966	8.532
H	3.3884	10.1969	9.006
C	4.1163	9.8192	7.141
H	3.4163	10.252	6.6663
C	5.1433	9.2099	6.4551
H	5.1374	9.2112	5.5045
C	6.2012	8.5856	7.1384
C	7.2643	7.8915	6.4281
C	7.555	7.6385	5.0944
C	8.2758	7.2091	8.5563
H	8.49529	7.27254	12.40557
H	9.14172	6.38447	4.15835
H	7.02147	8.0238	4.25072

Surfaces of highest occupied molecular orbital (HOMO) and lowest unoccupied molecular orbital (LUMO) of compounds **5**, **16**, **17a**, **18a**, **19a** and **21a** all reveal a nodal plane bisecting the molecule along the nitrogen atom. The HOMO of all derivatives relies mainly on the dibenzoullazine core as the donor. The LUMO is strongly influenced by the substituents attached in the 1,2-positions and shifts to the acceptor part of the molecule dependent on accepting strength and conjugation of the electron withdrawing groups. Graphical representation of HOMO and LUMO are given in Figure S9 – 22.

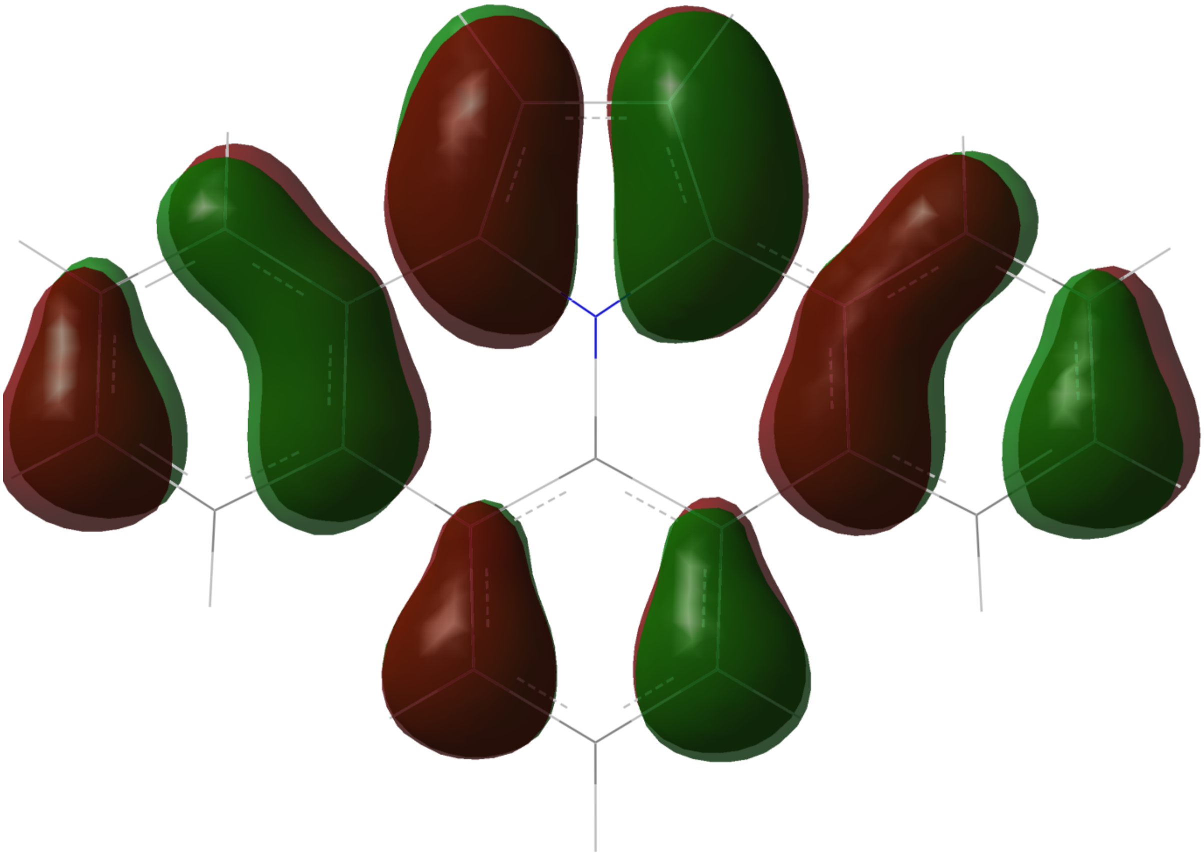

Figure S9. Representation of HOMO of parent dibenzoullazine **23**

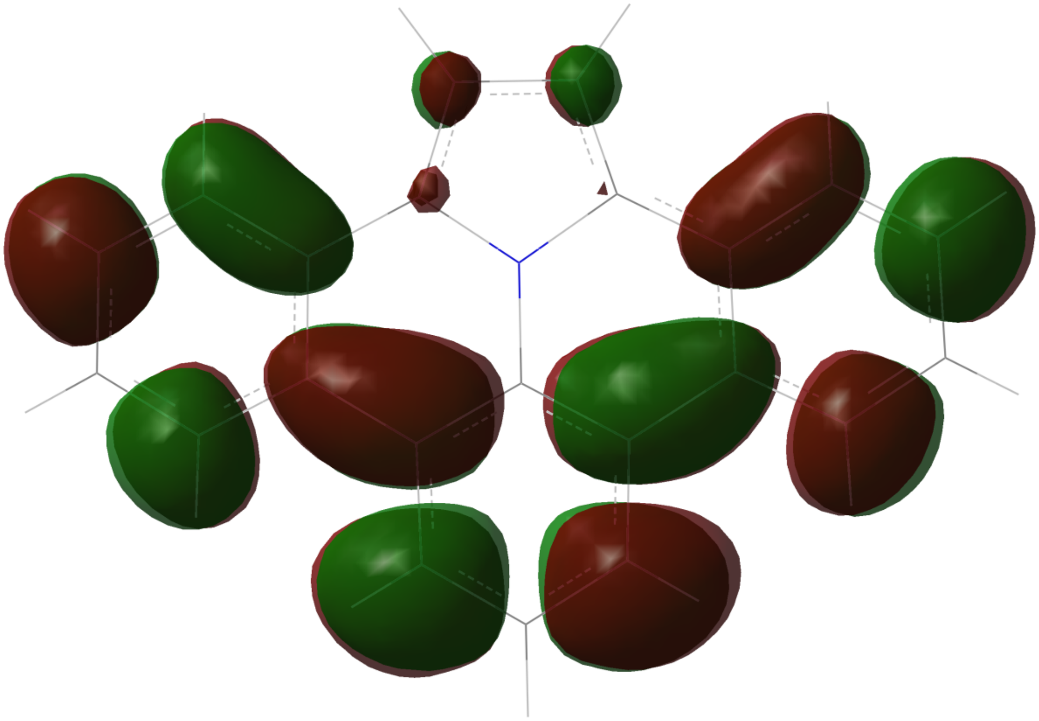

Figure S10. Representation of LUMO of parent dibenzoullazine **23**

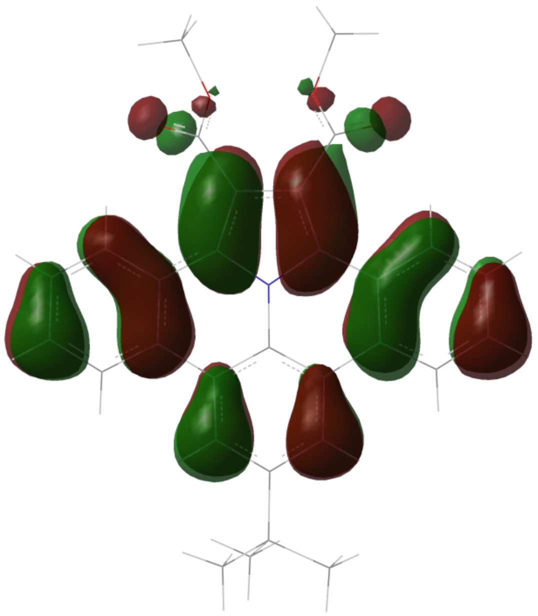

Figure S11. Representation of HOMO of compound **5**

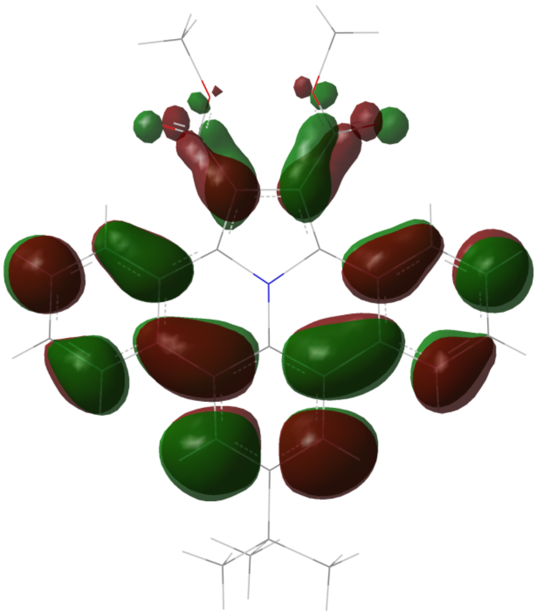

Figure S12. Representation of LUMO of compound **5**

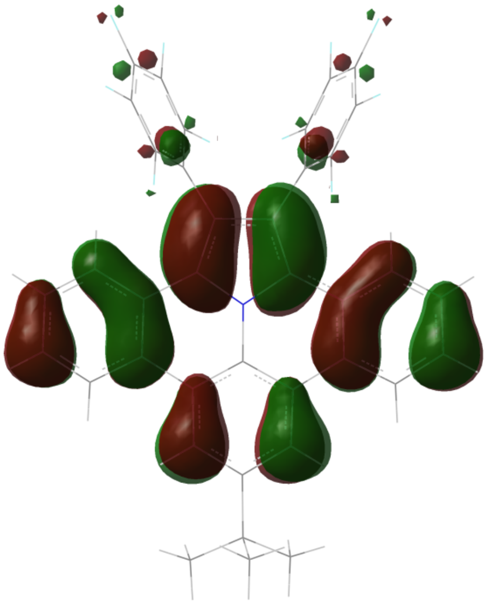

Figure S13. Representation of HOMO of compound **16**

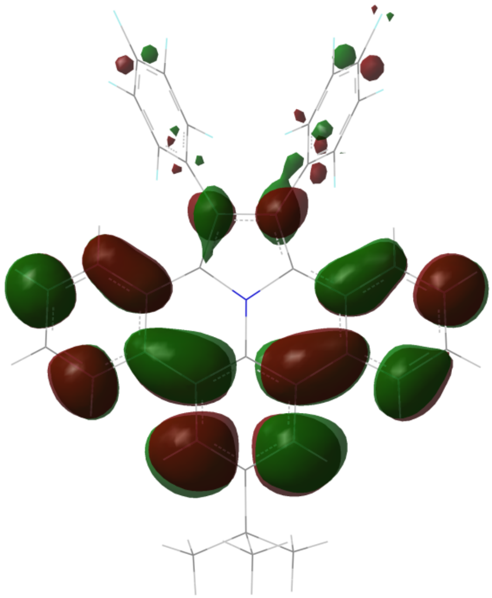

Figure S14. Representation of LUMO of compound **16**

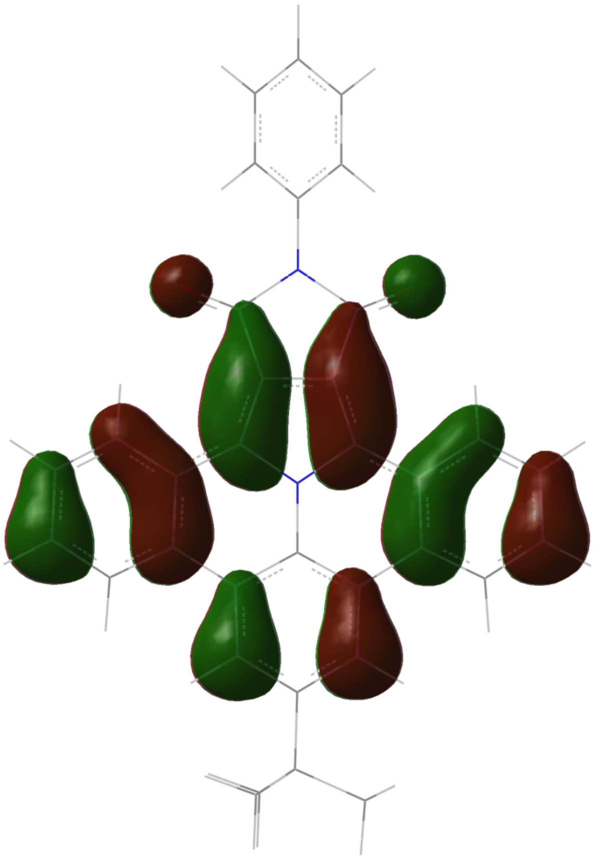

Figure S15. Representation of LUMO of compound **17**

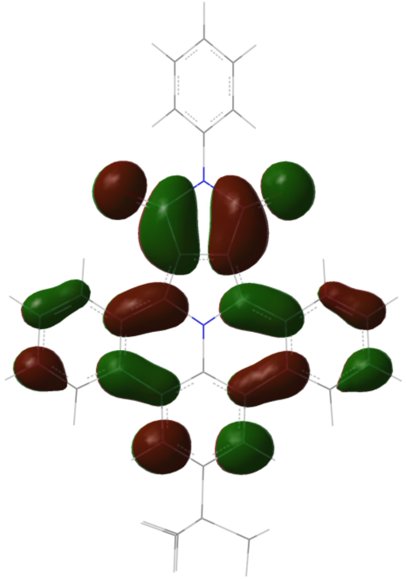

Figure S16. Representation of LUMO of compound **17**

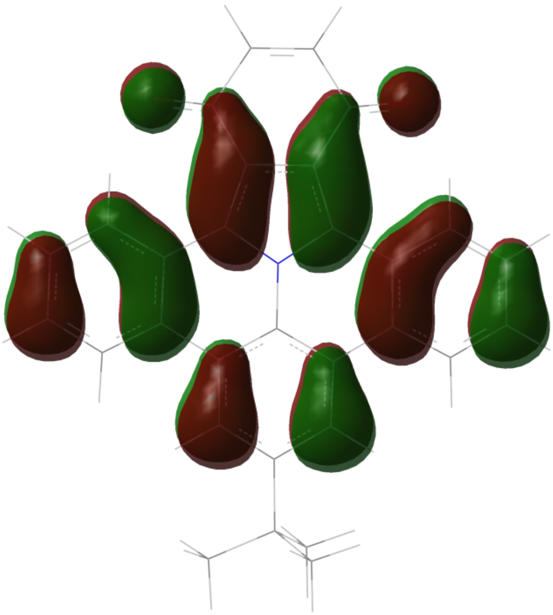

Figure S17. Representation of HOMO of compound **18a**

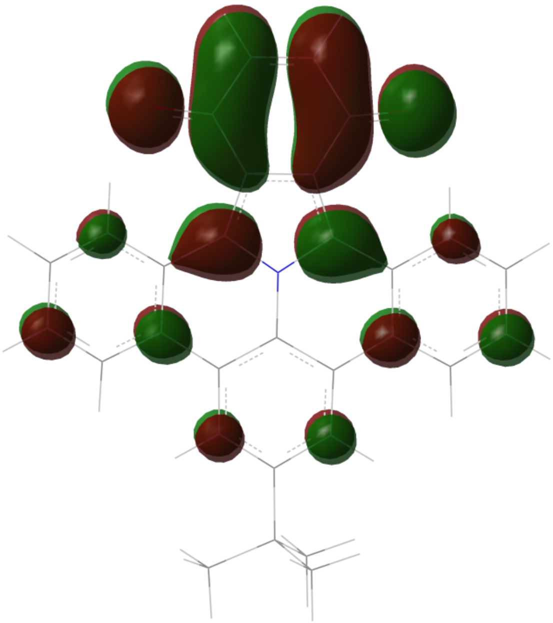

Figure S18. Representation of LUMO of compound **18a**

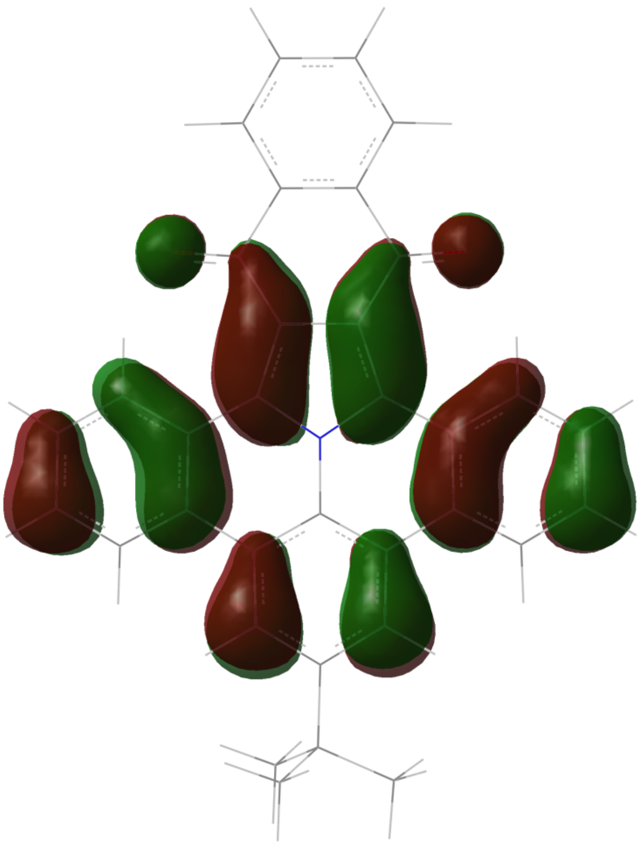

Figure S19. Representation of HOMO of compound **19a**

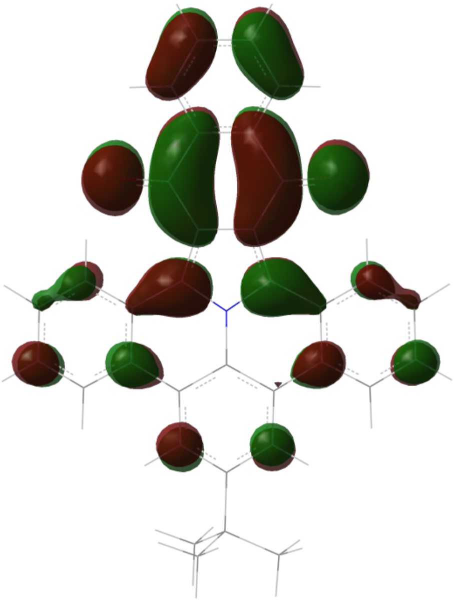

Figure S20. Representation of LUMO of compound **19a**

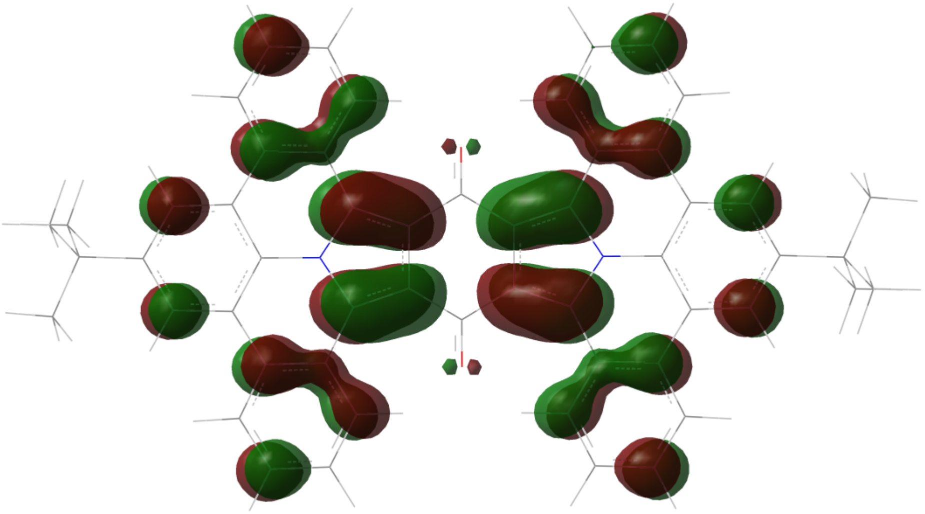

Figure S21. Representation of HOMO of compound **21a**

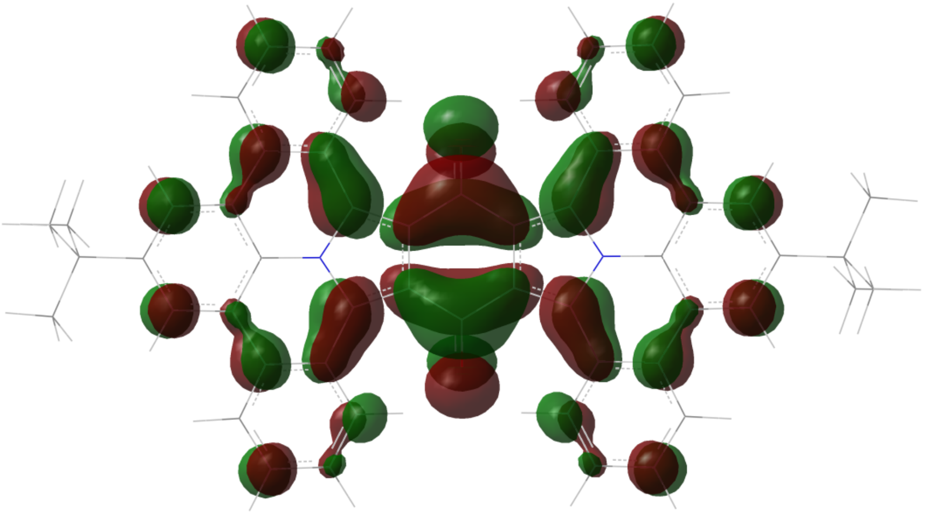

Figure S22. Representation of LUMO of compound **21a**

4) UV-vis absorption spectra

General information

Solution UV-vis absorption spectra were recorded in anhydrous solutions at a concentration of 10^{-5}moll^{-1} in THF.

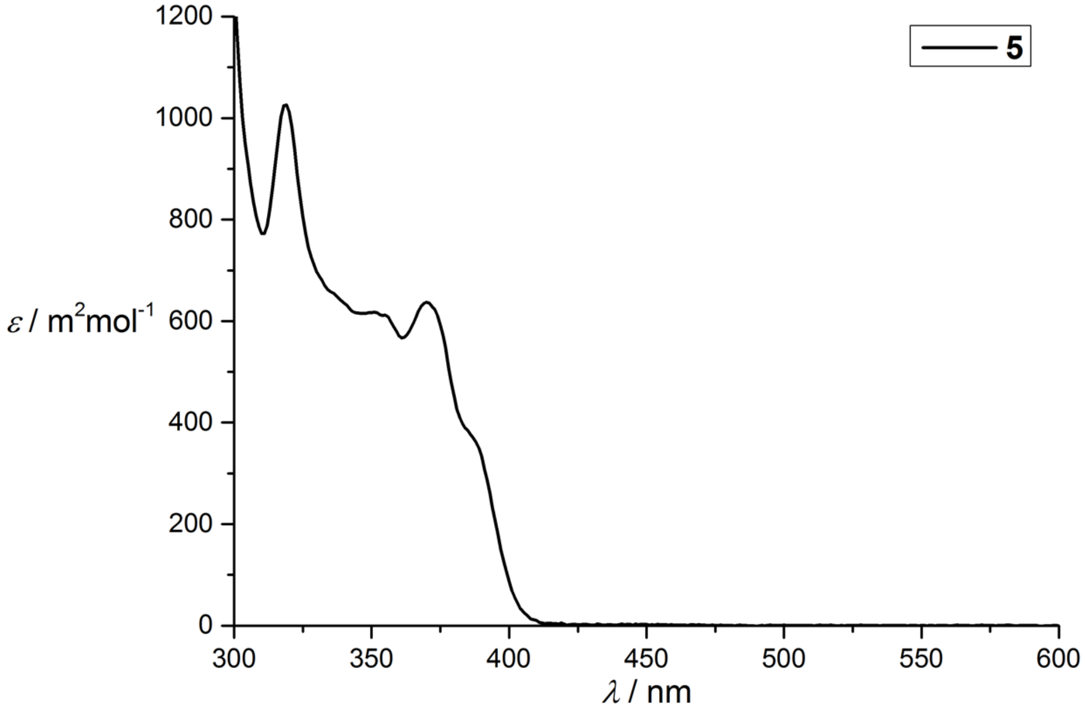

Figure S23. UV-vis absorption spectrum of compound **5** in THF.

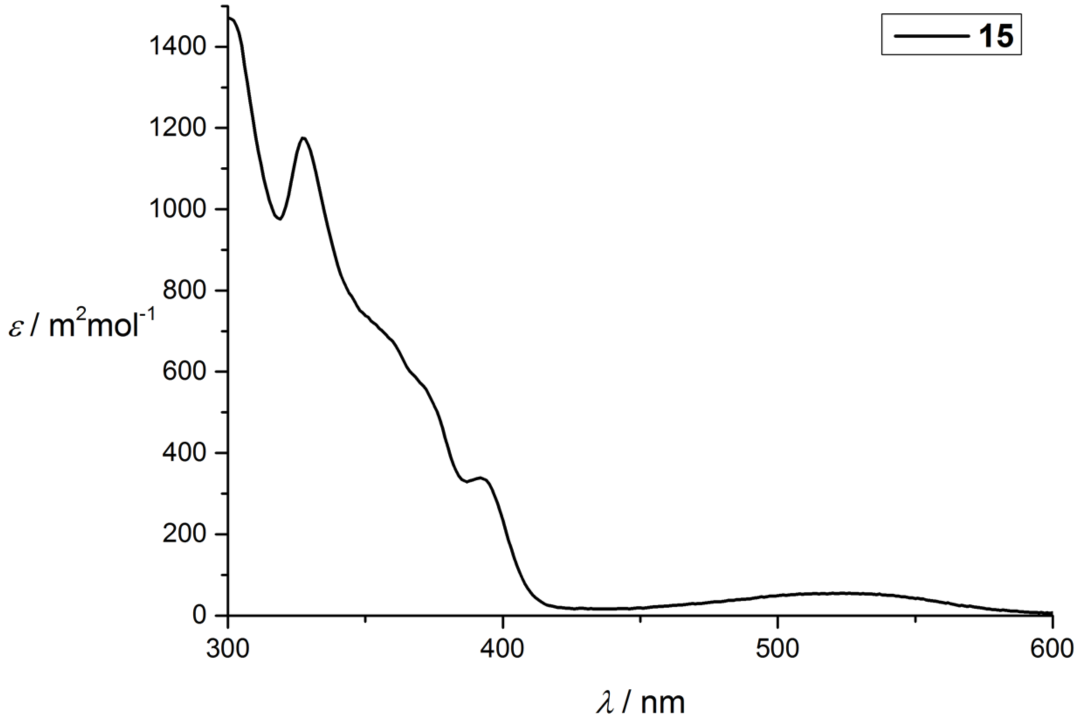

Figure S24. UV-vis absorption spectrum of compound **15** in THF.

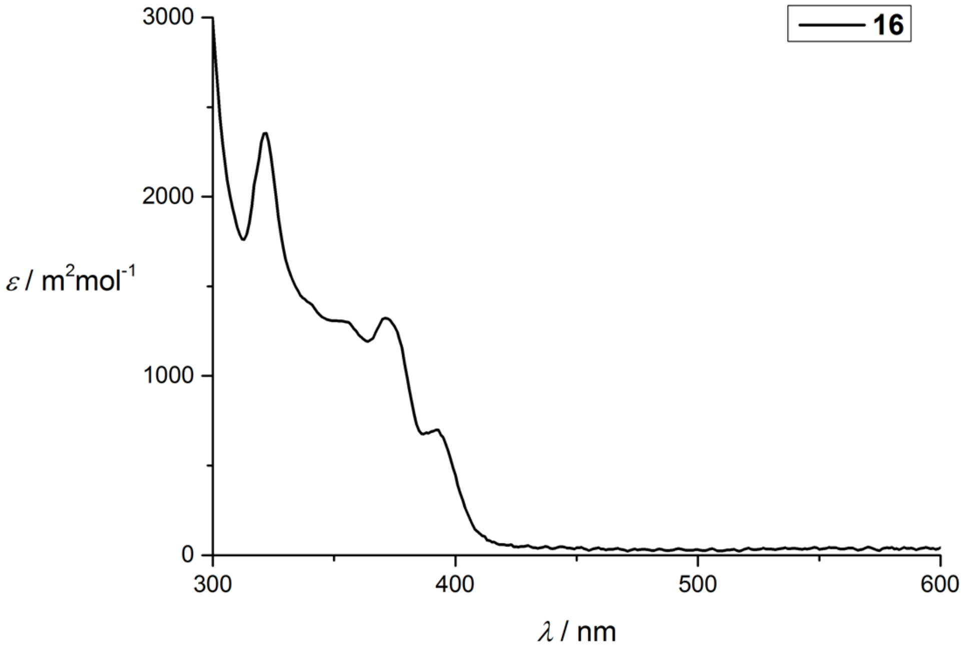

Figure S25. UV-vis absorption spectrum of compound **16** in THF.

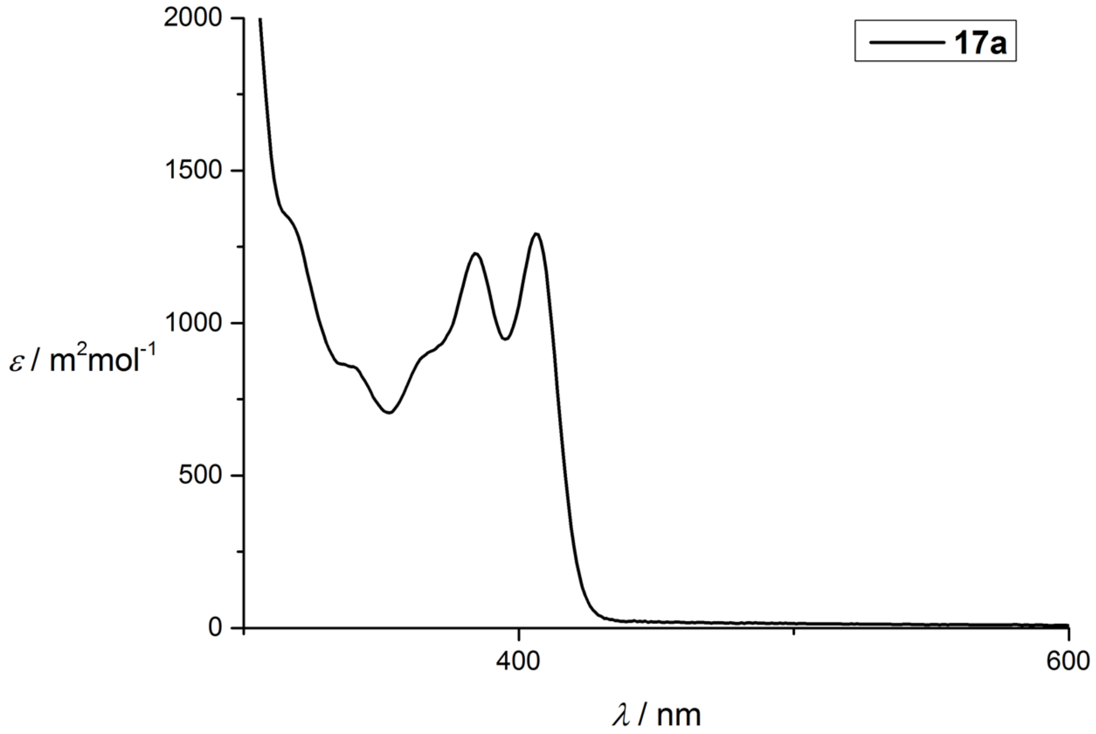

Figure S26. UV-vis absorption spectrum of compound **17a** in THF.

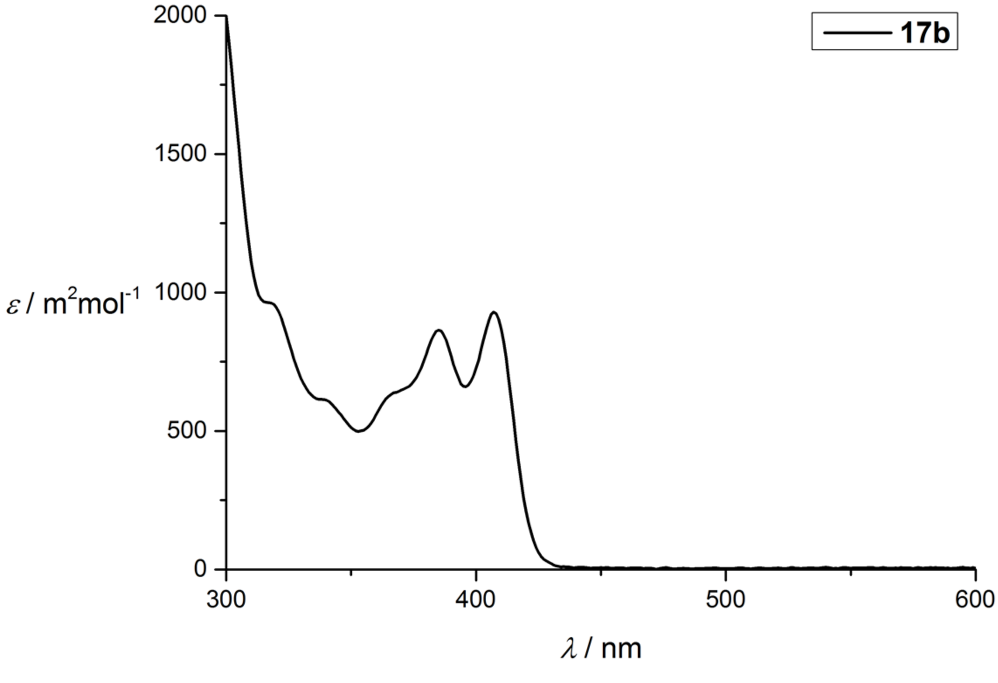

Figure S27. UV-vis absorption spectrum of compound **17b** in THF.

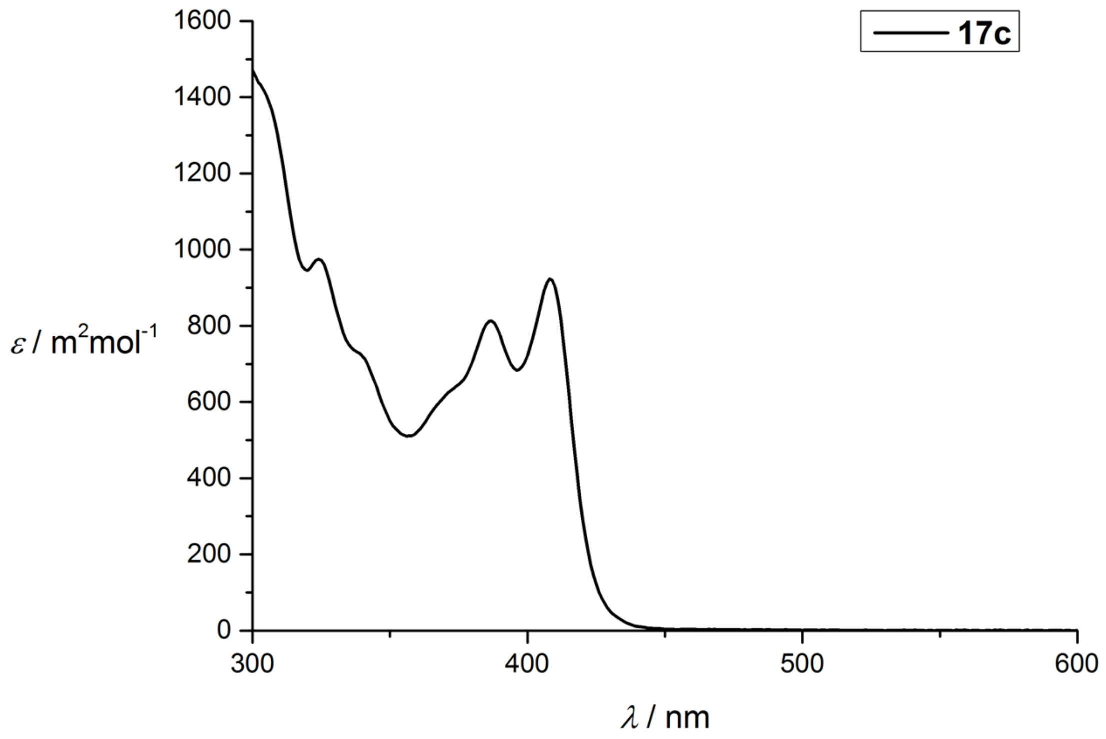

Figure S28. UV-vis absorption spectrum of compound **17c** in THF.

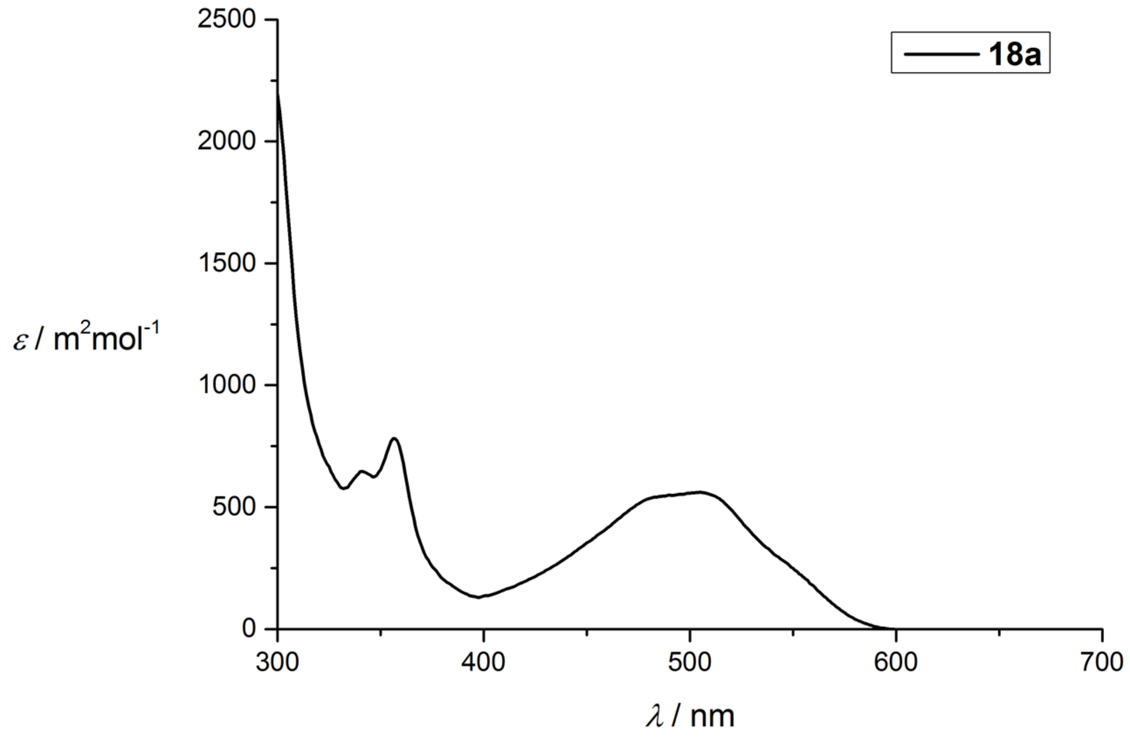

Figure S29. UV-vis absorption spectrum of compound **18a** in THF.

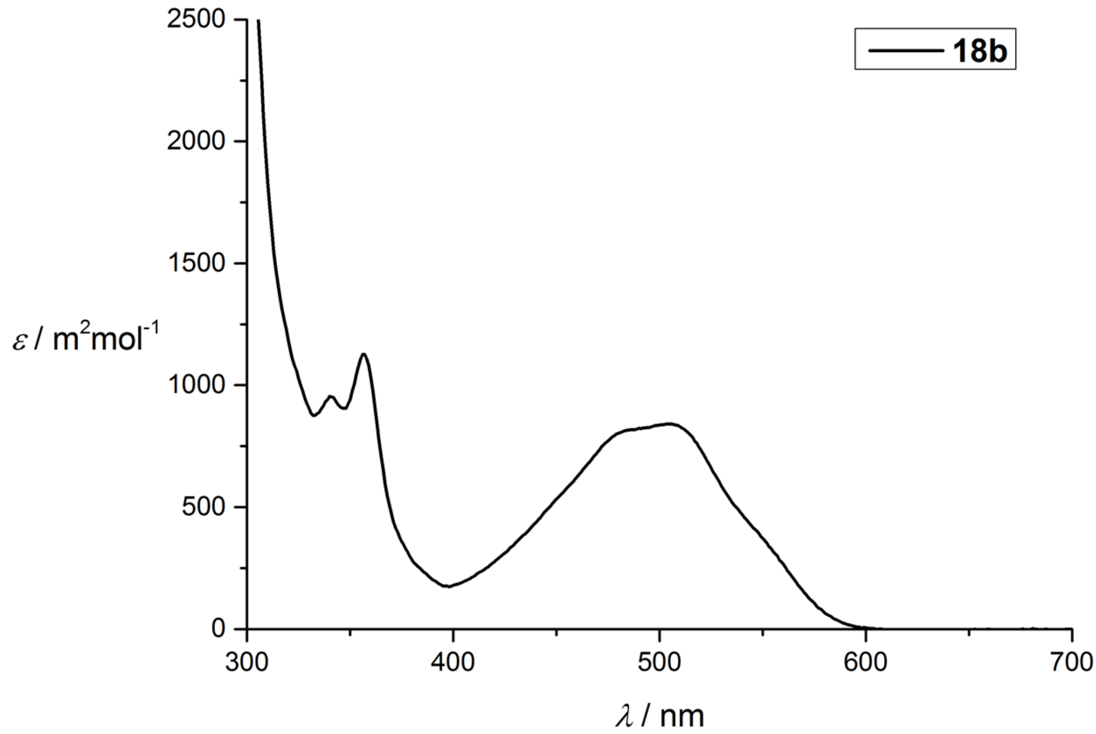

Figure S30. UV-vis absorption spectrum of compound **18b** in THF.

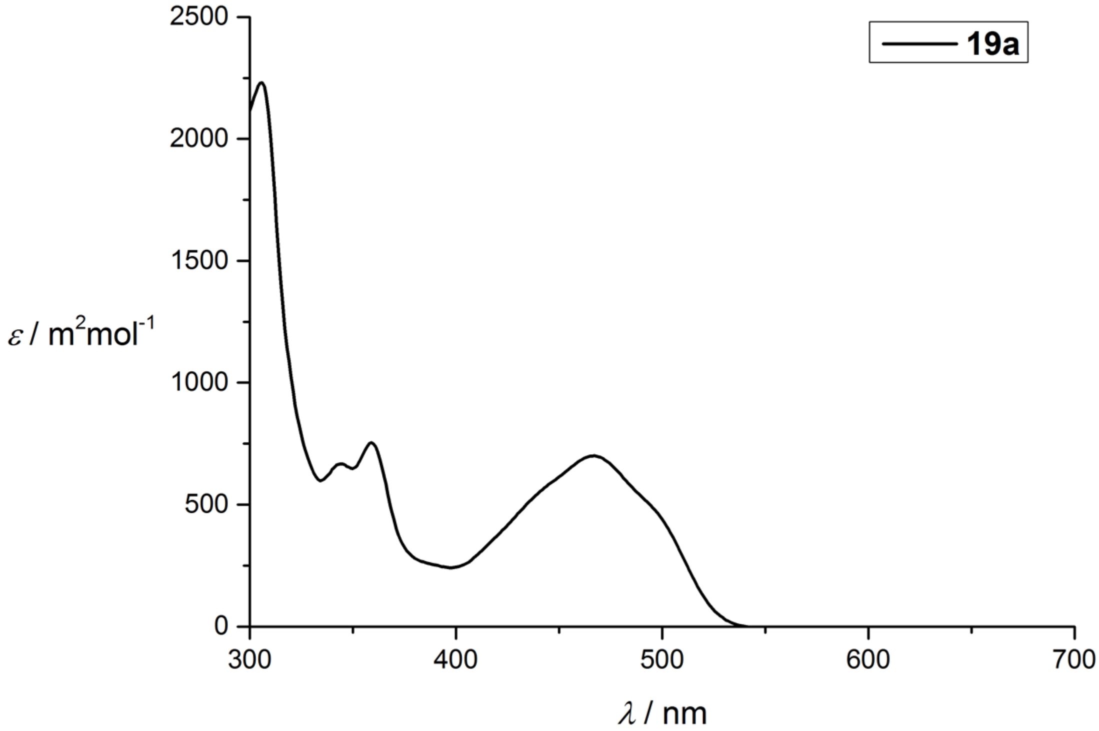

Figure S31. UV-vis absorption spectrum of compound **19a** in THF.

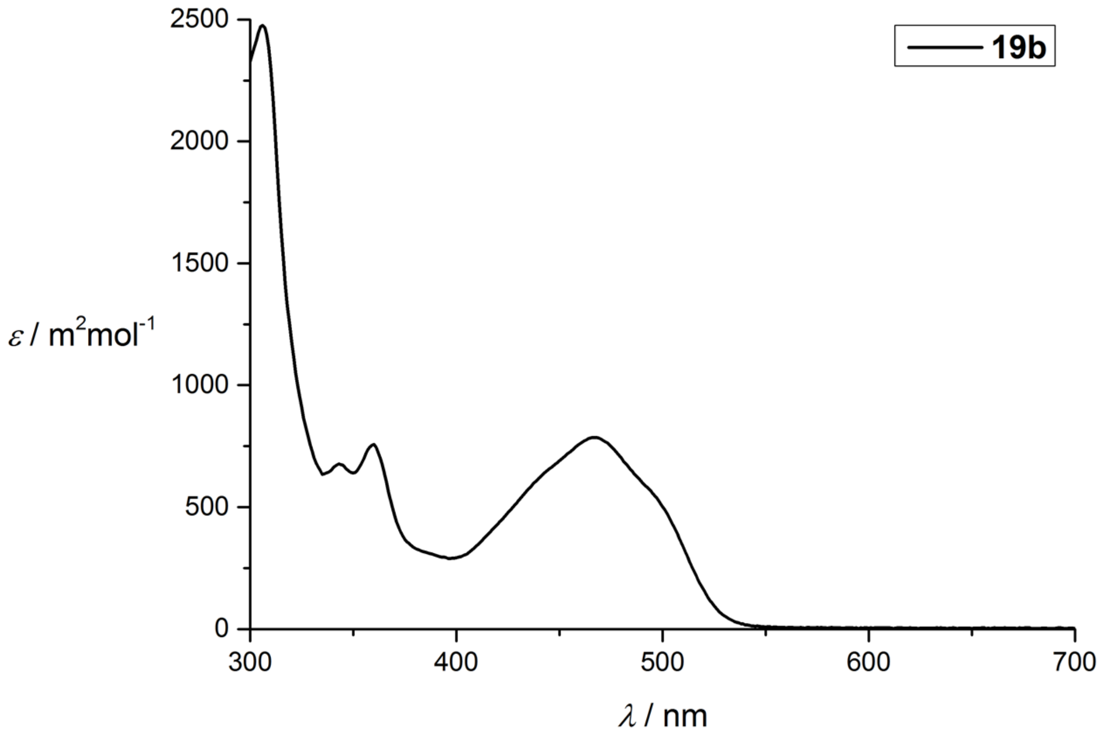

Figure S32. UV-vis absorption spectrum of compound **19b** in THF.

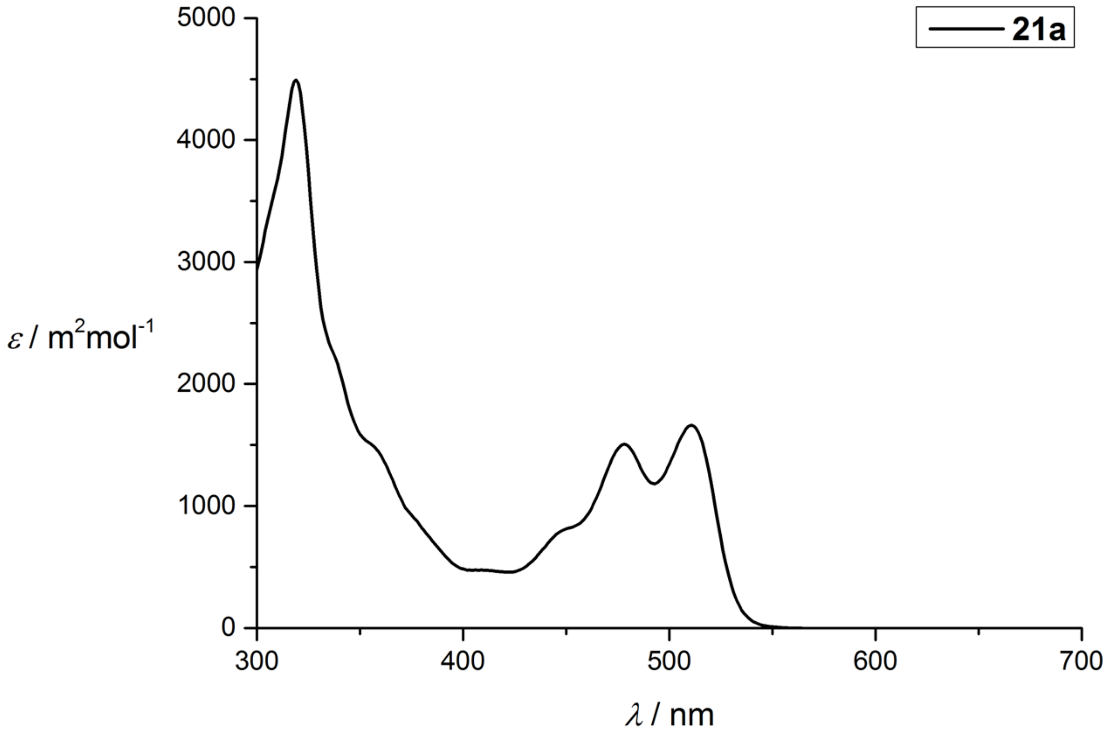

Figure S33. UV-vis absorption spectrum of compound **21a** in THF.

5) Cyclic Voltammetry

Cyclic Voltammetry were recorded from 0.1 M solutions of nBu_4PF_6 and the respective compound (**5**, **15**, **16**, **17a**, **18a**, **19a**, **21a**) in anhydrous acetonitrile at a scan rate of 50 mVs^{-1}.

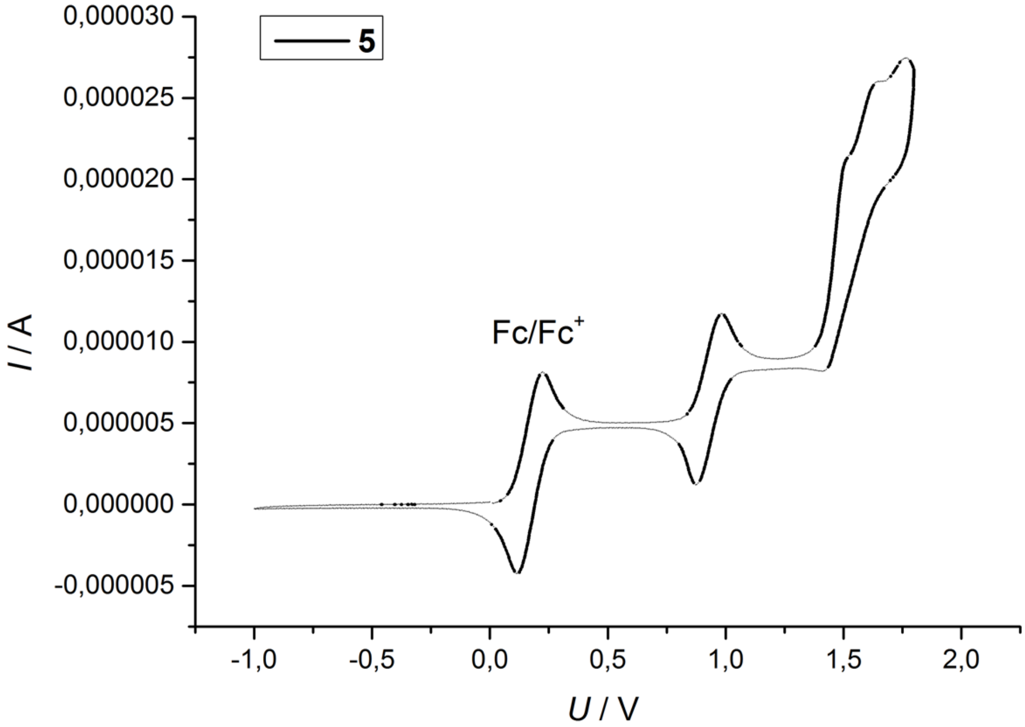

Figure S34. Cyclic voltammograms of compound **5** in acetonitrile.

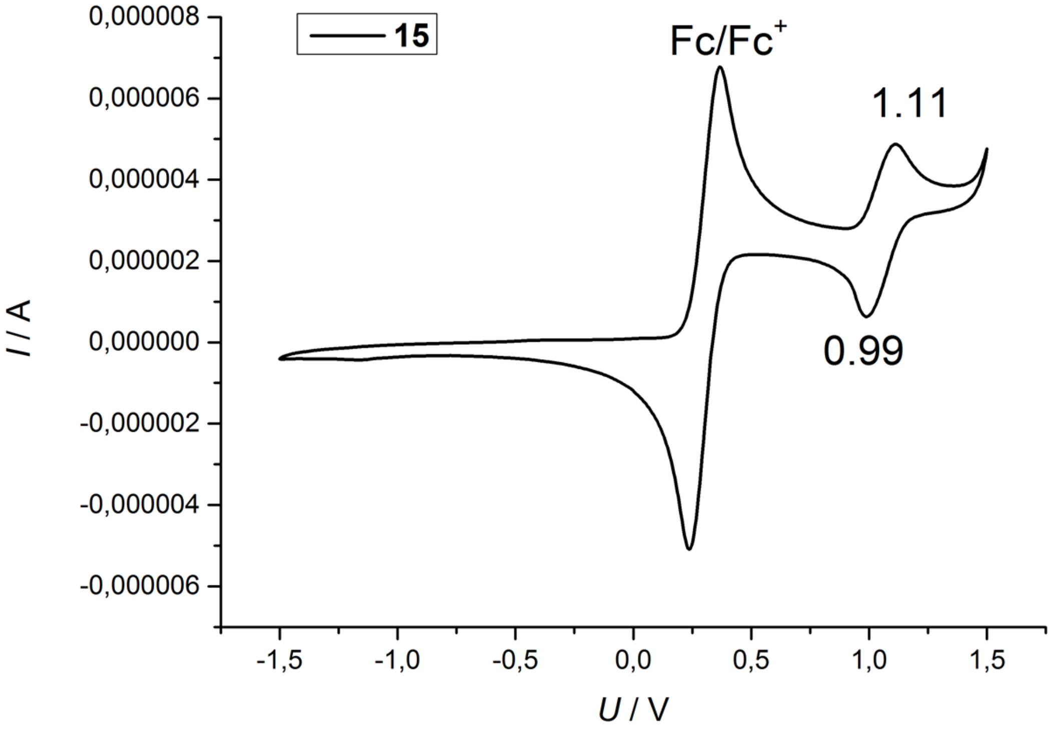

Figure S35. Cyclic voltammograms of compound **15** in acetonitrile.

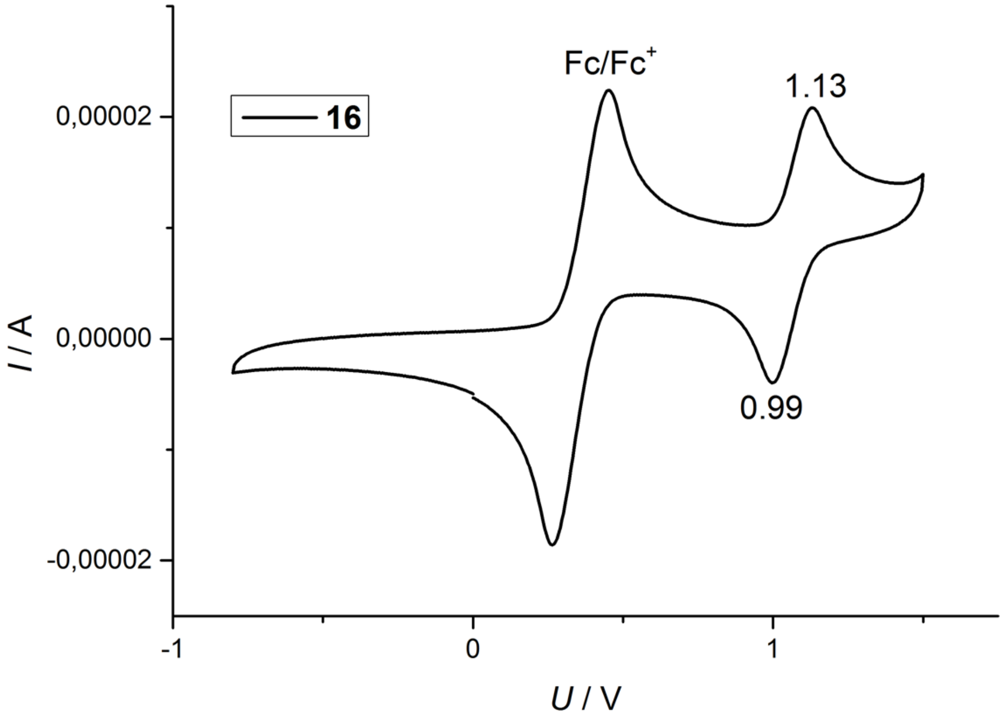

Figure S36. Cyclic voltammograms of compound **16** in acetonitrile.

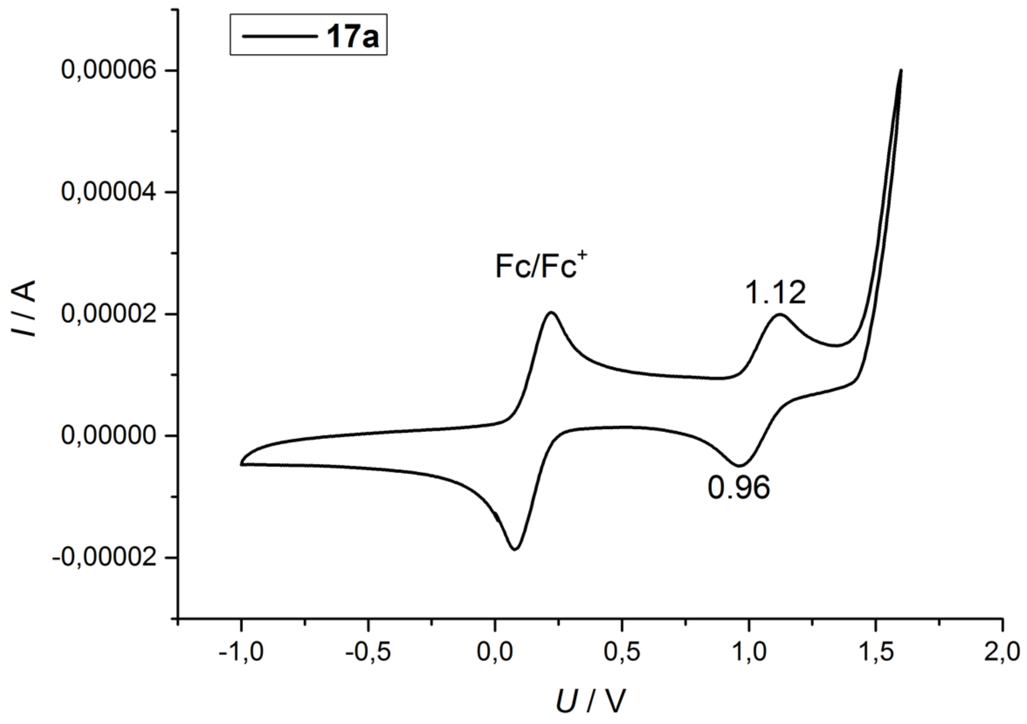

Figure S37. Cyclic voltammograms of compound **17a** in acetonitrile.

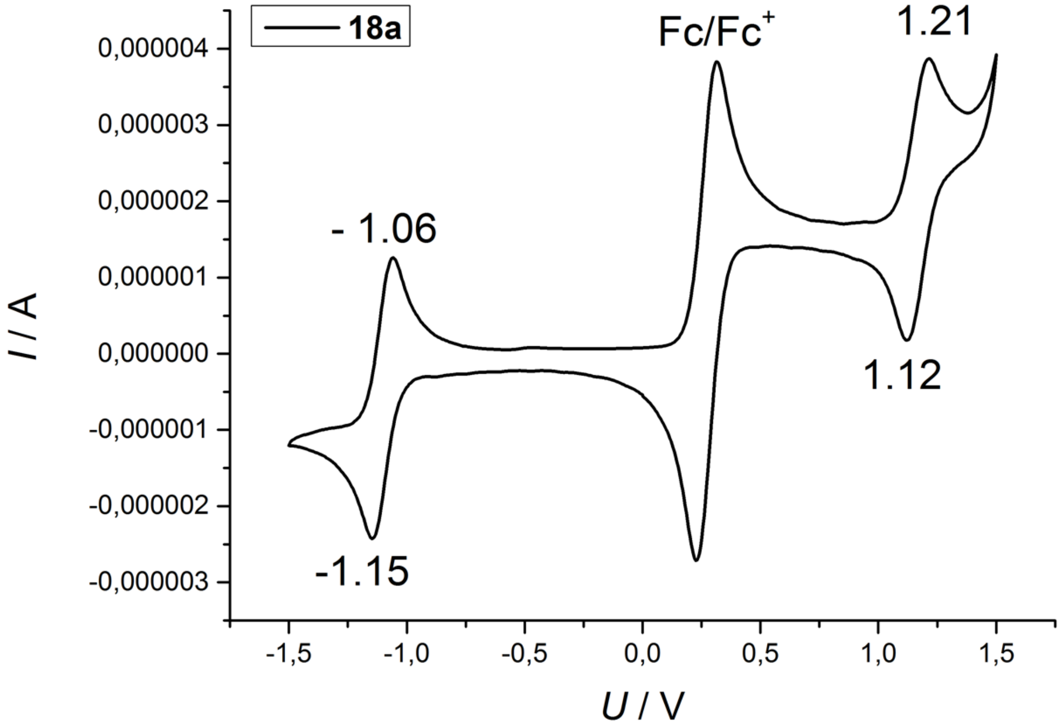

Figure S38. Cyclic voltammograms of compound **18a** in acetonitrile.

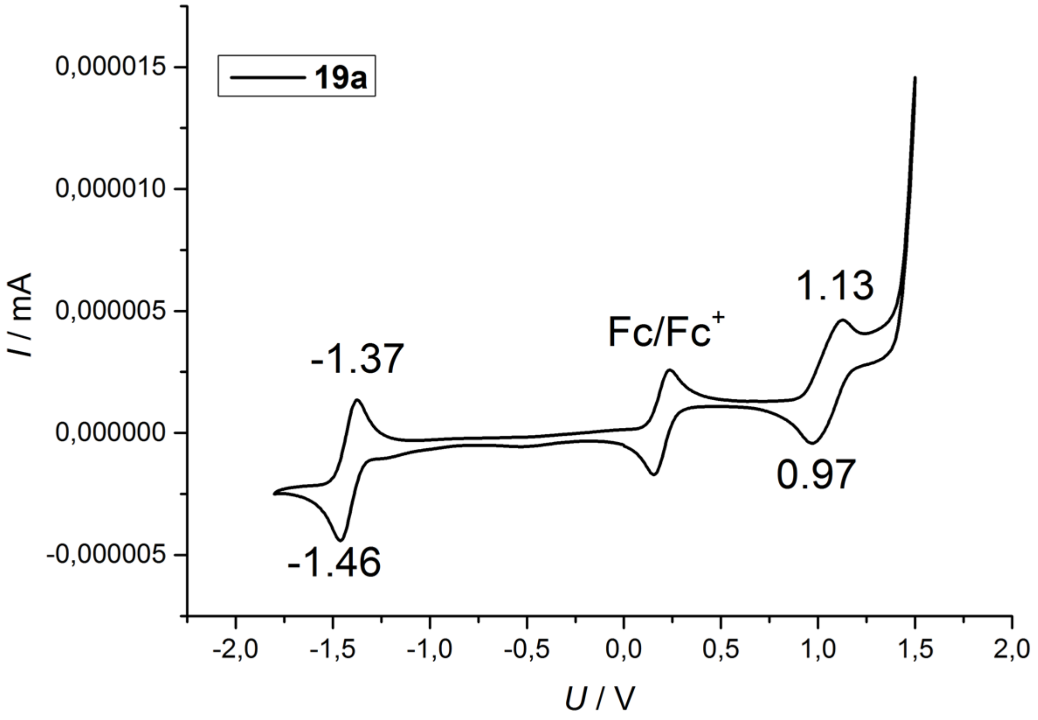

Figure S39. Cyclic voltammograms of compound **19a** in acetonitrile.

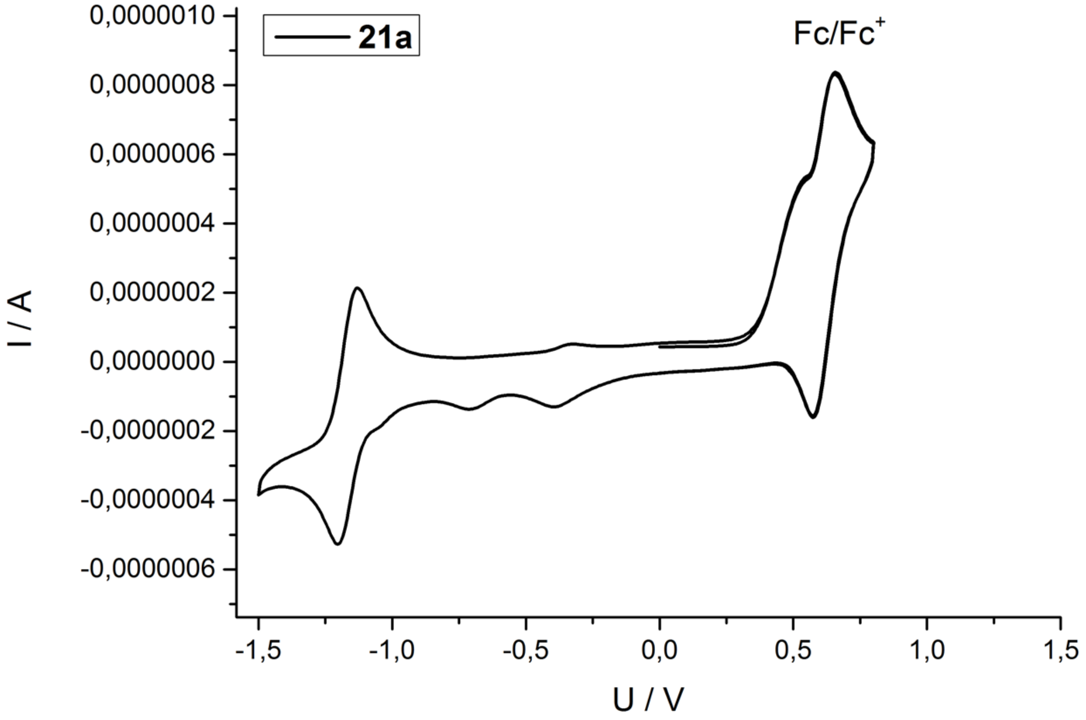

Figure S40. Cyclic voltammograms of compound **21a** in acetonitrile.

6) NMR-spectra

^{1}H-NMR (300 MHz, top) and ^{13}C-NMR (75.5 MHz, bottom) spectra of **25a** at 298 K in THF-d_8.

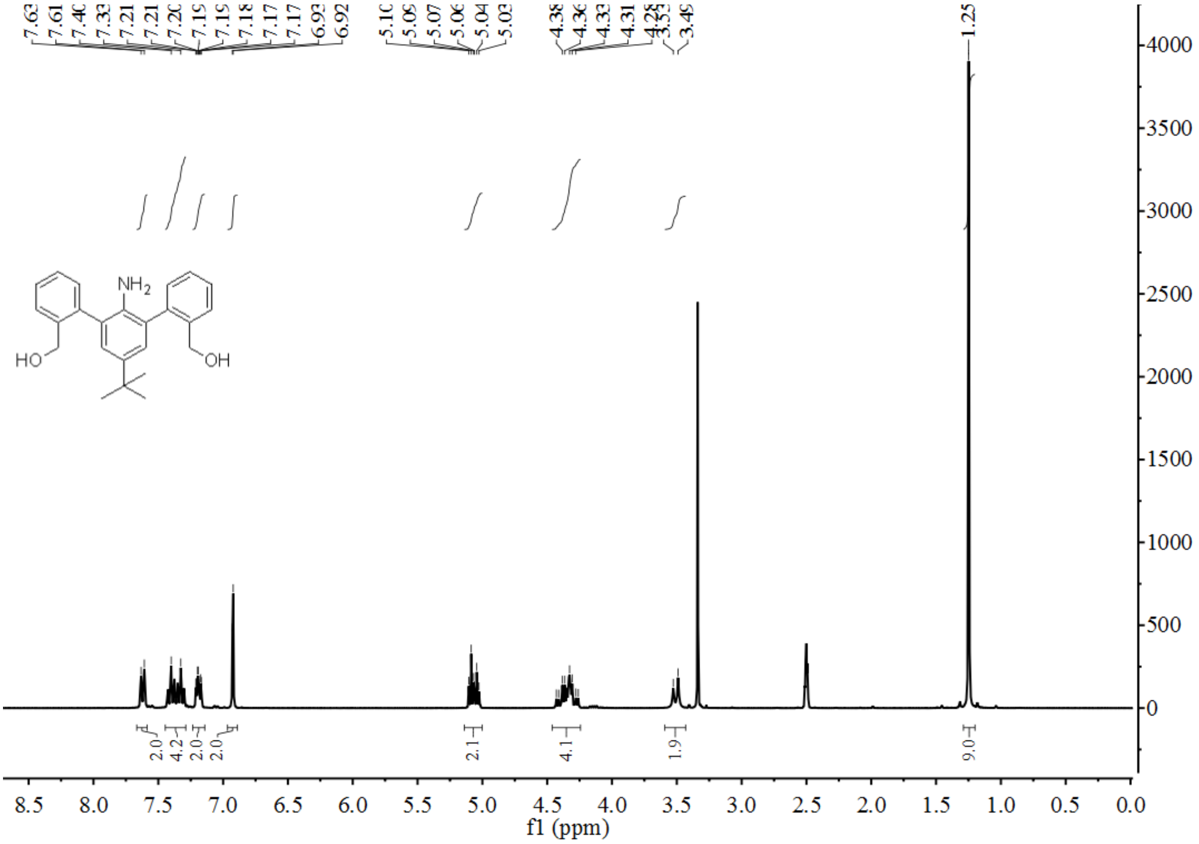

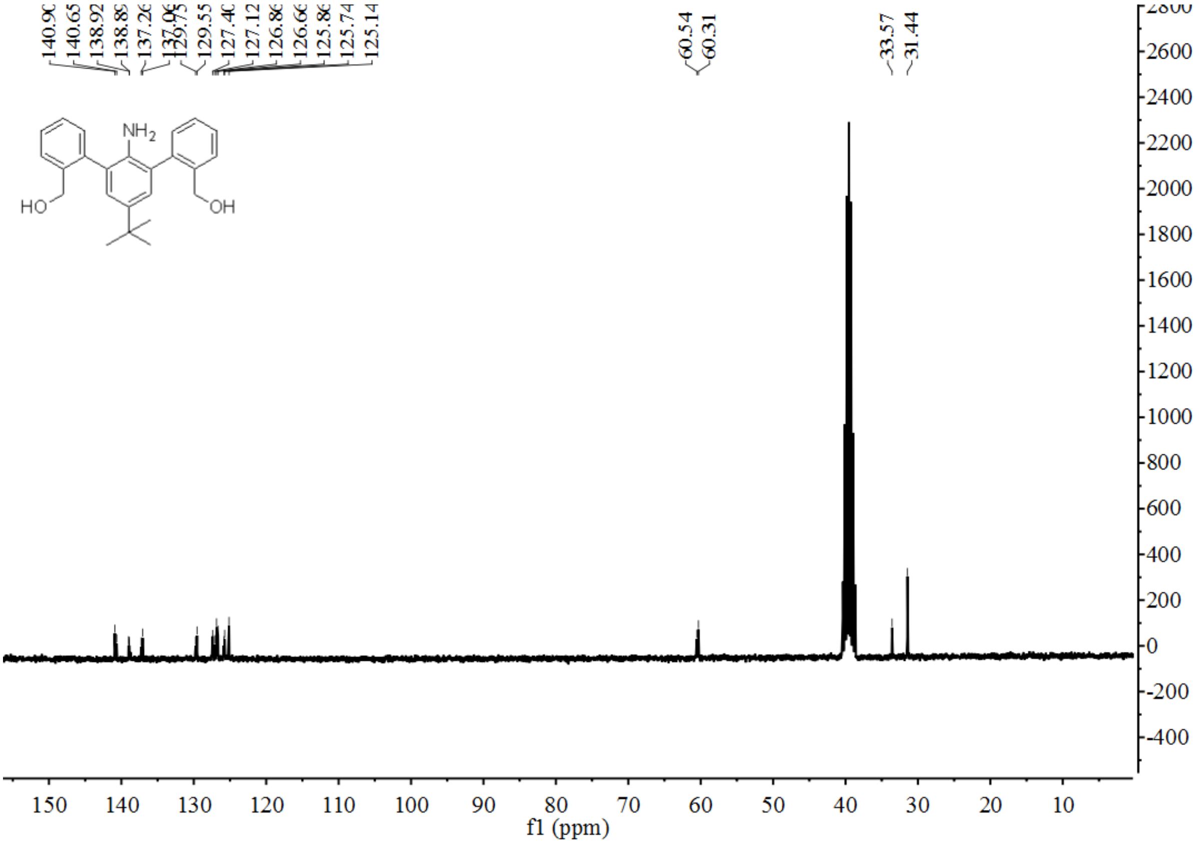

^{1}H-NMR (300 MHz, top) and ^{13}C-NMR (75 MHz, bottom) spectra of **25b** at 298 K in DMSO-d_6.

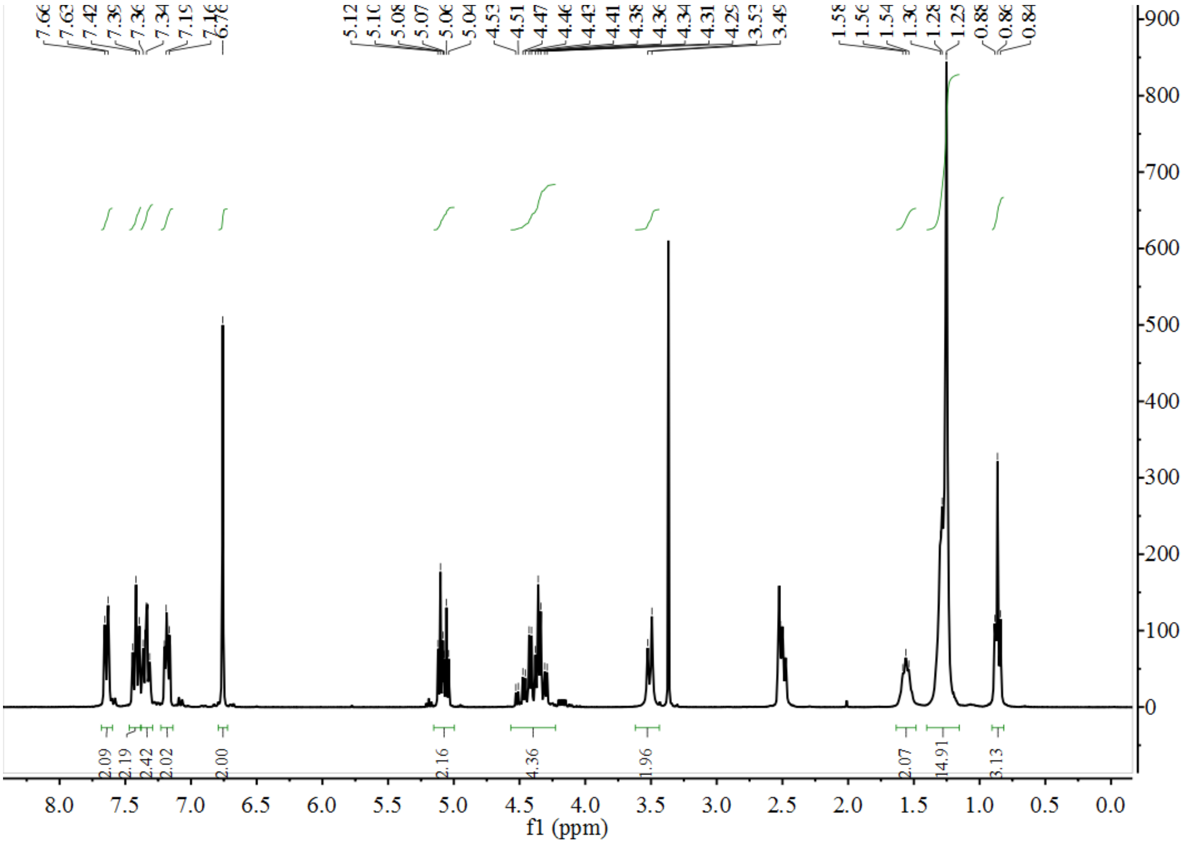

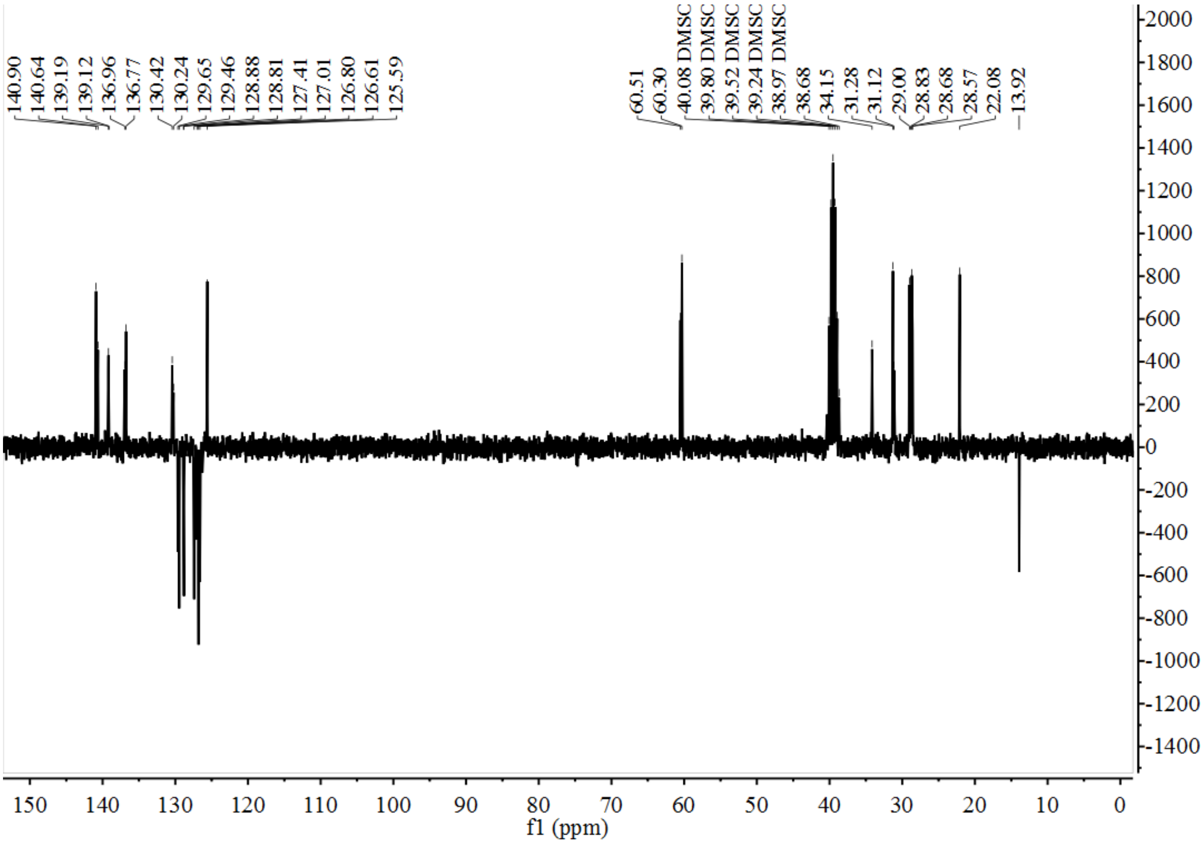

^{1}H-NMR (300 MHz, top) and ^{13}C-NMR (75 MHz, bottom) spectra of **25c** at 298 K in DMSO-d_6.

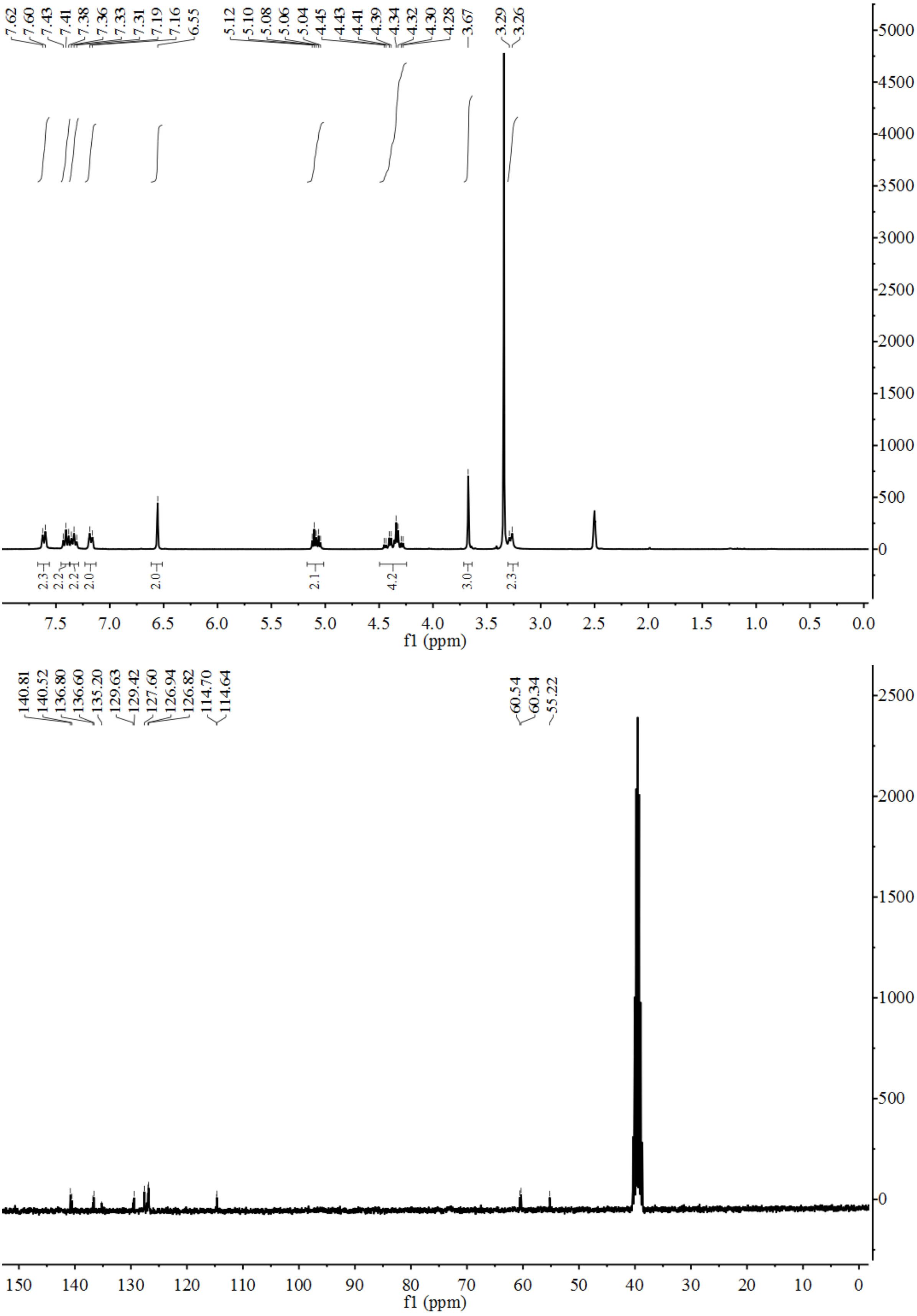

^{1}H-NMR (500 MHz, top) and ^{13}C-NMR (125 MHz, bottom) spectra of **20a** at 298 K in DMSO-d$_6$.

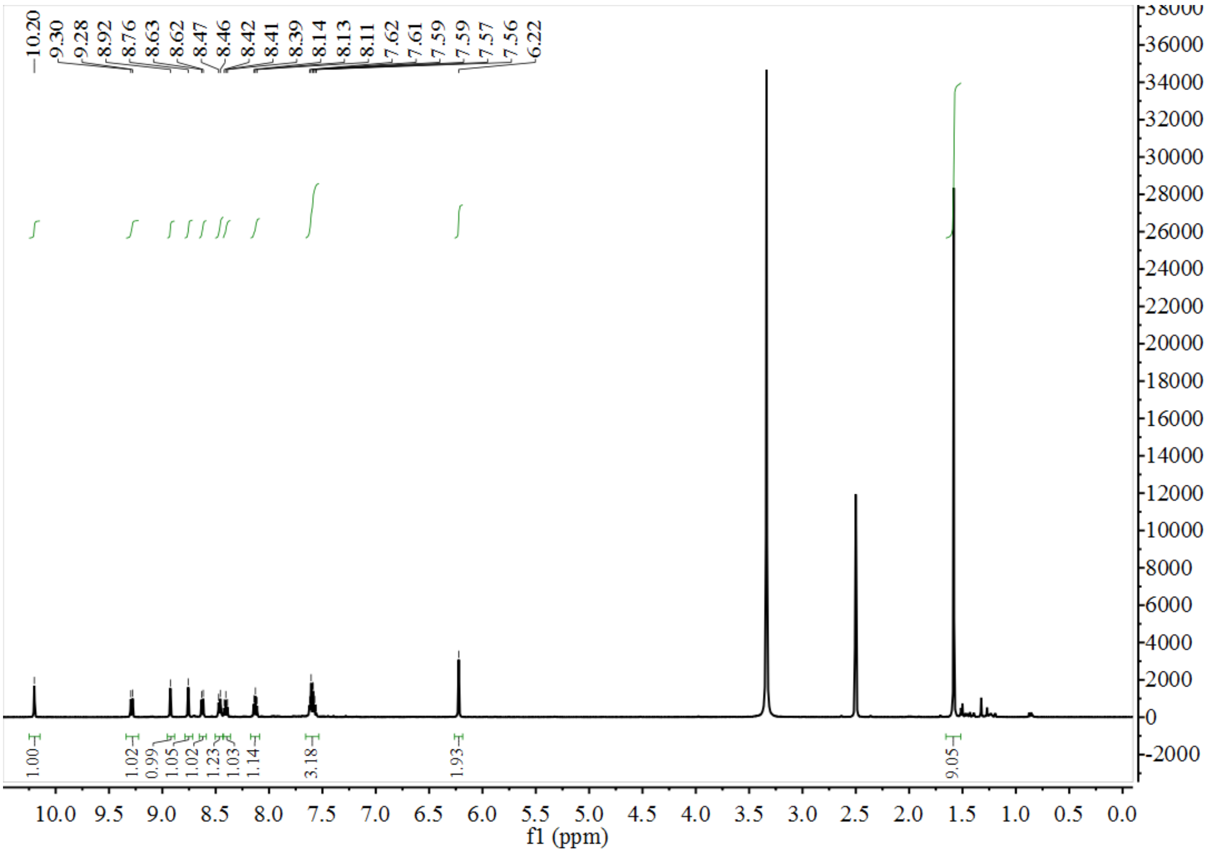

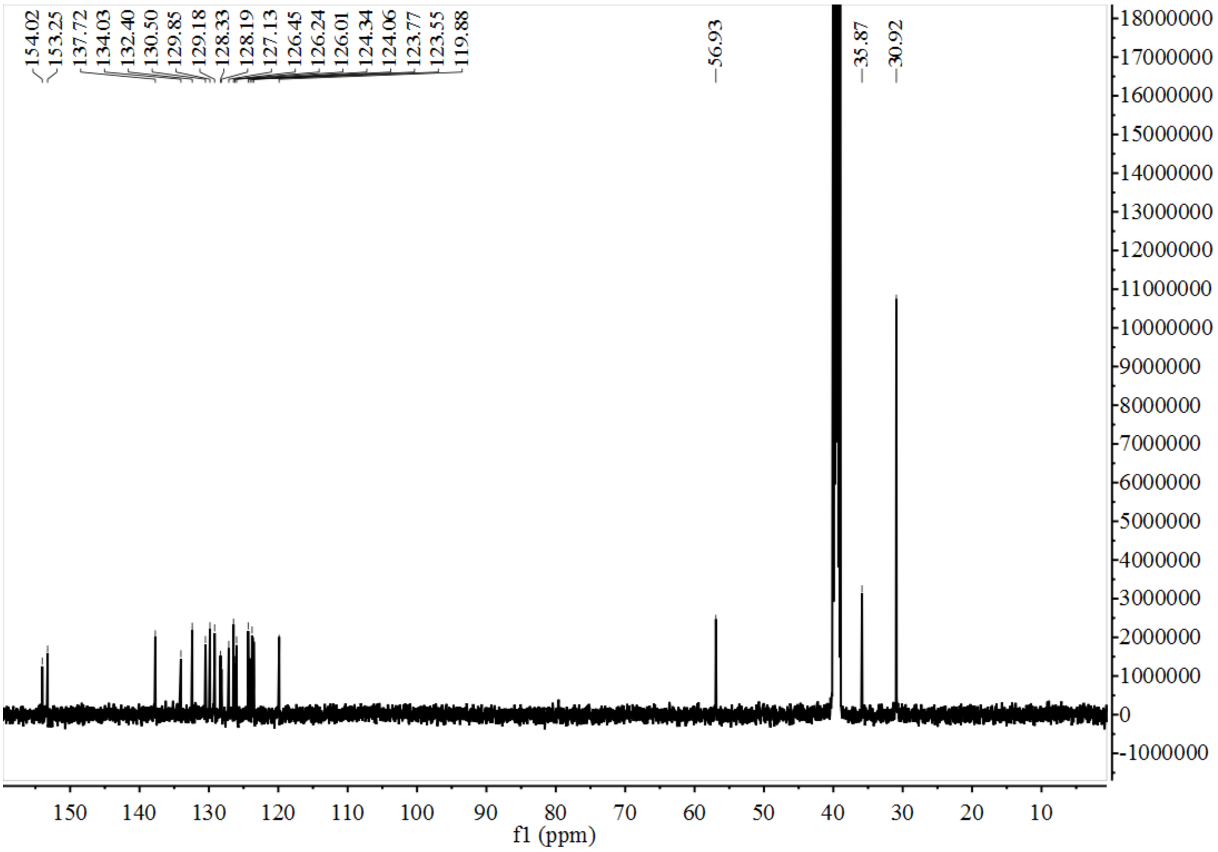

^{1}H-NMR (300 MHz, top) and ^{13}C-NMR (75 MHz, bottom) spectra of **20b** at 298 K in DMSO-d_6.

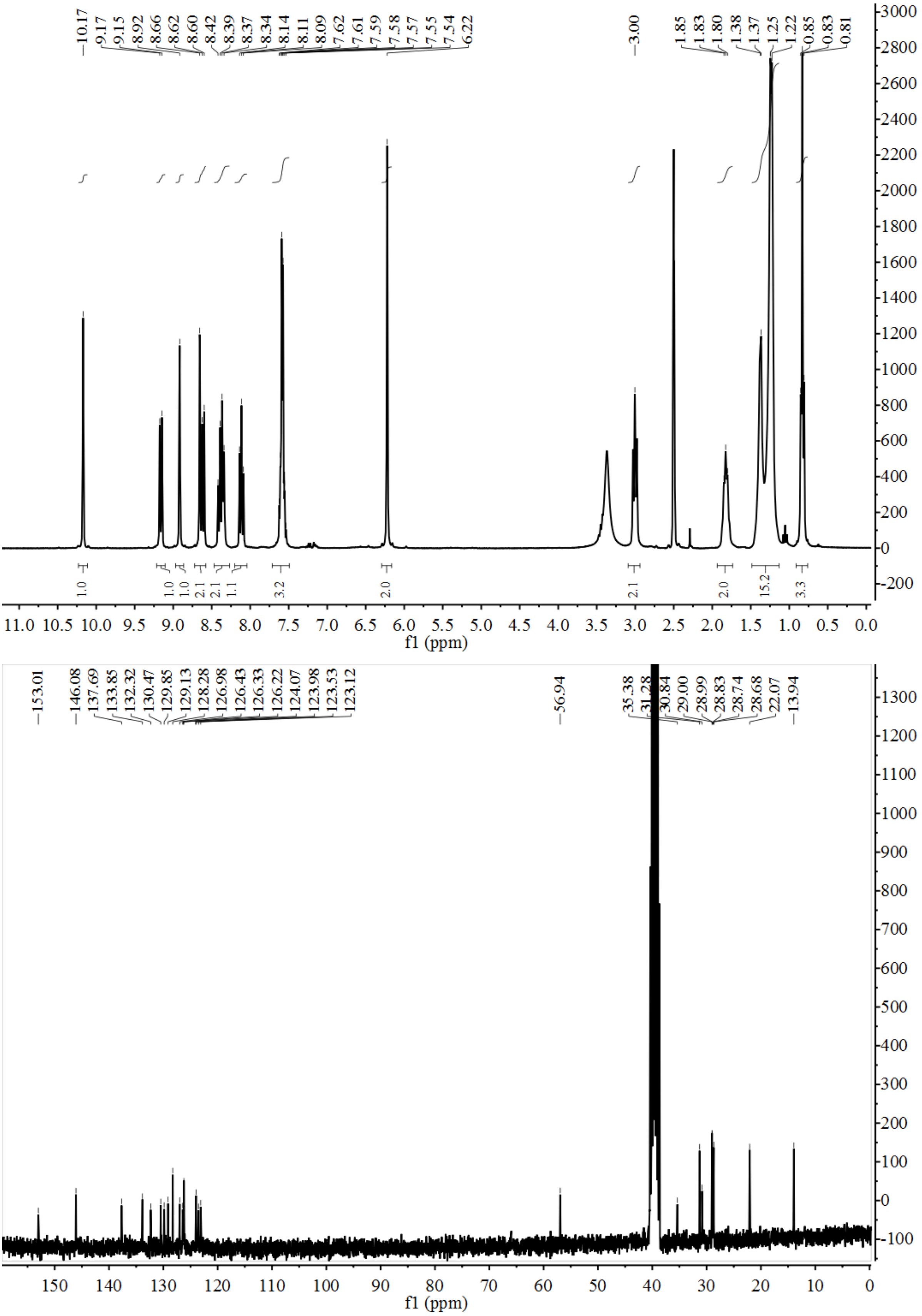

^{1}H-NMR (300 MHz, top) and ^{13}C-NMR (176 MHz, bottom) spectra of **20c** at 298 K in DMSO-d$_6$.

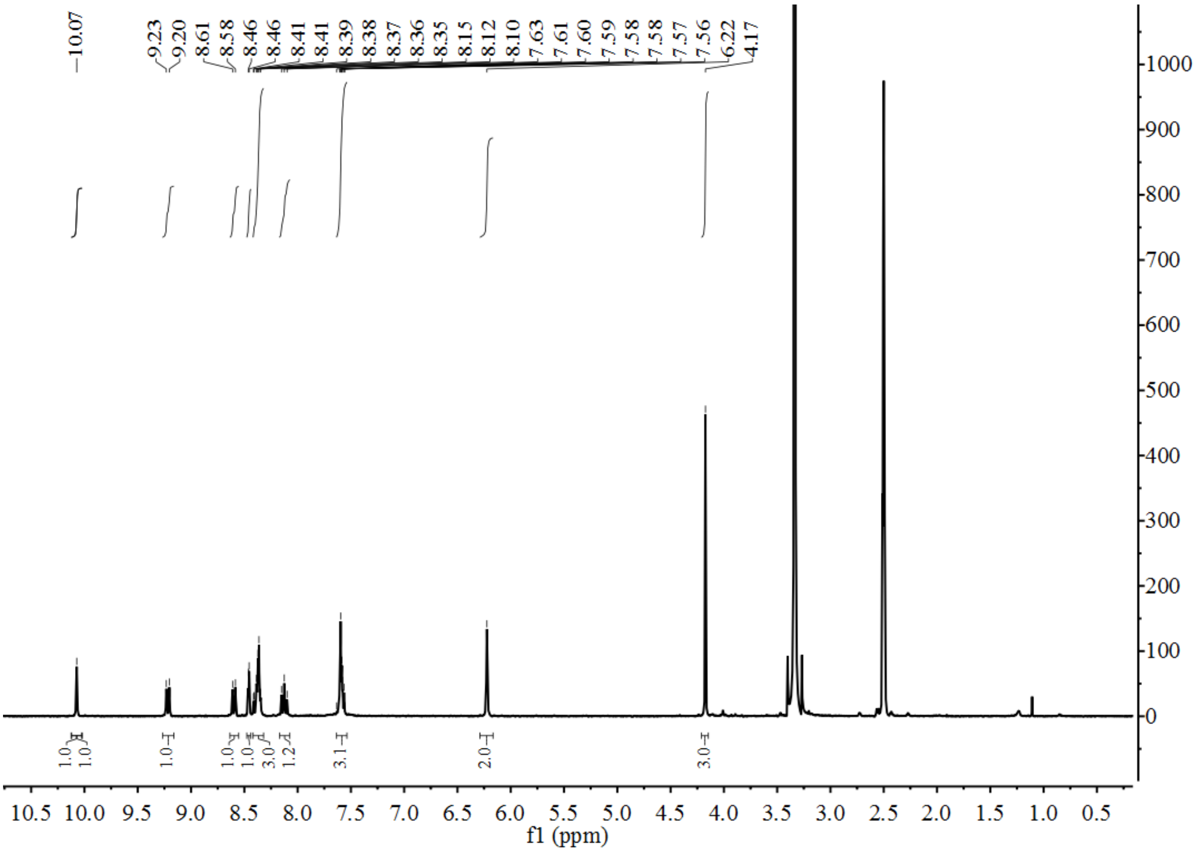

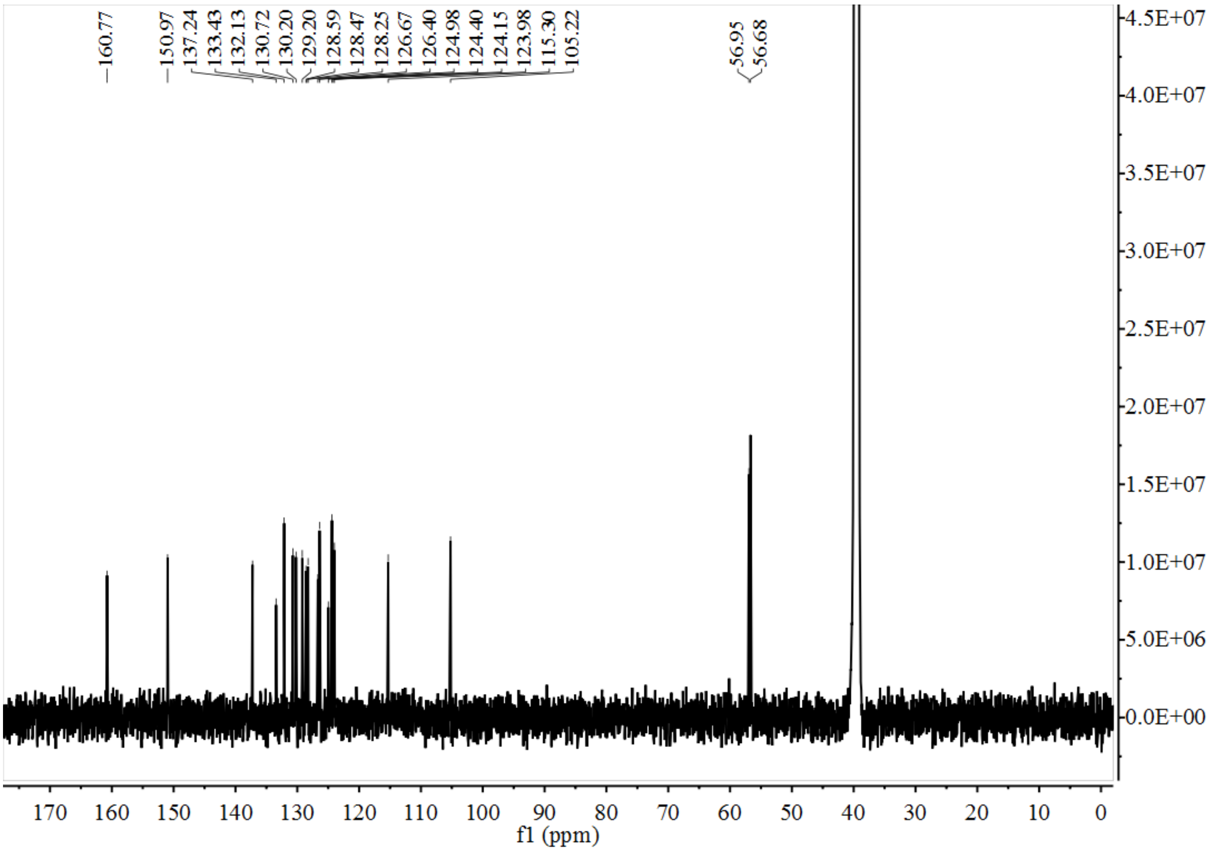

^{1}H-NMR (300 MHz, top) and ^{13}C-NMR (75 MHz, bottom) spectra of **5**(15a) at 298 K in Chloroform-d$_1$.

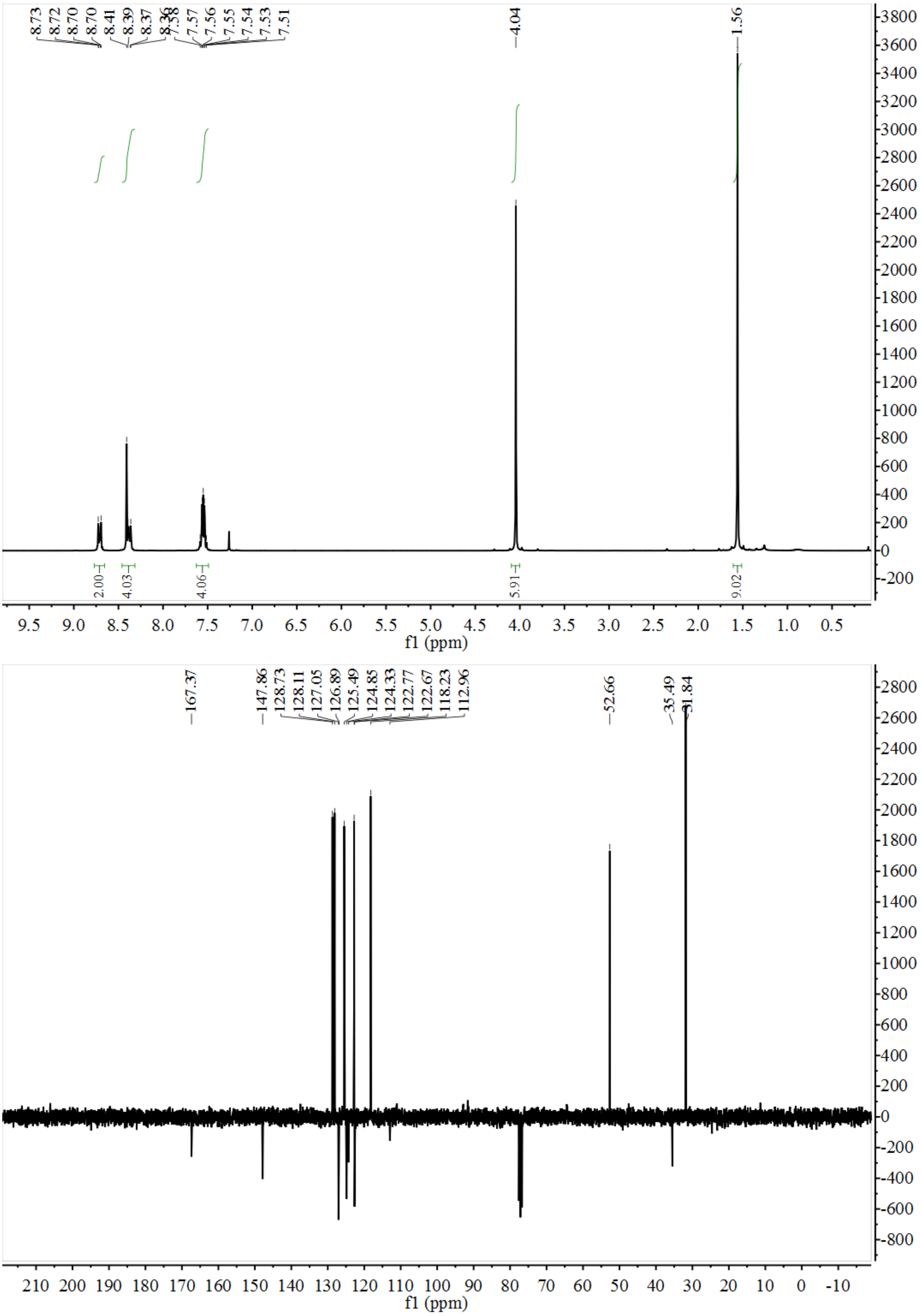

^{1}H-NMR (300 MHz, top) and ^{13}C-NMR (75 MHz, bottom) spectra of **15** at 298 K in Chloroform-d_1.

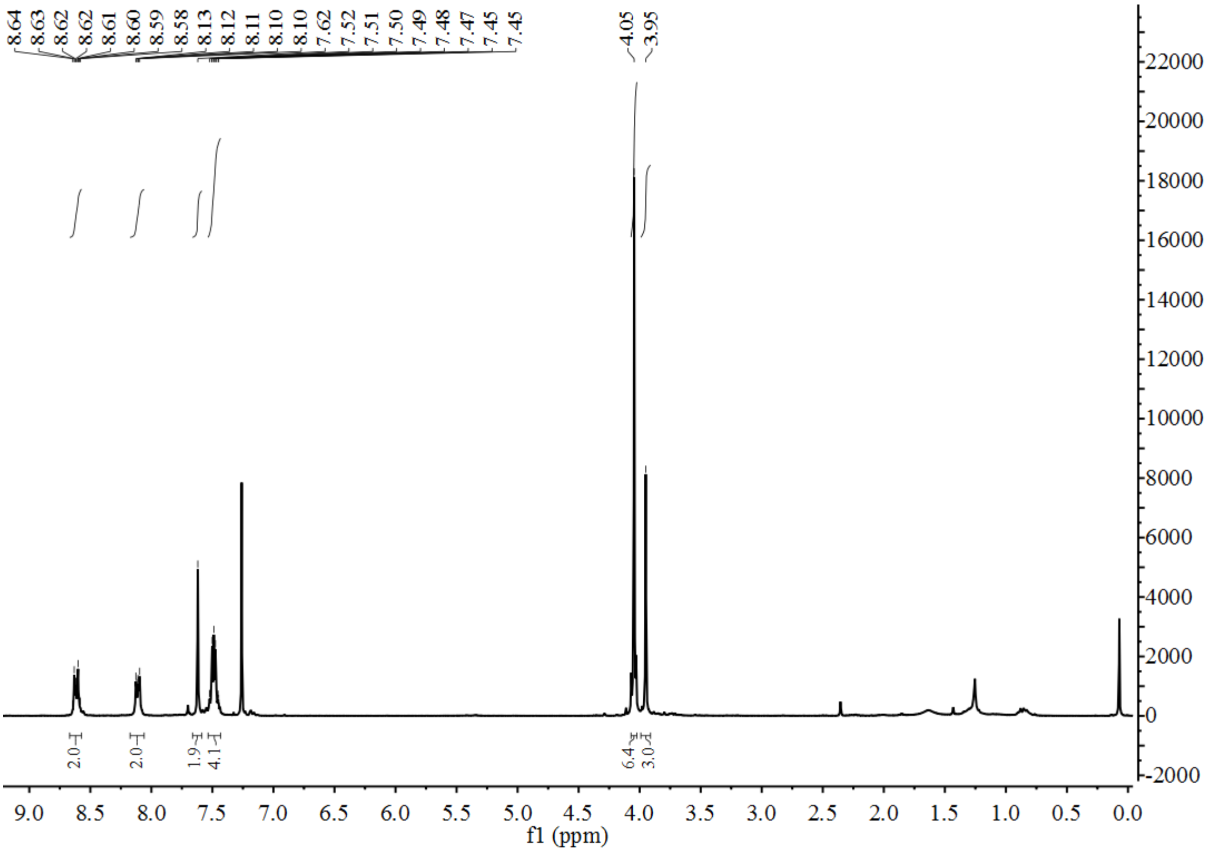

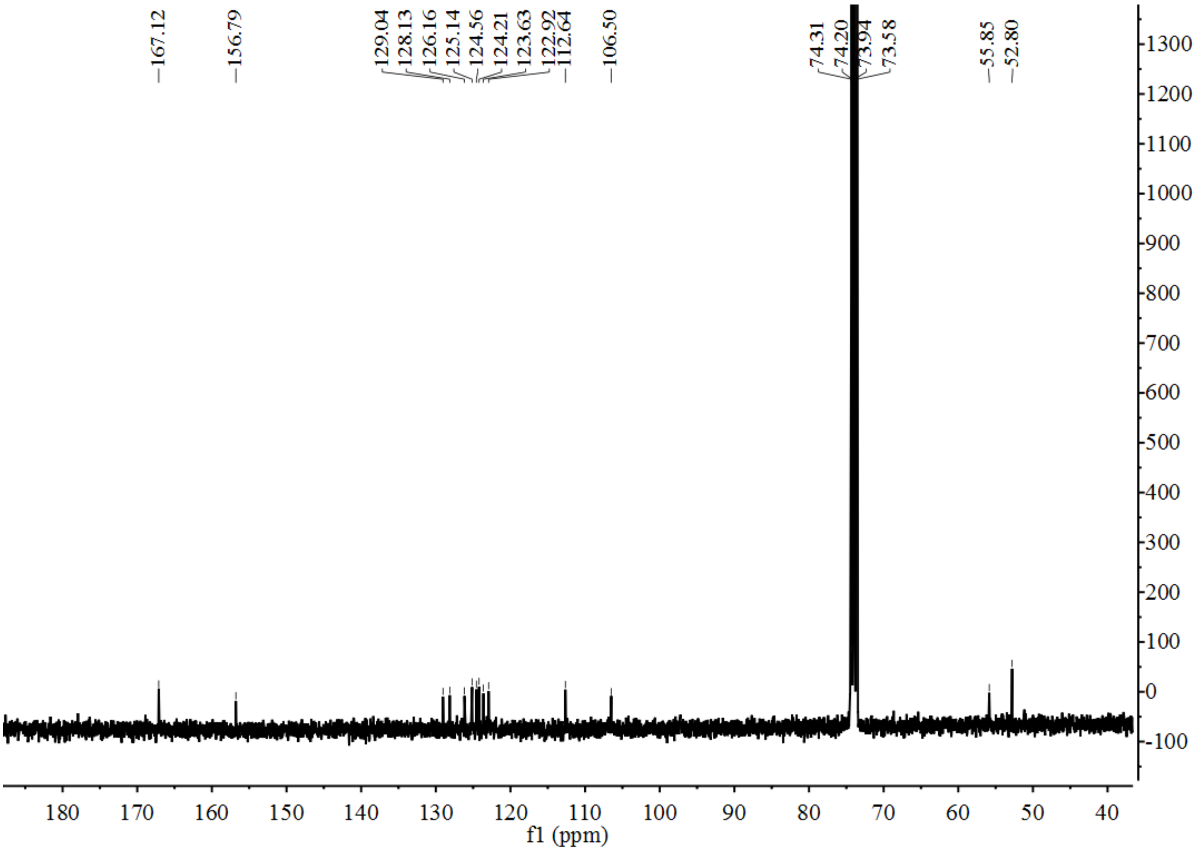

^{1}H-NMR (700 MHz, top) and ^{13}C-NMR (176 MHz, bottom) spectra of **16** at 298 K in Methylenchloride-d_2.

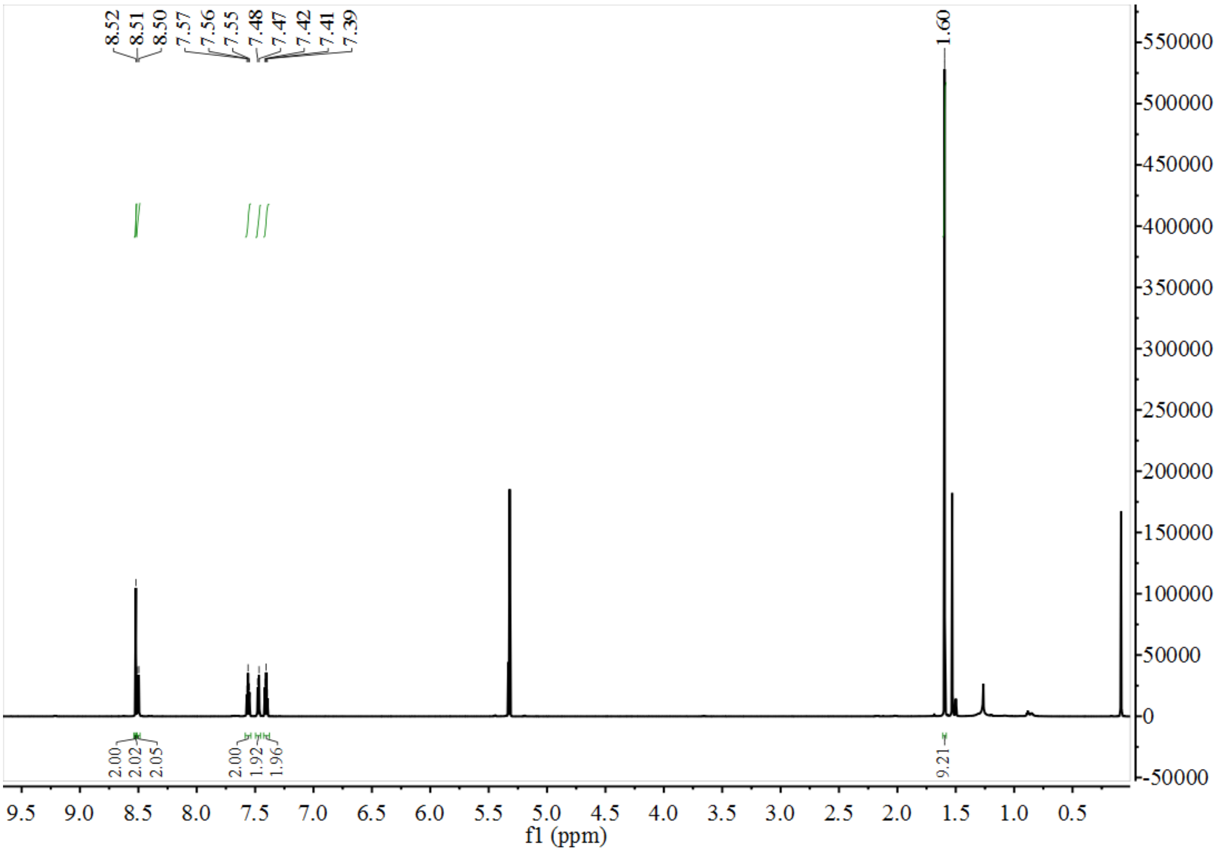

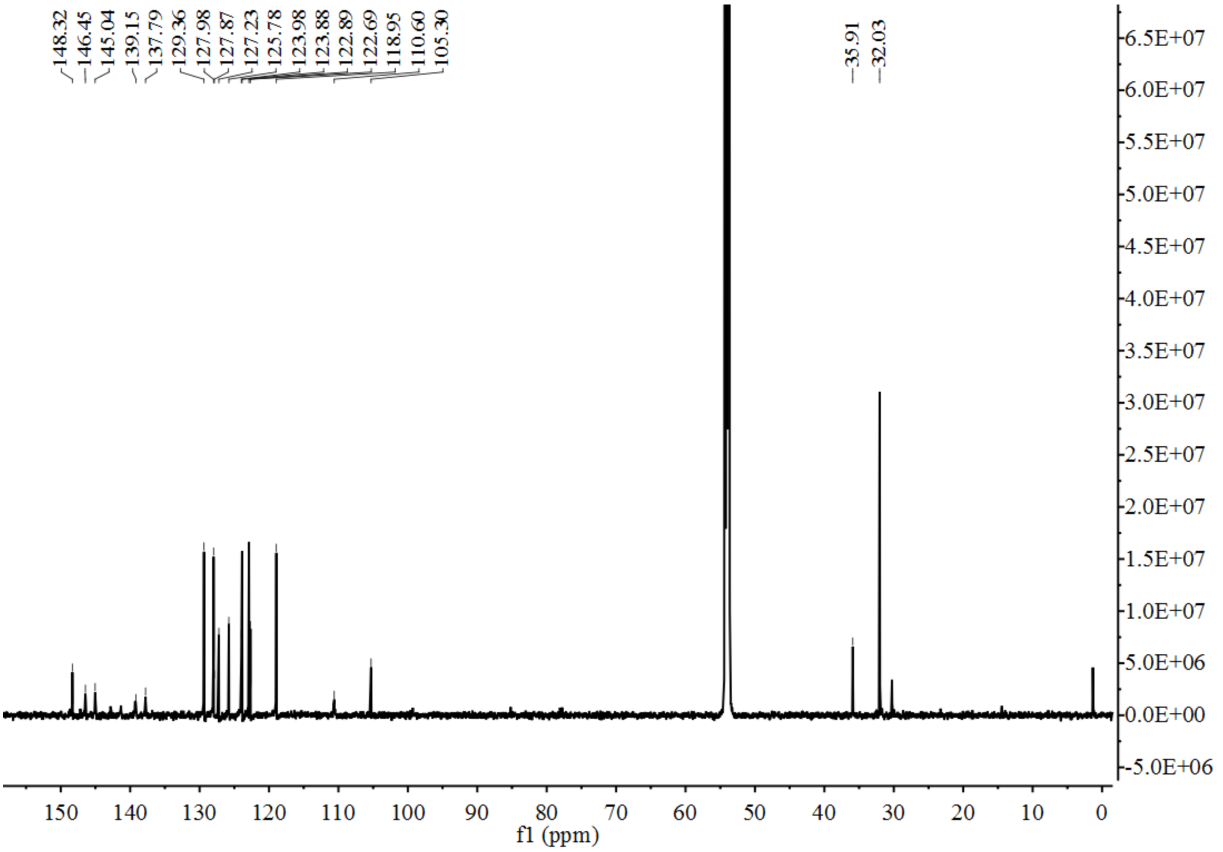

^{1}H-NMR (300 MHz, top) and ^{13}C-NMR (176 MHz, bottom) spectra of **17a**) at 298 K in Tetrachlorethane-d$_2$.

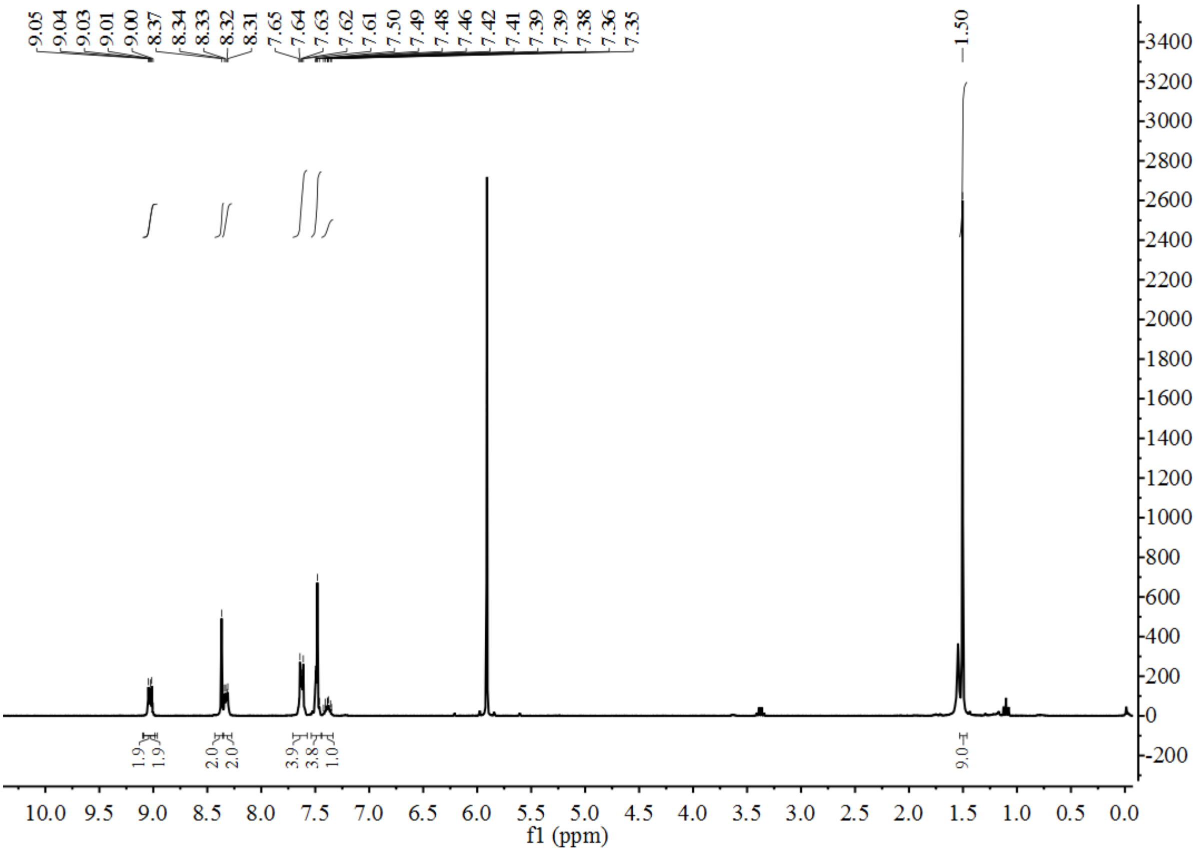

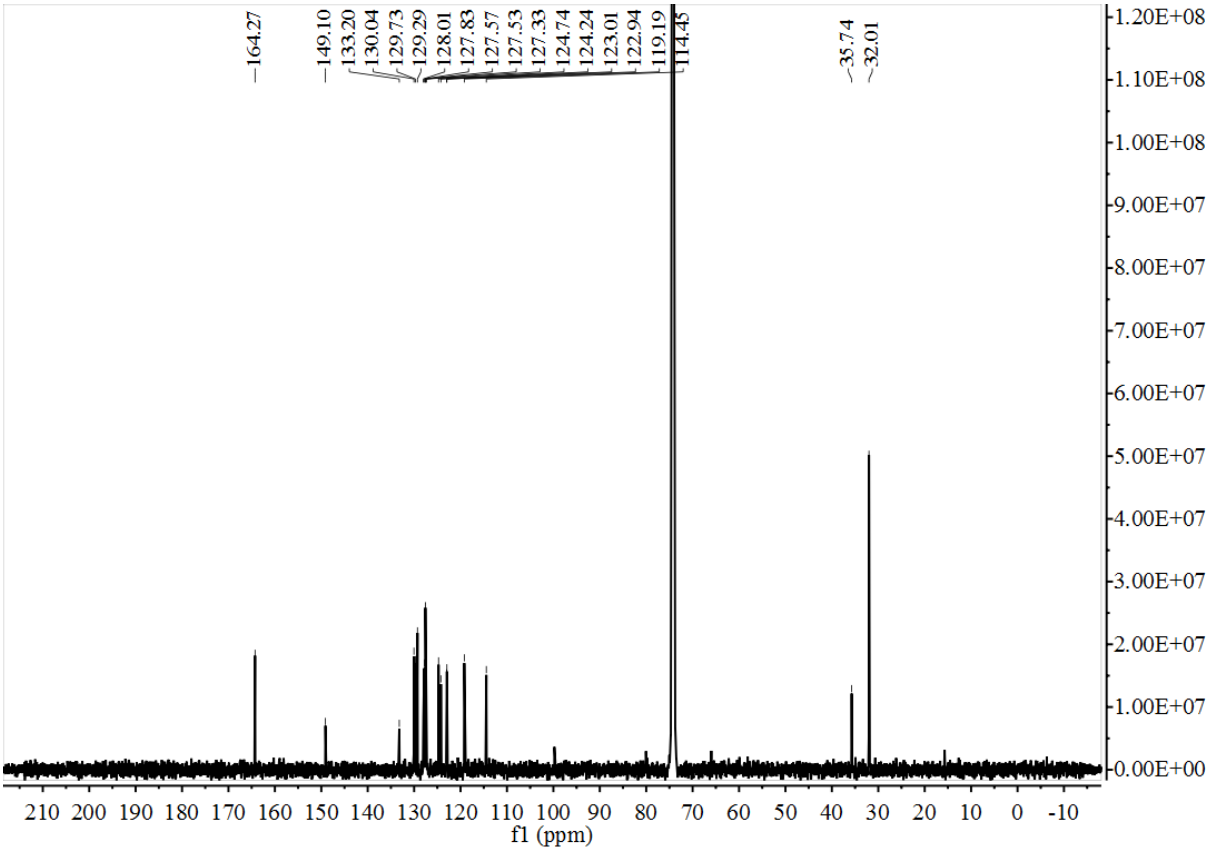

^{1}H-NMR (500 MHz, top) and ^{13}C-NMR (126 MHz, bottom) spectra of **17b**) at 393 K in Tetrachlorethane-d_2.

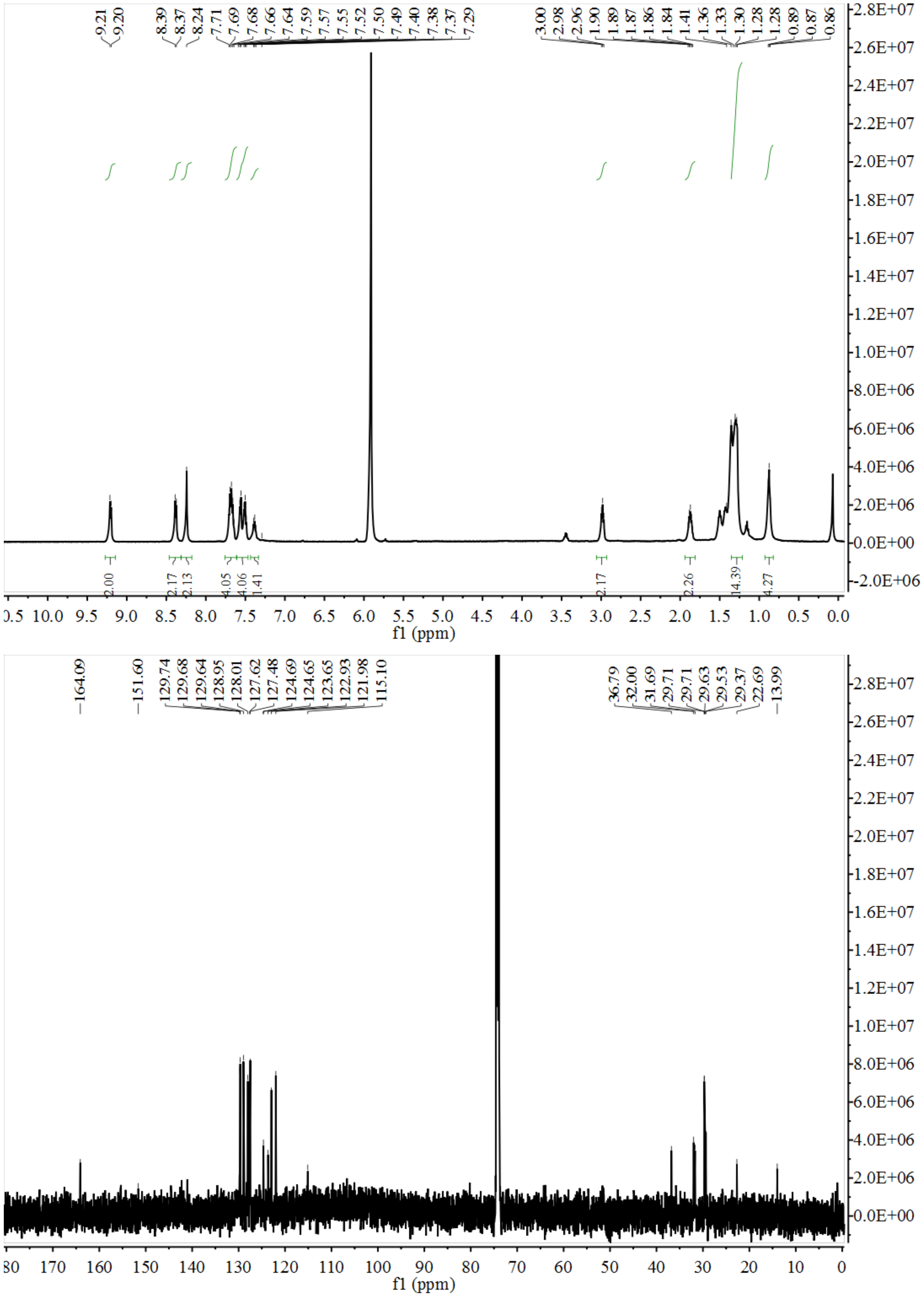

^{1}H-NMR (500 MHz, top) and ^{13}C-NMR (126 MHz, bottom) spectra of **17c**) at 373 K in Tetrachlroroethane-d_2.

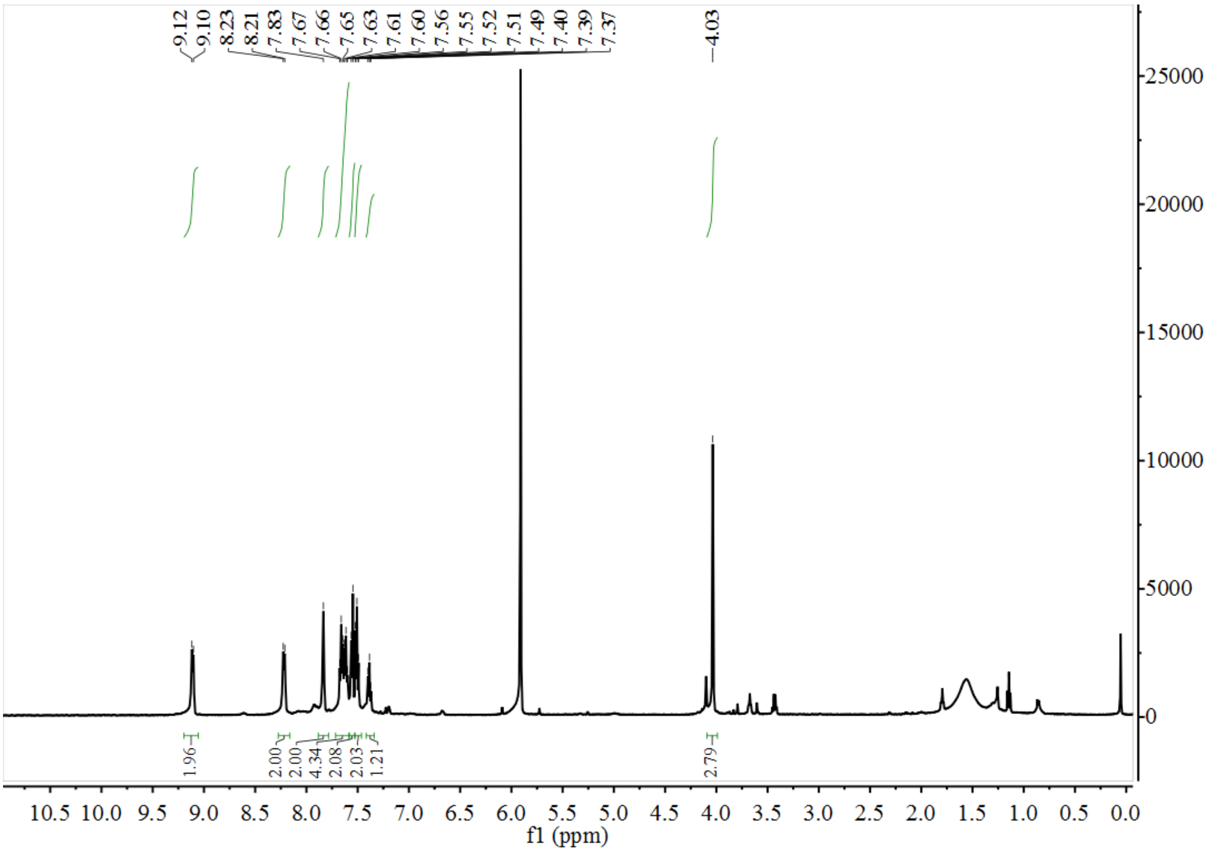

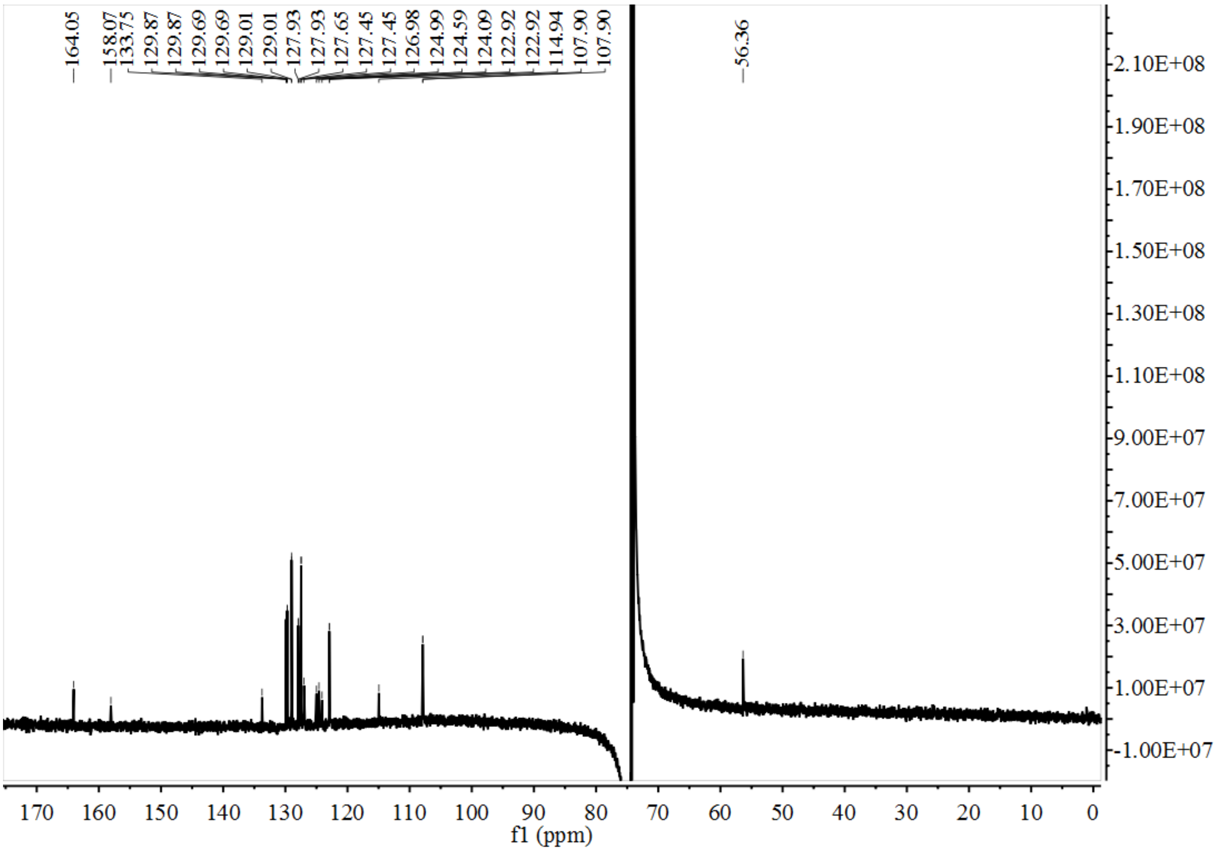

^{1}H-NMR (300 MHz, top) and ^{13}C-NMR (176 MHz, bottom) spectra of **18a**) at 298 K in Tetrachloroethane-d_2.

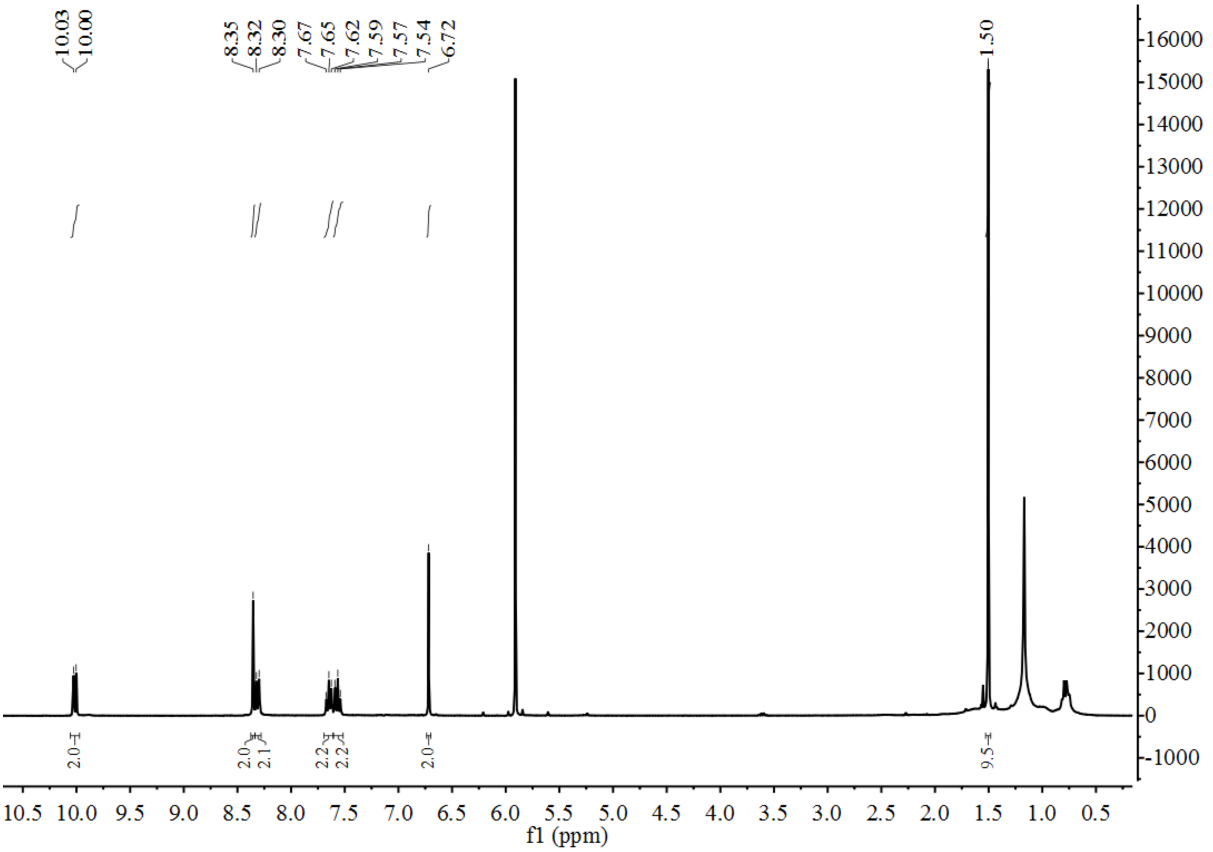

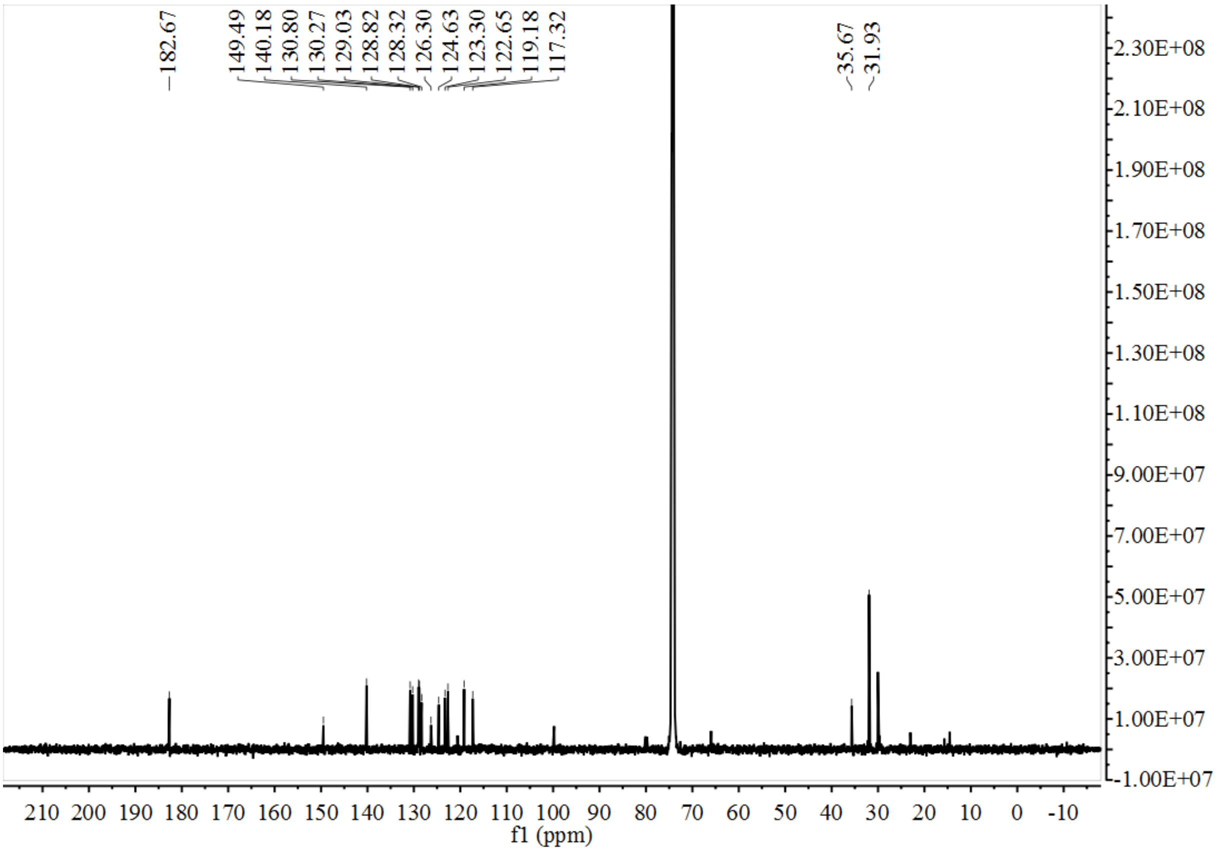

^{1}H-NMR (700 MHz, top) and ^{13}C-NMR (176 MHz, bottom) spectra of **18b**) at 298 K in Tetrachloroethane-d_2.

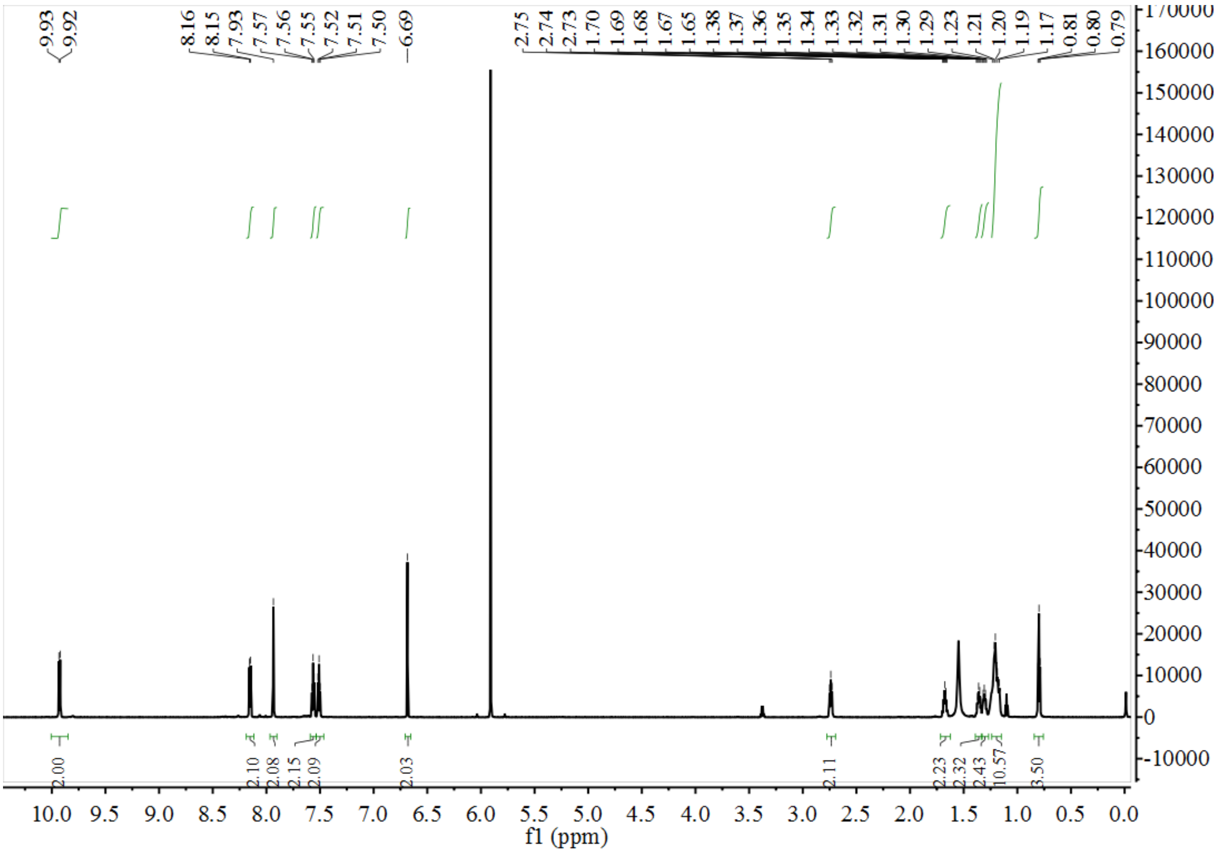

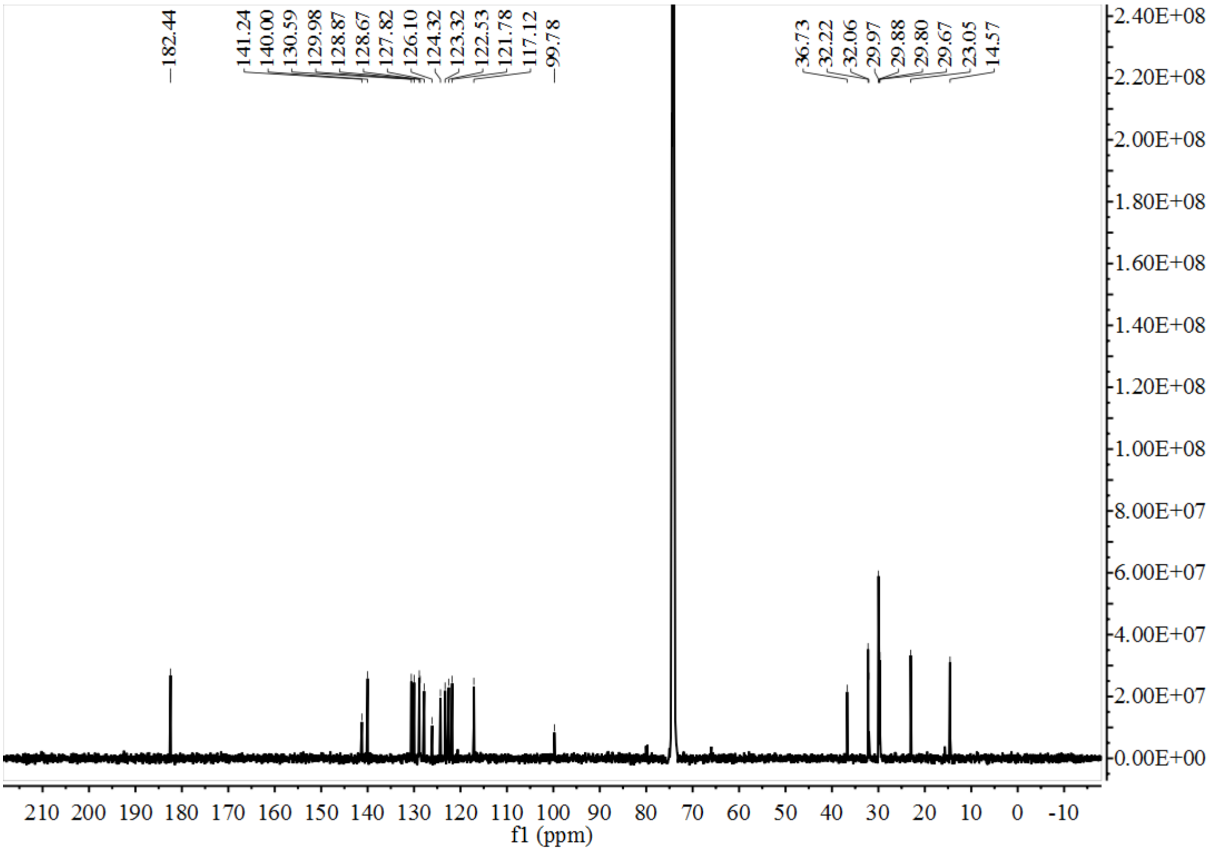

^{1}H-NMR (300 MHz, top) and ^{13}C-NMR (75 MHz, bottom) spectra of **19a**) at 298 K in Tetrachloroethane-d_2.

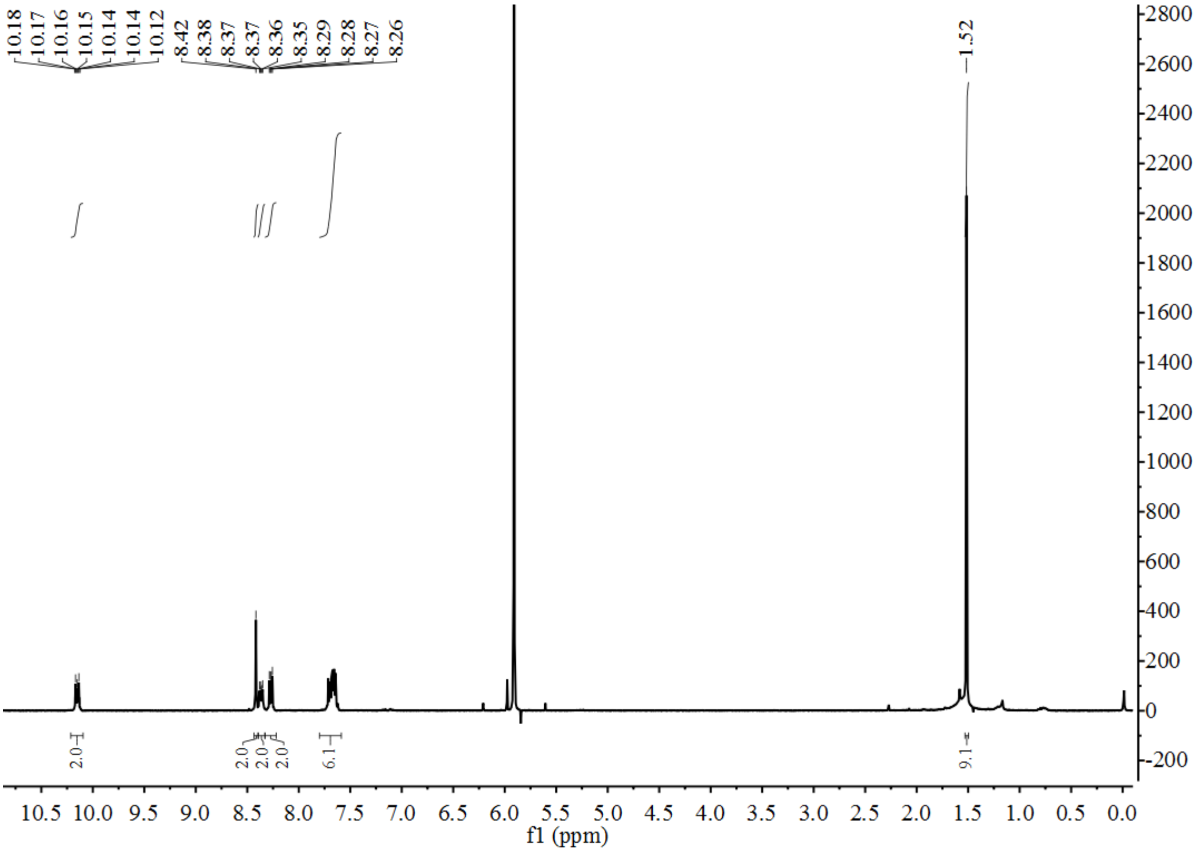

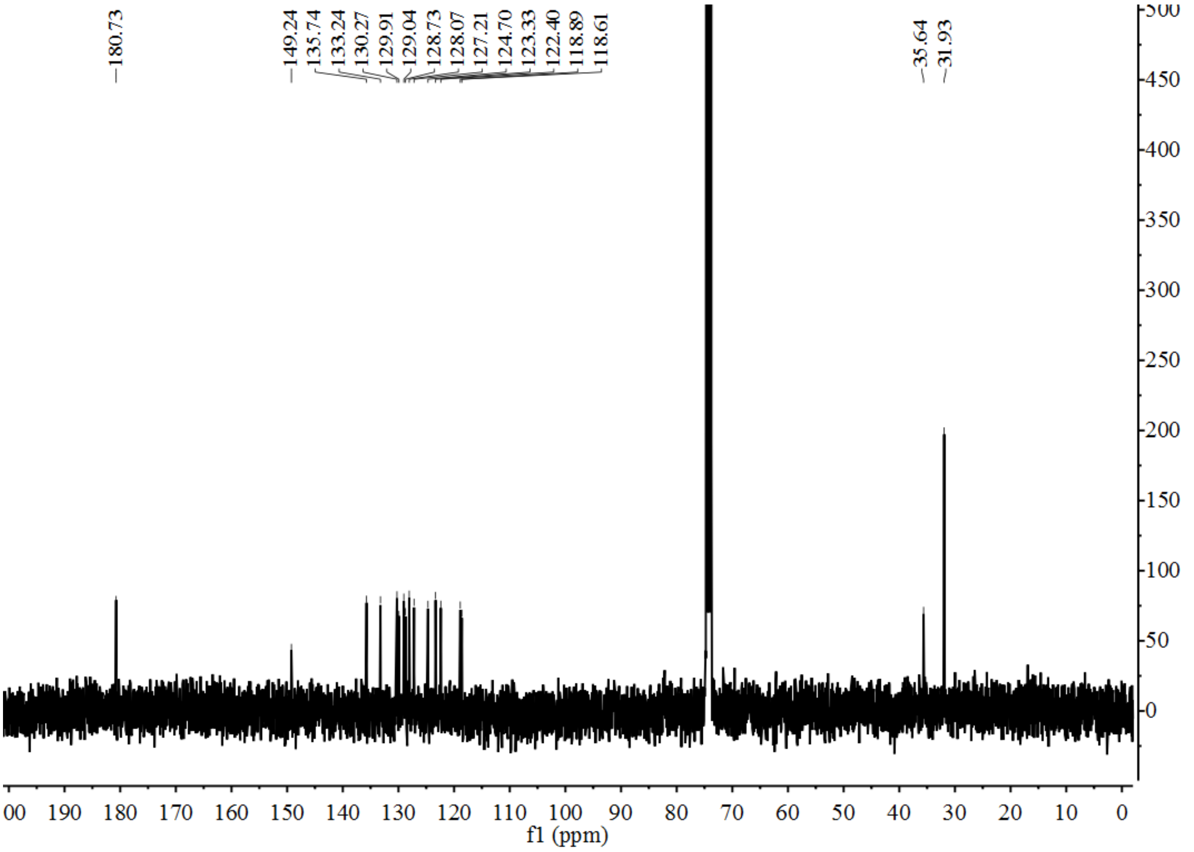

^{1}H-NMR (700 MHz, top) and ^{13}C-NMR (176 MHz, bottom) spectra of **19b**) at 323 K in Tetrachloroethane-d_2.

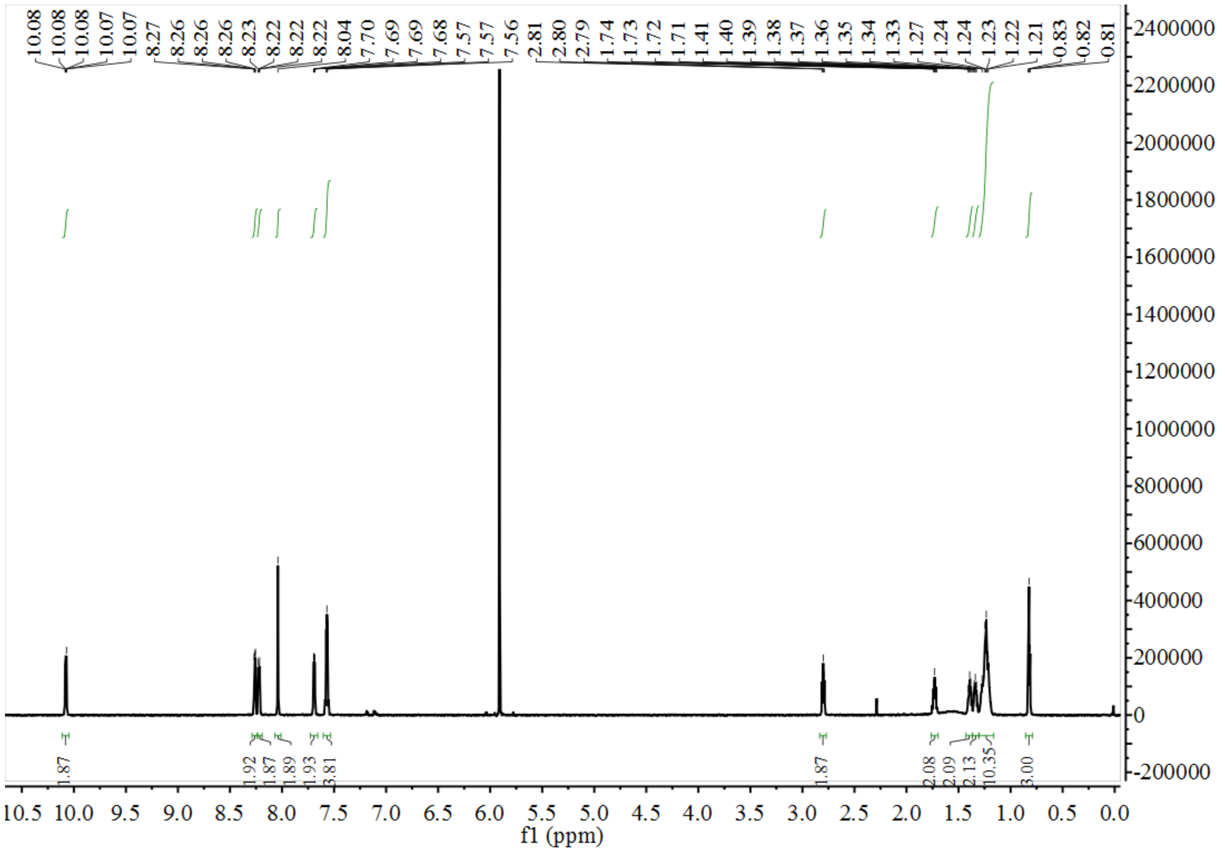

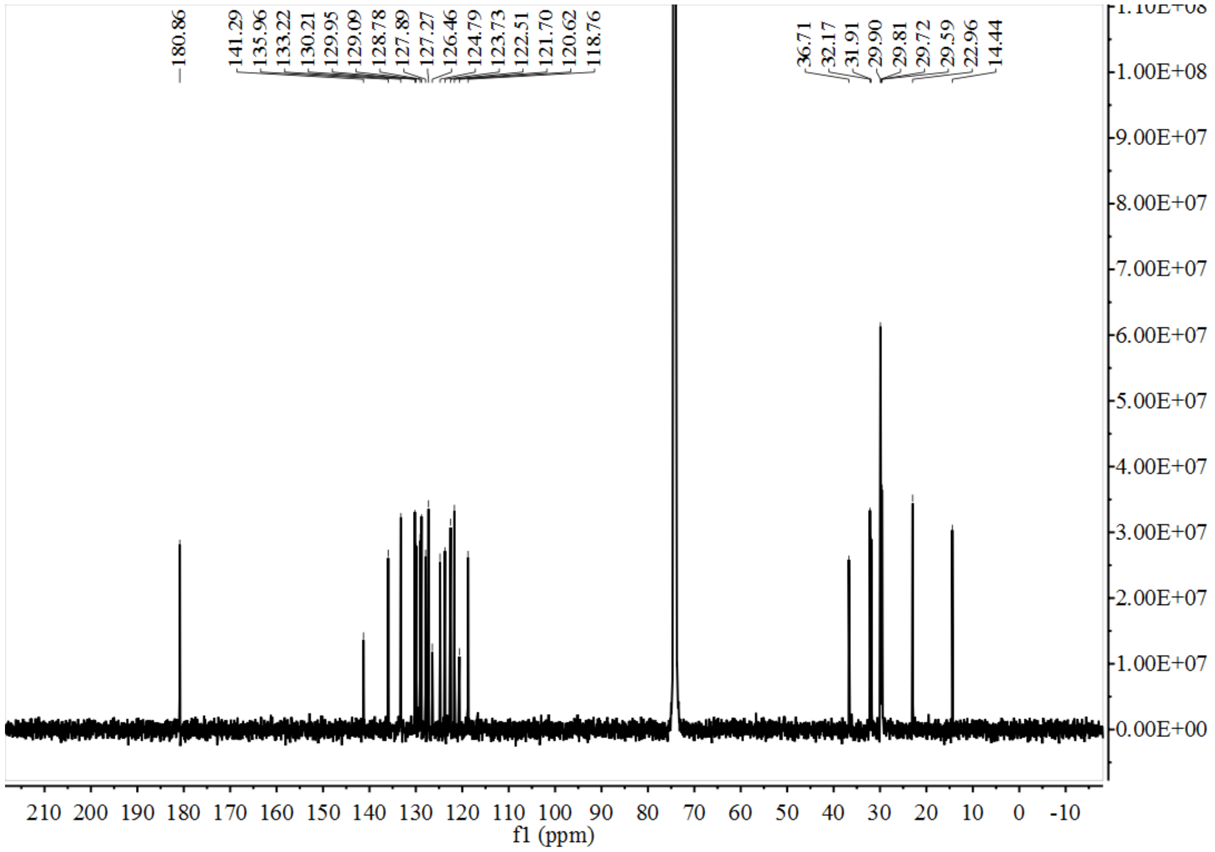

^{1}H-NMR (500 MHz, top) and ^{13}C-NMR (126 MHz, bottom) spectra of **21a**) at 403 K in Tetrachloroethane-d_2.

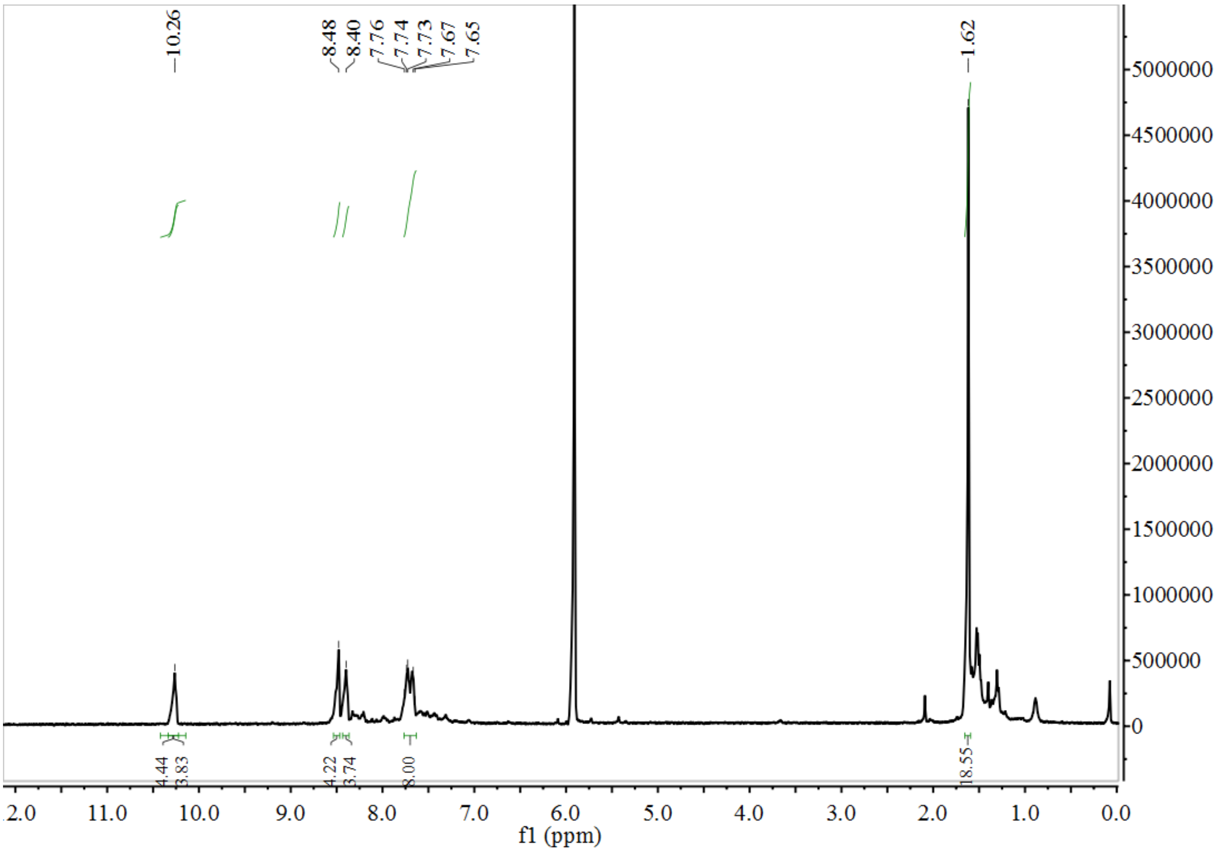

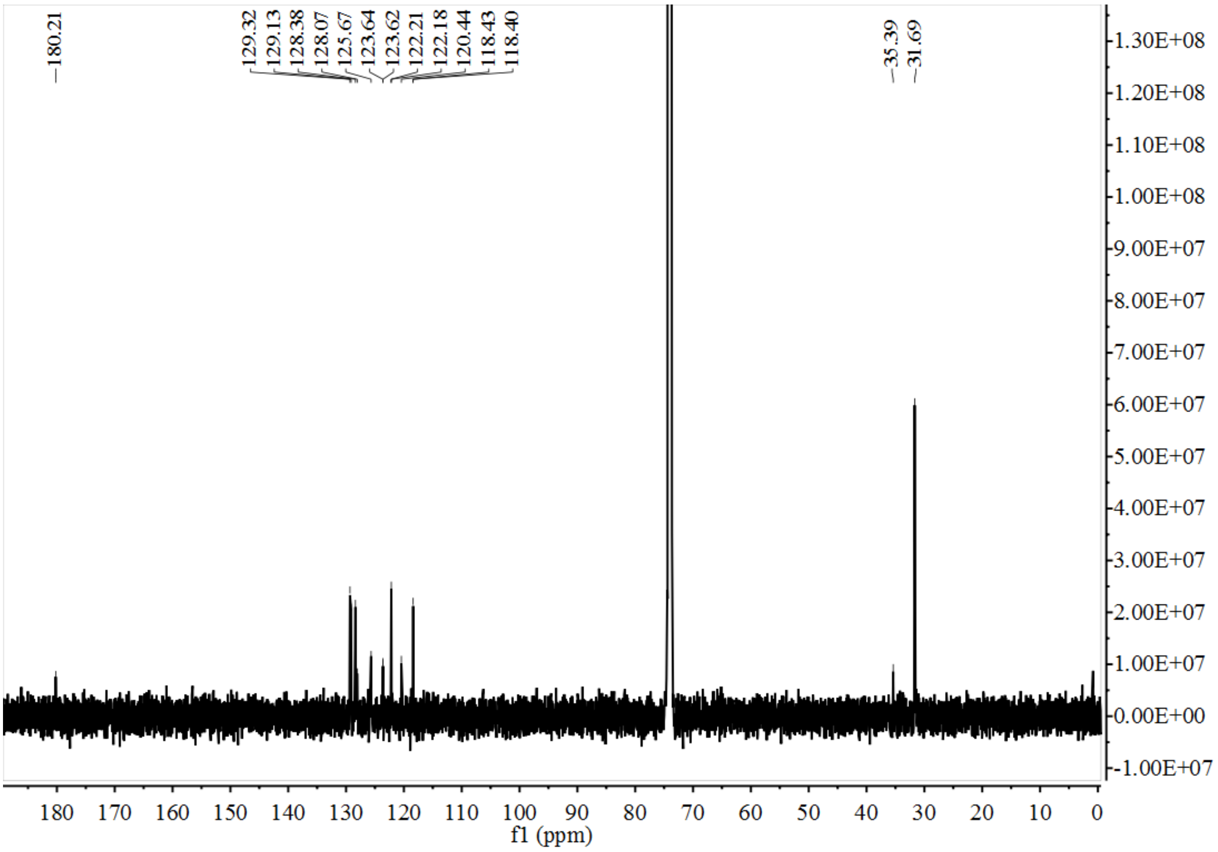

^{1}H-NMR (500 MHz, top) and ^{13}C-NMR (126 MHz, bottom) spectra of **21b**) at 393 K in Tetrachloroethane-d_2.

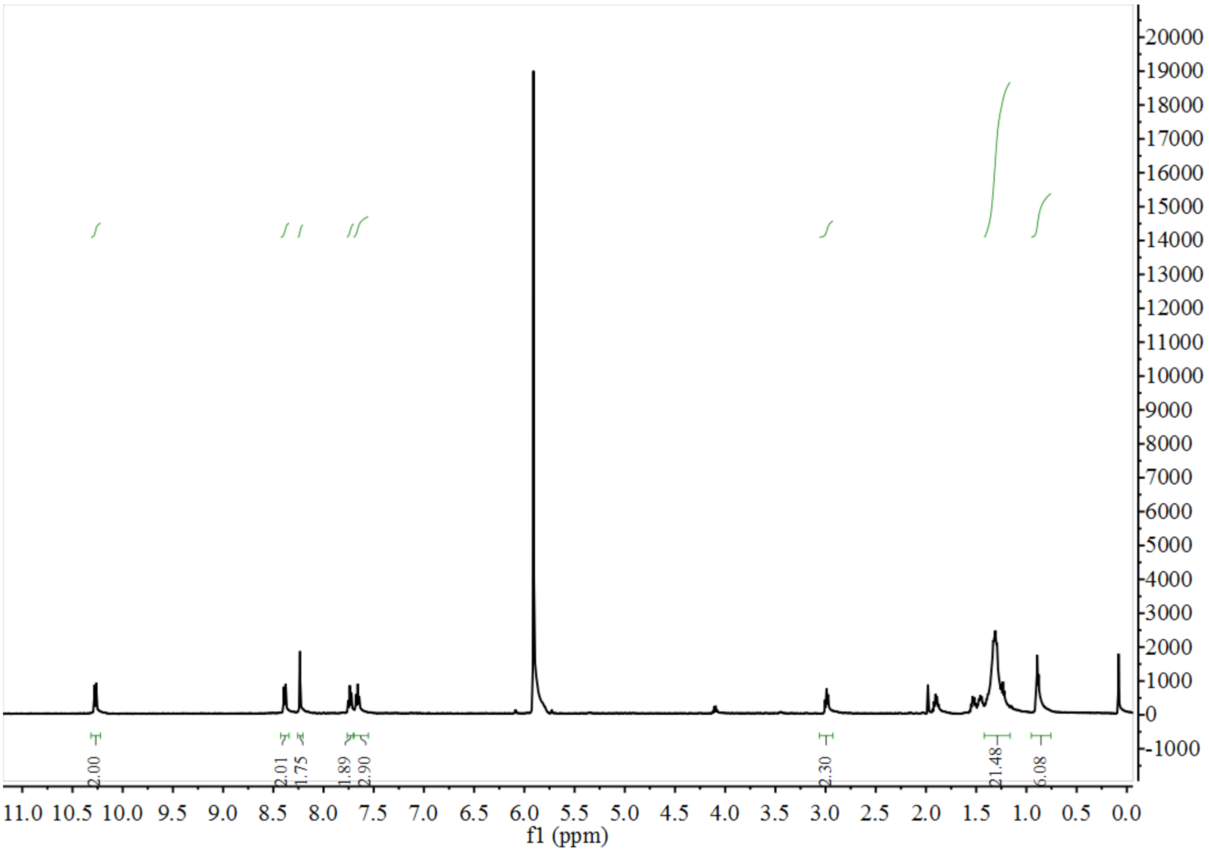

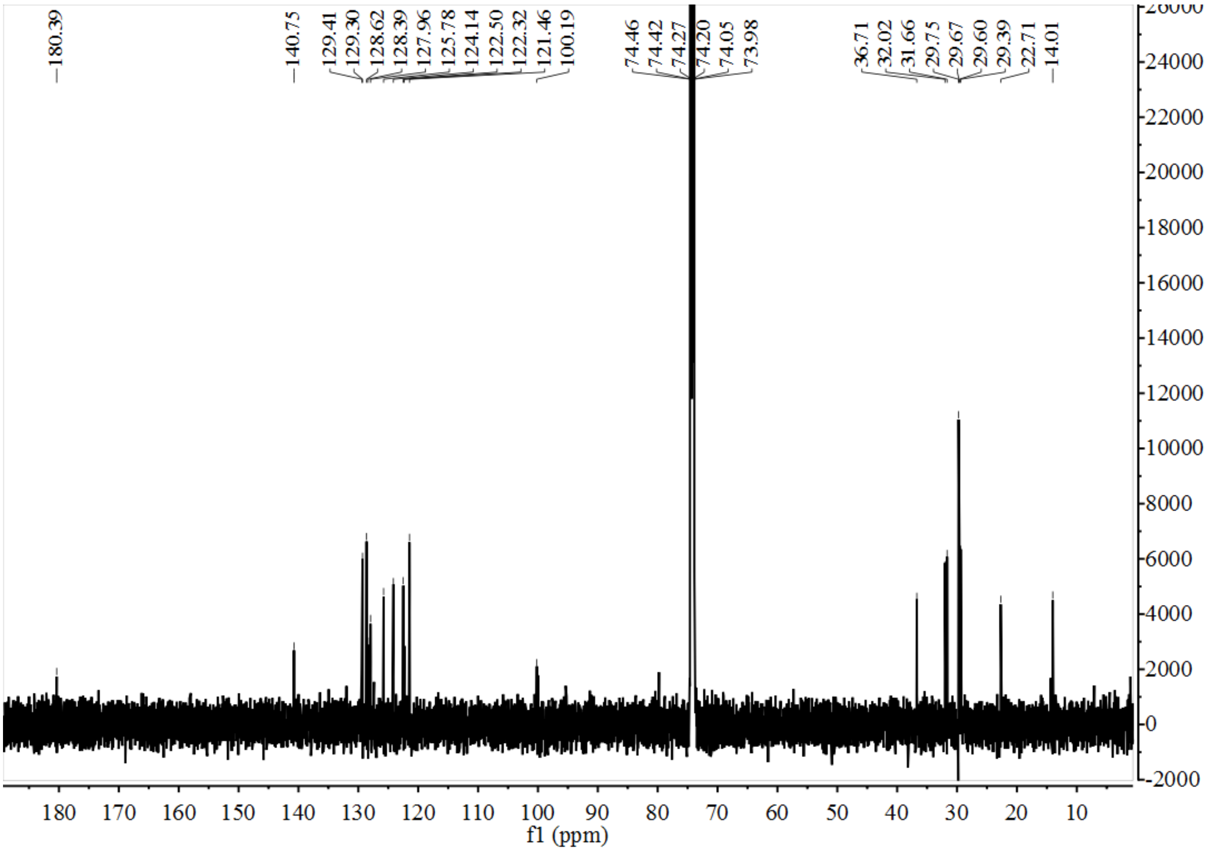

7) References

1. G. M. Sheldrick, *Acta Crystallographica Section A*, 2008, **64**, 112-122.
2. A. Altomare, M. C. Burla, M. Camalli, G. L. Cascarano, C. Giacovazzo, A. Guagliardi, A. G. G. Moliterni, G. Polidori and R. Spagna, *Journal of Applied Crystallography*, 1999, **32**, 115-119.
3. A. Spek, *Acta Crystallographica Section D*, 2009, **65**, 148-155.
4. M. J. Frisch, G. W. Trucks, H. B. Schlegel, G. E. Scuseria, M. A. Robb, J. R. Cheeseman, G. Scalmani, V. Barone, B. Mennucci, G. A. Petersson, H. Nakatsuji, M. Caricato, X. Li, H. P. Hratchian, A. F. Izmaylov, J. Bloino, G. Zheng, J. L. Sonnenberg, M. Hada, M. Ehara, K. Toyota, R. Fukuda, J. Hasegawa, M. Ishida, T. Nakajima, Y. Honda, O. Kitao, H. Nakai, T. Vreven, J. A. Montgomery Jr., J. E. Peralta, F. Ogliaro, M. J. Bearpark, J. Heyd, E. N. Brothers, K. N. Kudin, V. N. Staroverov, R. Kobayashi, J. Normand, K. Raghavachari, A. P. Rendell, J. C. Burant, S. S. Iyengar, J. Tomasi, M. Cossi, N. Rega, N. J. Millam, M. Klene, J. E. Knox, J. B. Cross, V. Bakken, C. Adamo, J. Jaramillo, R. Gomperts, R. E. Stratmann, O. Yazyev, A. J. Austin, R. Cammi, C. Pomelli, J. W. Ochterski, R. L. Martin, K. Morokuma, V. G. Zakrzewski, G. A. Voth, P. Salvador, J. J. Dannenberg, S. Dapprich, A. D. Daniels, Ö. Farkas, J. B. Foresman, J. V. Ortiz, J. Cioslowski and D. J. Fox, Gaussian, Inc., Wallingford, CT, USA, 2009.
5. E. R. Davidson and D. Feller, *Chemical Reviews*, 1986, **86**, 681-696.
